# TGFβ signaling promotes cell cycle progression and resistance to the CDK4/6 inhibitor palbociclib through SOX4 transcriptional modulation in breast cancer cells

**DOI:** 10.1038/s41419-026-08435-4

**Published:** 2026-02-04

**Authors:** Mohamad Moustafa Ali, Yuka Itoh, Aisha Mariama Pereira Badji, Sarah Gallant, Chrysoula Tsirigoti, Yu Bai, Beata Filipek-Górniok, Keiji Miyazawa, Carl-Henrik Heldin, Aristidis Moustakas

**Affiliations:** 1 Department of Medical Biochemistry and Microbiology, Science for Life Laboratory, Uppsala University, Uppsala, Sweden; 2 Department of Biochemistry, Graduate School of Medicine, University of Yamanashi, Yamanashi, Japan; 3 Astra Zeneca, Mölndal, Sweden; 4 Department of Genetics and Pathology, Science for Life Laboratory, Uppsala University, Uppsala, Sweden

**Keywords:** Breast cancer, Cancer microenvironment

## Abstract

Cancer signaling encompasses a wide array of entangled molecular cascades that promote oncogenic progression and counteract the effect of tumor suppressors. Transforming growth factor β (TGFβ) induces complex and stage-dependent effects throughout tumor progression. During pre-malignant hyperplastic growth, TGFβ restricts cell proliferation and inflammation, while on the other hand, TGFβ promotes migration and distal metastasis of cancer cells. To dissect the temporal chromatin-based transcriptional response to TGFβ, we employed 3D culture models of isogenic human breast epithelial cells, exemplified by non-oncogenic MCF-10A (MI) and their HRAS-transformed counterpart (MII). Genome-wide chromatin accessibility profiling revealed an extensive chromatin opening induced by TGFβ at transcription start sites and enhancer elements in both models, with a marked enrichment of SOX4 binding motifs in oncogenic cells. Transcriptomic analyses unexpectedly revealed the upregulation of DNA replication and DNA damage response pathways, following TGFβ stimulation of oncogenic MII 3D cultures. Canonical TGFβ-driven programs, including epithelial-mesenchymal transition and metabolic reprogramming, were activated in both models. Notably, single-cell RNA-seq of primary breast tumors confirmed co-expression of *SOX4* and cell cycle regulators. Mechanistically, we show that TGFβ induces the interaction between the MH2 domain of SMAD3 and the intrinsically disordered regions of SOX4, co-activating downstream gene targets. Validating the genome-wide analyses, we found that resistance of breast cancer cells to the CDK4/6 inhibitor palbociclib conferred by TGFβ stimulation was functionally dependent on SOX4. Collectively, our findings reveal an apparent oncogenic function of TGFβ in promoting cell cycle progression and drug resistance through SOX4, highlighting the pro-tumorigenic role of TGFβ signaling in breast cancer progression.

## Introduction

Transforming growth factor-β (TGFβ) suppresses proliferation of most cell types, including cancer cells, while promoting the cell cycle and survival of specific cells when acting coordinately with mitogenic factors or oncogenic signals such as RAS [1, 2]. This bimodal response to TGFβ, established by 2D cell culture studies, explains the anti-/pro-tumorigenic actions of TGFβ in animal models of cancer [3, 4]. Mechanistically, TGFβ induces expression of epithelial or hematopoietic cyclin-dependent kinase (CDK) inhibitors (p15, p21, p57), represses expression of the oncogenic factor c-MYC and arrests their cell cycle [5], enforcing cytostasis [6]. These cell responses are mediated by signaling via type I (TβRI) and type II (TβRII) kinase receptors, phosphorylation of SMAD2 and SMAD3 [7], oligomerization with SMAD4 and accumulation in the nucleus, and SMAD binding to 5’-CAGA-3’ or GC-rich genomic sequences [8]. SMAD interactions with transcription and chromatin cofactors provide physiological cell type- or disease state-dependent gene responses, with alternative signaling effectors, such as protein kinases, contributing coordinately [9, 10].

Breast cancer (BRCA) prevails among women, with metastases accounting for 90% of deaths [11]. BRCA subtypes are based on expression of receptors for estrogen (ER), progesterone (PR) and human epidermal growth factor (HER2), including the aggressive, Basal-like/Triple-negative (TNBC/ER^−^/PR^−^/HER2^−^) tumors [11, 12]. ER^+^/PR^+^-patients receive hormone therapy alone or combined with the CDK4/6 inhibitor palbociclib upon metastasis [13], HER2^+^-patients receive receptor-neutralizing antibodies (e.g., Trastuzumab) [14], and high-grade or receptor-negative patients receive genotoxic chemotherapy alone or combined with immunotherapy [15]. Resistance to endocrine therapy (tamoxifen/aromatase inhibitors) can develop due to acquired ER-mutations or activation of alternative signaling [16]. TNBC patients respond poorly to chemotherapy due to the absence of actionable receptors and drug extrusion induced in part by TGFβ, contributing to poor clinical outcomes [17].

The contribution of TGFβ signaling to the resistance of BRCA and other tumor cells to chemo- or radio-therapy has been established [18, 19]. This is often linked to the induction of epithelial-mesenchymal transition (EMT) by TGFβ, a physiological process occurring during embryogenesis, tissue repair upon wounding and cancer progression [18, 19]. EMT as a component of cancer etiology empowers primary tumors with metastatic potential [20] and enhanced cancer stem cell properties [21, 22], features enforced by TGFβ signaling via the EMT or alternative mechanisms [20].

Diverse molecular mechanisms drive the EMT response to TGFβ [23], and frequently depend on cooperative TGFβ and RAS signaling [24], which mechanistically coordinates transcriptional inputs by the RREB1 protein and epigenetic adaptation on specific gene enhancers [25]. Chromatin modifications on gene enhancers, coupled to the presence of topologically-associated domains of the genome, characterize the EMT response to TGFβ [26]. The link of such chromatin-based processes to resistance of cancer cells to treatment remains unexplored.

BRCA cells exposed to TGFβ over long periods (months to years), sustain their EMT response, while bypassing anti-proliferative and pro-apoptotic responses [27, 28]. Activation of mTOR or Aurora-A kinases and chemokine secretion contribute to pro-survival and chemoresistance responses [27–29]. Interestingly, pancreatic cancer cells responding to palbociclib, activate TGFβ signaling, exhibiting EMT and a fibrogenic matrix-inducing response [30]. Resistance to palbociclib can involve high expression or mutational alteration of CDK6; mutant CDK6 was shown to bypass the anti-proliferative signal of TGFβ, based on a sequestration mechanism of the TGFβ-induced CDK inhibitor p15 [31]. In contrast, resistance to palbociclib caused by high CDK6 expression could be transferable via extracellular vesicle-mediated transport of *miR-432-5p* that downregulates SMAD4, suggesting that TGFβ signaling counteracts resistance in T47D BRCA cells [32]. Similarly, TGFβ3 can cooperate with palbociclib to kill TNBC cells, suggesting that TGFβ3 counteracts resistance to palbociclib generated by high CDK6 expression [33]. Thus, the role of TGFβ in treatment resistance appears also complex.

According to the above open questions, we investigated transcriptional and chromatin architectural modulations in response to TGFβ stimulation in isogenic normal and transformed 3D cultures, revealing the transcription factor SOX4 as uniquely enriched in oncogenic cells. Concomitantly, TGFβ-induced transcriptional rewiring associated with cell cycle processes in oncogenic cells links SOX4 function to palbociclib resistance.

## Materials and methods

### Cell culture, growth factors, and RNA extraction

MCF-10A (abbreviated here as MI) and HRAS-transformed MCF-10AneoT (abbreviated here as MII) mammary epithelial cells were maintained as previously described [34]. The murine BRCA cell line Py2T, engineered to express a red fluorescent protein (RFP) under the epithelial-specific E-cadherin promoter, and human TNBC-basal MDA-MB-231 cells were cultured under 3D and 2D conditions, respectively, as previously described [35]. Human HCC-1937, HCC-1954 and MDA-MB-453 cell lines were cultured in RPMI-1640 medium (Gibco^™^, ThermoFisher Scientific, Sweden). The human HEK293T, human hepatocellular carcinoma HepG2, human glioblastoma U2987MG, parental human lung adenocarcinoma A549, SMAD4 and SMAD2/3/4 triple-knockout A549 cell lines were generated and maintained as previously described [36, 37]. All media (except MI and MII) were supplemented with 10% fetal bovine serum (FBS; Biowest, Almeco A/S, Denmark), 1% penicillin-streptomycin (Gibco^™^, ThermoFisher Scientific, Sweden) and cells were kept in a humidified incubator at 37 °C and 5% CO2. Cells were free of mycoplasma (tested every 2 months) and all cell lines were authenticated using PCR-single-locus-technology (Eurofins, Sweden).

To initiate MI or MII 3D culturing, 2000 cells/100 µl per well were seeded in 96-well Corning^®^ Costar^®^ultra-low attachment plates (Merck, Sweden). After seeding, the cells were centrifuged for 10 s and left to settle down carefully. Individual spheroids started forming about 24 h post-seeding. For TGFβ treatment, 50 µl per well was removed and re-supplemented with fresh medium containing 2 or 2.5 ng/ml of recombinant human TGFβ1 (Peprotech Inc., USA) for 16 h; then, the spheres were collected and centrifuged for 5 min at 1000 × *g*. The pellets were washed twice with cold PBS and then lysed on ice using the ReliaPrep^™^ RNA Cell Miniprep System according to the manufacturer’s instructions (Promega, USA) and RNA samples were stored at −80 °C. The concentration and quality of all extracted RNA samples were assessed by NanoDrop^™^ spectrophotometry (ThermoFisher Scientific, Sweden) and 2100 Bioanalyzer (Agilent, USA). For time-point analysis, 3D spheres were formed, and either stimulated or not with TGFβ1 for 6, 16, 24 or 48 h. Spheres were collected at the respective time-point alongside with untreated spheres at the initial time point (T0) and subjected to RNA extraction.

### Generation of CRISPR/Cas9 knockout cells

The Cas9 protein and a single guide RNA (gRNA; CCGCGCTCCTTCCTGGTCAAGAA) targeting the beginning of exon 1 that encodes the SNAG domain of SNAI2, were delivered to MDA-MB-231 cells as complex (ribonucleoprotein particles) using the Lipofectamine CRISPRMAX Cas9 transfection reagent (CMAX00001, ThermoFisher Scientific, Sweden). Two days post-transfection, living cells were selected and FACS sorted by 7-AAD staining (Thermofisher Scientific, Sweden). The complete procedure was performed at the High Throughput Genome Engineering Facility (HTGE) at Karolinska Institutet, funded by the SciLifeLab https://www.scilifelab.se/units/crispr-functional-genomics/. Then, the cells were moved to our laboratory and single-cell colonies were expanded. Knock-out clones were then validated using ddPCR and immunoblotting.

### Drug treatments and generation of palbociclib-resistant cells

The TβRI inhibitor galunisertib (LY2157299, MedChemExpress, USA) was dissolved in DMSO. Doxorubicin hydrochloride (Sigma-Aldrich, Merck, Sweden) and palbociclib monohydrochloride (TargetMol Chemicals Inc., USA) were dissolved in sterile water. Doxorubicin was introduced to MII-spheres at 500 nM, final concentration, of in the presence or absence of TGFβ stimulation for 48 h. The IC_50_ concentration of each cell line was determined empirically by exposing the cells to an escalated concentration of palbociclib for 72 h. To generate palbociclib-resistant cell lines, cells were exposed to the pre-determined IC_50_ concentration of palbociclib for 72 h followed by drug withdrawal for 48 h and re-exposure to the same concentration of palbociclib for another 72 h. The same routine was repeated until all cells acquired resistance at least to an equal dose to the IC_50_ dose of the drug, and the concentration was escalated gradually following the same procedure. The cells reached full resistance in a period of 4–6 months of continuous drug exposure, and were subsequently cultured in the presence of palbociclib.

### Reverse transcription and real-time quantitative PCR (RT-qPCR)

Total RNA amount (500 ng) per sample was converted into cDNA using the High-Capacity cDNA Reverse Transcription Kit (ThermoFisher Scientific, Sweden) according to the manufacturer’s protocol. RT-qPCR was performed utilizing qPCRBIO SyGreen^®^ Mix (PCR Biosystems, UK) on a Bio-Rad CFX96 thermal cycler (Bio-Rad Laboratories Inc., Germany). Gene expression levels were calculated using the ΔCt method and the complete list of the used primers is provided in Table S7.

### Cell viability, synergy score and colony-forming assays

PrestoBlue HS (high sensitivity) Cell Viability Reagent (ThermoFisher Scientific, Sweden) was used following the manufacturer’s protocol in 96-well plates. 3D cultures were exposed to the cell viability reagent for 4 h, while 2D cultures for 1 h. Fluorescence intensities were detected using EnSpire^®^ Multimode Microplate Reader (PerkinElmer, Inc., USA). To determine the synergy score in drug combination experiments, 5000 cells/well were seeded overnight in a 96-well plate and treated with increasing concentrations of palbociclib or galunisertib alone or in combination for 72 h. Each treatment encompassed eight biological replicates. The synergy scores were calculated based on the cell viability measurement observed in each treatment using the SynergyFinder tool [38]. Each experiment was conducted twice.

For the colony-forming assay, cells were seeded at a low confluency (1000 cells) in six-well plates. Following the attachment and formation of initial colonies, the cells were either stimulated with TGFβ or treated with drugs for an additional ten days. The colonies were fixed with methanol at RT for 20 min and stained with 0.5% crystal violet in 25% methanol. Stained colonies were washed several times with distilled water to remove excessive stain and were left to dry at RT.

### Caspase 3/7 and EdU incorporation assays

The induction of apoptosis, following drug treatments, was measured using the Caspase-Glo^®^ 3/7 Assay kit (Promega, USA) according to the manufacturer’s protocol, in 96-well plates and the spheres were incubated with Caspase-Glo^®^ reagents for 45 min with gentle orbital shaking at RT. Luminescence was detected using an EnSpire^®^ Multimode Microplate Reader (PerkinElmer, Inc., USA). Each treatment condition contained eight biological replicates.

The EdU incorporation assay was performed utilizing the Click-iT™ EdU Proliferation Assay for Microplates (Invitrogen™, ThermoFischer Scientific, Sweden). Spheres generated as described earlier were either simultaneously incubated with TGFβ and 10 µM of EdU or pre-stimulated with TGFβ for 16 h, followed by removal of the medium and incubation with 10 µM of EdU in fresh medium for 16 h. For each experimental condition, 20 spheres were treated independently and then pooled together into five replicates to enhance the detection limit. Subsequently, the spheres were fixed and processed according to the manufacturer’s instructions. Fluorescence was detected at excitation length 568 nm and emission length 585 nm using the EnSpire^®^ Multimode Microplate Reader (PerkinElmer, Inc., USA).

### Transient transfection and generation of stable knockdown cell lines

Predesigned small interfering RNAs (siRNA), either scrambled non-targeting or target-specific against *SOX4*, *SLUG*, *SMAD2*, *SMAD3* and *SMAD4*, were purchased from Sigma-Aldrich, Merck. Transient transfection of MII and MDA-MB-231 cells growing in 2D conditions was carried out using Lipofectamine RNAiMAX Transfection Reagent (ThermoFisher Scientific, Sweden) following the manufacturer’s protocol in 24-well plates with a final concentration of 25 nM for each siRNA. For RNA extraction and immunoblotting experiments, 2D adherent cells were collected after 48 h of transfection. For 3D spheroid experiments, transfected cells were collected 24 h post-transfection, counted and re-seeded in ultra-low attachment plates to generate spheres for an additional 72 h. For transient plasmid transfection, 1 µg of each plasmid per well in a 6-well plate was transfected with Lipofectamine 3000 Transfection Reagent (ThermoFisher Scientific, Sweden) following the manufacturer’s recommendation.

To generate stable knockdown cells, MISSION^®^ pLKO.1-puro (Sigma-Aldrich, Merck, Germany) lentiviral constructs encoding short hairpin RNA (shRNA), either scrambled or *SOX4*-specific, were kindly gifted by Prof. Paul J. Coffer (Utrecht University, The Netherlands). To generate lentiviral particles, 2 µg of each shRNA vector was co-transfected with 1 µg of pCMV-VSV-G-Rev and 1 µg of pCAG-HIVgp vectors, using Lipofectamine 3000 Transfection Reagent (ThermoFisher Scientific, Sweden), into HEK293T cells. The cells were incubated for 4 h in DMEM medium, then the medium was replaced with DMEM/F-12 medium supplemented with 5% horse serum and the cells were incubated for an additional 48 h. Subsequently, medium containing released lentiviral particles was collected and spun down at 1000 × *g* for 5 min and the supernatant, corresponding to each shRNA vector, was added to MII-cells seeded in 6-well plates for 24 h, then 1 µg/ml of puromycin (Sigma-Aldrich, Merck, Sweden) was added to select stably transduced MII-cells for 48 h. Following that, fresh medium was added to each well containing 0.5 µg/ml of puromycin to maintain the MII-stable clones. The knockdown efficiency was assessed using qRT-PCR.

### SOX4 and reporter cloning and generation of stable cell clones expressing CAGA-GFP and CAGA-Luc

Full-length *SOX4* and its two shorter isoforms were amplified by PCR using cDNA from U2987MG cells. The PCR products were inserted into pcDNA3 vector using *Eco*R I and *Xba* I sites. Expression plasmids for SOX4 lacking its DNA-binding domain (ΔDBD) and truncated versions of SOX4 were generated by PCR using appropriate primers and the full-length SOX4 expression plasmid as a template. The oligonucleotide that encodes TWIN-FLAG tag (WSHPQFEKGGGSGGGSGGSAWSHPQFEKDIDYKDDDDKG) was inserted into an expression vector driven by the *EF-1α* promoter using *Bam*H I and *Eco*R I sites.

All the reporter vectors used for transient transfection in this study have the pGL4-MLP backbone. The oligonucleotides were inserted using *Kpn* I and *Xho* I sites. The lentiviral vectors encoding green fluorescence protein (GFP) or luciferase under the control of CAGA_12_-MLP (CAGA-GFP or CAGA-Luc) were generated by Gateway technology (ThermoFisher Scientific, USA).

To generate MII-expressing stable CAGA-GFP or CAGA-Luc reporters, the lentiviral vectors were introduced into HEK293T cells with pCMV-VSV-G-RSV-Rev and pCAG-HIVgp to produce lentiviral particles as described above. The MII-cells were then transduced with the particles and selected using puromycin for 48 h.

### CAGA-Luciferase reporter assay

For 2D cultures, cells were transiently co-transfected with 150 ng of the TGFβ/SMAD-responsive (CAGA)_12_-Luc construct and 12 ng of pRL-TK vector, which encodes the *Renilla* luciferase to normalize the firefly luciferase measurements. Luciferase assays were performed using the Firefly and Renilla Dual Luciferase Assay kit (Biotium, USA) following the manufacturer’s instructions. Relative normalized luciferase detected values were derived from independent biological triplicates. For 3D spheroids, MII-cells stably expressing the CAGA-Luc reporter were seeded and transiently transfected with the siRNAs for 24 h. Then, the transfected cells were collected, and an equal number of cells were re-seeded in ultra-low attachment plates to create spheroids for 48 h, followed by TGFβ stimulation for 6 h. Ten independent spheres were pooled together to represent one biological replicate to obtain a reproducible signal. The experiment was performed with three biological replicates, each with two technical replicates. The luminescence intensities were recorded without normalization with a second reporter.

### Zebrafish engraftment and optimization

Zebrafish (*Danio rerio*) experiments were conducted at the Genome Engineering Zebrafish National Facility (currently DanioReadout, Uppsala University). Zebrafish adults and embryos from the AB line were maintained as previously described [39]. At two days post fertilization (2 dpf) at least 300 embryos were injected with palbociclib-resistant BRCA cell lines (MDA-MB-231 and HCC-1937). No randomization method was applied and the microinjector was blinded to the groups of injected cancer cells. Prior to the injection, the cells were maintained at the exponential growth phase. The medium was aspirated and the cells were washed twice with warm PBS, stained with Vybrant™ Multicolor Cell-Labeling Kit (ThermoFisher Scientific, Sweden) at 1:100 v/v in PBS and incubated for 30 min at 37 °C in a humified incubator with 5% CO_2_, followed by incubation for 5 min at 4 °C. The staining solution was aspirated and washed twice with warm PBS, and cells were collected with trypsin. Then, the cells were spun down and washed twice with PBS, and finally, they were re-suspended in 1 ml of PBS and stored on ice after measuring their viability. Shortly, 500 cells per embryo were injected either into the bloodstream or in the perivitelline space (PVS), kept at 33 °C and monitored alive at 24 h time intervals. When embryos were injected into the bloodstream, they showed high mortality rates attributed to cardiac edema (see “Results”). On the other hand, PVS injection led to the formation of loose, local tumors with micrometastases at 24 h post-injection, which surprisingly attracted melanocytes and caused a 70% mortality rate at 48 h (see “Results”). Optimized engraftment by injecting resistant HCC-1937 cells into the yolk-sack counteracted this limitation, and fluorescently-labeled cells formed tumor masses without adverse effects (see “Results”). The maximum tolerated dose of palbociclib and galunisertib in non-tumor-bearing zebrafish larvae was measured, followed by a morphological (phenometric) analysis. To identify and quantify distinct morphological regions, phenometrics implemented a deep learning-based segmentation pipeline. The model, built on a ResNet-50 backbone, was trained using a dataset of 2250 annotated images. Following segmentation, four features were quantified: body area, eye area, pericardiac area, and the presence of an inflated swim bladder. Then, resistant HCC-1937 cells were engrafted into the larval yolk-sac 2 dpf, and fluorescently-labeled tumors were imaged and measured at 24 h post-engraftment (3 dpf). Subsequently, the larvae were exposed to palbociclib (20 µM), galunisertib (20 µM), or their combination for 48 h (5 dpf), followed by quantifiable imaging to determine tumor growth rates.

### Western blotting and immunoprecipitation (IP)/pull-down assays

Total cellular proteins were extracted using RIPA lysis buffer (0.1% SDS, 0.5% sodium deoxycholate, 50 mM Tris-HCl pH 7.5, 150 mM NaCl, 5 mM EDTA and protease inhibitor cocktail) by continuous vortexing for 15 min at cold, followed by brief sonication for 2 min and centrifugation at max speed for 15 min at 4 °C. The supernatants were carefully transferred to new tubes and protein concentrations were determined using Pierce^™^ BCA Protein Assay Kit (ThermoFisher Scientific, Sweden). For each sample, 40 µg of protein was boiled for 10 min in NuPAGE™ LDS Sample Buffer (ThermoFisher Scientific, Sweden) supplemented with β-mercaptoethanol at 2.5% final concentration. Upon gel electrophoresis and transfer to nitrocellulose membranes using a wet transfer unit (Bio-Rad Laboratories Inc., Sweden), Western blotting was performed as described [40].

For each IP condition, 1.5 × 10^6^ cells were seeded in 100 mm culture dishes and incubated overnight. Following transfection with the respective plasmid, the cells were incubated for 24 h and were subsequently either stimulated for an additional 24 h or left unstimulated. The cells were lysed in 3 ml of cold RIPA lysis buffer (0.5% Triton X-100, 0.5% sodium deoxycholate, 20 mM Tris pH 7.5, 150 mM NaCl and 10 mM EDTA supplemented with 1× protease and phosphatase inhibitors) by vigorous vortexing for 5 min. Then, the cells were sonicated for five cycles (30 s ON, 30 s OFF at high pulse) and centrifuged at max speed for 10 min at 4 °C. The supernatants were carefully transferred to new 1.5 ml tubes (three tubes per condition) and 1% of the total volume of each condition was kept as an input. To proceed with the IP of FLAG-tagged proteins, we utilized Pierce™ Anti-DYKDDDDK Magnetic Agarose Beads (ThermoFisher Scientific, Sweden), resuspended and washed three times in RIPA lysis buffer on ice. Washed beads (25 µl) were added to each 1 ml of the cell lysates and incubated overnight at 4 °C with gentle rotation. Upon magnetic precipitation on ice, the beads containing the immunocomplex were washed three times with RIPA lysis buffer and finally resuspended in 25 µl of sample buffer adjusted as 2× buffer and supplemented with β-mercaptoethanol, boiled for 10 min and immediately separated on a magnetic rack, whereas the input samples were boiled for 5 min in the same volume of sample buffer.

For each pull-down assay, 2 × 10^5^ cells were seeded in 6-well plates and incubated overnight. Following transfection with the respective plasmid, the cells were incubated for 24 h, lysed in 300 µl of a buffer containing 1% Nonidet P-40, 20 mM Tris-HCl (pH 7.4), 150 mM NaCl, 5 mM EDTA, protease inhibitor mixture (Nacalai Tesque, Japan), and phosphatase inhibitor mixture (Nacalai Tesque, Japan), centrifuged at 15,000 rpm for 5 min at 4 °C, with supernatants carefully transferred to new 1.5 ml tubes (three tubes per condition) and 3% of the total volume of each condition kept as input. For each 280 µl of cell lysate, 10 µl Strep-Tactin^®^ Sepharose^®^ resin (IBA Lifesciences GmbK, Germany) were added and the lysates were incubated for 1 h at 4 °C with gentle rotation, centrifuged and the beads containing the protein complex were washed three times with wash buffer containing 1% Nonidet P-40 20 mM Tris-HCl (pH 7.4) and 150 mM NaCl, and finally resuspended in 20 µl of sample buffer adjusted as 2× buffer and supplemented with β-mercaptoethanol. The pull-down and input samples were boiled for 5 min, resolved by SDS-PAGE and detected by Western blotting. A list of all primary antibodies is given in Table S7 and the original immunoblots are presented at the end of the Supplementary file.

### Gene expression analysis of MI- and MII-spheres using nanoString profiling

For each time point, independent biological triplicates of MI- and MII-spheres were subjected to RNA extraction as described above, and 100 ng of total RNA/sample was loaded into the nCounter^®^ Tumor Signaling 360^TM^ panel (nanoString, USA) and hybridized overnight following the manufacturer’s protocol. Data acquisition was performed on the nanoString nCounter^®^ FLEX Analysis System at Clinical Genomics Uppsala, SciLifeLab. Raw data were analyzed using nSolver^TM^ Analysis Software v4.0 (nanoString, USA), where the imaging QC threshold was determined at 75% field of view and positive control linearity cutoff was set at 0.95; in addition to a positive control limit of detection more than two standard deviation units above the negative control values. Data normalization was performed utilizing a combination of positive control and housekeeping normalization built-in functions of the nSolver^TM^ Analysis Software. The differential expression analysis was done using the nCounter^®^ Advanced Analysis module v2.0.134, implementing the DESeq2 package. Normalized probe intensities are listed in Table S1.

### Bulk RNA and scRNA sequencing data analysis

The library preparation step was performed with 500 ng total RNA/sample using the TruSeq stranded total RNA library preparation kit with RiboZero Gold treatment and unique dual indexes following the manufacturer’s protocol (Protocol # 1000000040499, Illumina Inc., San Diego, CA, USA). Paired-end reads with a length of 150 bp were generated using SP flowcell and v1.5 sequencing chemistry on a NovaSeq 6000 sequencing platform located at the SNP&SEQ facility, National Genomics Infrastructure (NGI), SciLifeLab-Uppsala site. At least 30 million raw reads were generated per sample. The adapters were trimmed using the ILLUMINACLIP function of the Trimmomatic tool, where low-quality reads were also removed. The quality of trimmed reads was checked using the FastQC tool. We performed read alignment to the reference genome (GRCh38 genome assembly) using STAR Aligner with a two-pass alignment mode [41]. The features of the aligned reads were quantified against the comprehensive gene annotation from Gencode (GRCh38.p13) using featureCounts of the Subread package [42]. The quantified reads were normalized using the CPM method embedded in the DESeq Bioconductor package in R [43] and the differential expression analysis was done using standard input and *p*-value correction at a threshold of 0.05 for false discovery rate (FDR). Gene set enrichment analysis (GSEA) was carried out with the GSEA tool and the molecular signature database MSigDBv6 [44]. For scRNA-seq analysis, the expression count matrix and metadata were obtained from the publicly available GEO repository (accession number GSE176078). Seurat package v5.2.1 in R [45] was used to create an object for downstream data analysis. Stringent filtering criteria were applied, including a minimum of 8000 reads and 200 uniquely identified features per cell, with a percentage of mitochondrial genes less than 10% of the total features. Doublets were removed using the DoubletFinder package v2.0.3 [46]. Data normalization was performed using the SCTransform function in Seurat, followed by dimensionality reduction and clustering with default parameters. Data visualization was performed in RStudio v2024.12.0 Build 467 running R environment v4.4.2. All the lists of DEGs, PAM50 analysis and pathway enrichment analysis data are presented in Tables S3–S6.

### Chromatin tagmentation, ATAC-seq, intersection, and motif enrichment analyses

Following TGFβ treatment for 16 h, spheres were collected, dissociated in trypsin for 5 min at 37 °C with agitation, and then resuspended and counted using an automated cell counter (ThermoFisher Scientific, Sweden). Spheroid-derived single-cell suspensions of 75,000 living cells per condition were transferred to cold 1.5 ml tubes, washed once with 50 µl of cold PBS and centrifuged for 5 min at 500 × *g* in the cold. The cell pellets were then lysed in 50 µl of ice-cold lysis buffer (10 mM Tris-HCl, pH 7.4, 10 mM NaCl, 3 mM MgCl_2_, 0.1% NP-40) and immediately centrifuged for 5 min at 500 × *g* in the cold. We performed chromatin tagmentation using 2.5 µl/reaction of Nextera Tn5 transposase (Illumina Inc., USA) and subsequent chromatin purification as well as PCR amplification as previously described [47]. The generation of 50 bp paired-end reads was performed using the SP flowcell on a NovaSeq 6000 platform located at the NGI facility at SciLifeLab, Karolinska Institute, Stockholm. The ATAC-seq peak-calling, QC and differential accessibility were carried out utilizing the standard Nextflow pipeline of nf-core/atacseq v1.2.1 with default parameters. The statistics of differential accessibility were obtained using the DESeq2 tool in R. The intersection analysis was done using BEDTools v2.31.1 [48], where a 50% feature overlap was determined as a cutoff for the intersection between significantly enriched peaks in BED format corresponding to different experimental conditions. Motif enrichment analysis and feature annotations were performed utilizing the HOMER suite v4.11 [49], where several iterations were carried out and adjusted *p*-values were used. The BED files corresponding to differentially enriched ATAC-seq peaks, intersection analysis, annotated human TSS and enhancers are listed in Table S2.

### CUT&RUN and chromatin immunoprecipitation (ChIP) assays

The CUT&RUN (cleavage under targets and release using nuclease) Assay Kit (Cell Signaling Technology, USA) was utilized against MII-spheres, where multiple spheres (3 × 10^5^ cells/condition) were collected and dissociated into a single-cell suspension through rigorous pipetting and treatment with trypsin with continuous shaking for 5 min at 37 °C. The single cells were cross-linked with 2.7 µl of 37% formaldehyde per 1 ml of cell suspension for 2 min at room temperature (RT). The cross-linking was quenched using glycine and the suspension was incubated for 5 min at RT with gentle rotation. The cell suspension was immediately centrifuged for 3 min at 3000 × *g* at 4 °C, the supernatant was removed and each pellet was resuspended in 1 ml of the 1× wash buffer. The cell immobilization and subsequent permeabilization, immunoprecipitation and pAG-MNase-based DNA fragmentation were performed according to the manufacturer’s instructions. The IgG antibody served as a negative control for the immunoprecipitation reaction. Specific primers were designed to span extended regions upstream of the transcription start sites of target genes.

For each ChIP assay, 5 × 10^6^ cells/condition were utilized. Cells were washed twice with PBS and fixed on the plate with 10 ml of 1.1% w/v formaldehyde diluted in PBS for 10 min at RT with gentle shaking. The reaction was quenched with 0.125 M glycine for 5 min. The fixed cells were washed twice with 10 ml of cold PBS and scraped from the culture plate on ice using 1 ml of cold PBS. Immediately, the cells were centrifuged at 4000 × *g* for 10 min at 4 °C, and the fixed pellets were lysed in 1 ml of lysis buffer (0.1% SDS, 0.5% Triton X-100, 20 mM Tris-HCl, pH 8, and 150 mM NaCl, 1 mM phenylmethylsulfonyl fluoride (PMSF)) supplemented with protease inhibitor cocktail (Sigma-Aldrich, Merck, Sweden) and incubated on ice for 30 min with continuous pipetting. Each lysed pellet was transferred to 10 ml Bioruptor^®^ Plus tubes (Diagenode SA, Belgium) and subjected to chromatin shearing using a Bioruptor^®^ (Diagenode SA, Belgium) for five cycles (30 s ON, 30 s OFF at high pulse). The insoluble components were removed by centrifugation at maximum speed for 10 min at 4 °C. For each reaction, we used 60 μl of Sheep-Anti Mouse IgG Dynabeads^™^ (ThermoFisher Scientific, Sweden). The beads were washed twice with PBS and then incubated on rotation at 4 °C for 6 h with 4 μg of either H3K4me3 antibody (Abcam, UK) or IgG antibody as a negative control. The respective antibodies were diluted in 1 ml of IgG-free 0.5% BSA in PBS (Merck KGaA, Germany). The conjugated beads were washed twice with cold PBS and then incubated with 1 ml of clear lysate overnight at 4 °C with gentle rotation. The bound immune complexes were separated using magnetic precipitation followed by two washes with 1 ml low salt buffer (0.1% SDS, 1% Triton-X 100, 2 mM EDTA, 20 mM Tris-HCl, pH 8, 150 mM NaCl, 0.5 mM PMSF and protease inhibitors) for 7 min at 4 °C with gentle rotation. The immune complexes were then washed once with 750 µl of the same buffer containing high salt, 0.5 M NaCl, for 7 min at 4 °C with gentle rotation. A final PBS wash was done, followed by the addition of 200 μl of elution buffer (50 mM Tris-HCl, pH 8, 1% SDS, 10 mM EDTA and 0.5 mM PMSF) and samples were incubated at 65 °C for 30 min with rigorous agitation, followed by magnetic separation. The eluted material was de-crosslinked overnight at 65 °C. DNA isolation was performed using QIAquick PCR purification kit (Qiagen AB, Sweden). Quantitative PCR was performed and the relative fold-enrichment over IgG for each condition was calculated. A list of primers is given in Table S7.

### Statistical analysis

The results express mean values of at least three independent biological repeats as explained in the methods and figures. The number of replicates is indicated in every figure legend. After determining the efficiency of each cell-based assay, the number of technical and biological repeats was defined, and the appropriate statistical method was selected based on sample content and variation within each dataset included for comparison. Error bars represent standard error of the mean SEM and occasionally SD. The variance was similar between the groups that have been compared. Accordingly, the statistical method reported in the figure legends was chosen, and additional statistical methods are included in the methods. Statistical significance is represented by p-values *p ≤ 0.05, **p ≤ 0.01, ***p ≤ 0.001.

## Results

### Hypoxic and EMT gene expression profiles distinguish 3D growth of oncogenic breast epithelial cells

**Fig. 1:**
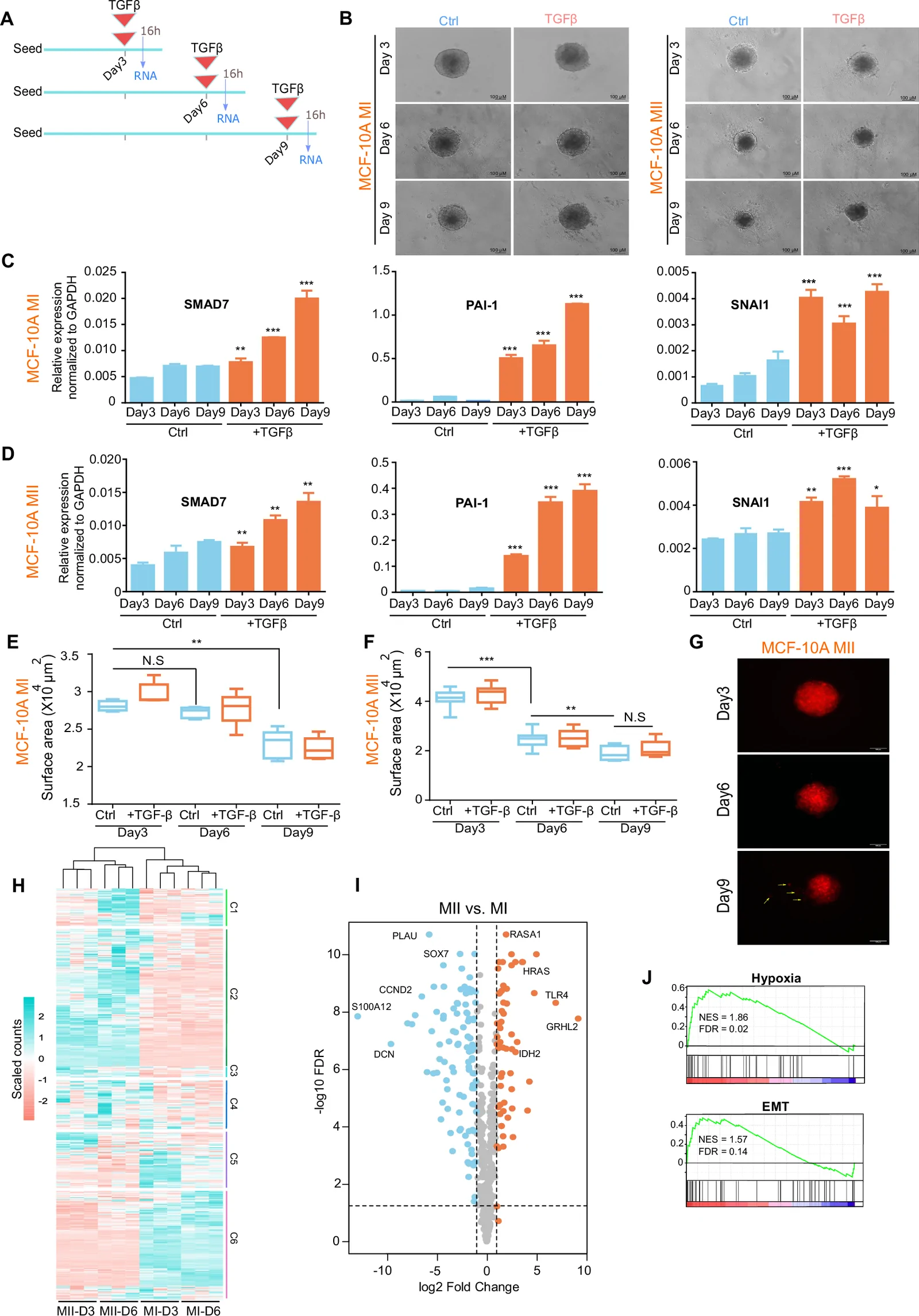
MI- and MII-cell growth in 3D highlights dynamic propagating activity. **A** Schematic illustration of different growth conditions examined for establishing MI- and MII-derived spheres seeded in ultra-low attachment 96-well plates prior to TGFβ treatment. **B** Phase-contrast micrographs of MI- and MII-spheres growing at different time intervals with or without TGFβ treatment. (Scale bar, 100 µm). RT-qPCR analysis of TGFβ-responsive genes in MI- (**C**) and MII- (**D**) spheres growing at the indicated time intervals upon TGFβ stimulation. Values represent mRNA expression levels normalized to *GAPDH*. Data are presented as mean values of three biological replicates ± SEM. Boxplots with median values and whiskers representing minimum and maximum values illustrating the quantification of surface area (µm^2^) of more than 20 independent MI- (**E**) or MII- (**F**) spheres per condition. **G** Representative fluorescence imaging of MII-spheres stained with a lipophilic tracer at the indicated time intervals. The arrows point to the cells migrating away from the structured sphere. (Scale bar, 100 µm). **H** Heatmap demonstrating the expression of 780 genes in MI- and MII-spheres collected on Day3 (D3) and Day6 (D6) post-seeding and measured by nanoString nCounter^®^ Tumor Signaling 360^TM^ panel. The k-means clustering algorithm identified six distinct clusters (C1 – 6) of expressed genes. The color-coded scale represents normalized probe intensities corresponding to gene expression levels. **I** Volcano plot showing the DEA of the investigated transcripts in MII-spheres, combining D3 and D6 samples against all MI-samples as reference. The vertical dashed lines demarcate log2 fold-change values of ±1 and the horizontal dashed line corresponds to an FDR value < 0.05. **J** GSEA plots showing the upregulation of hypoxia and EMT hallmarks, indicated by the normalized enrichment scores (NES) in Day6 MII-spheres compared to Day3 spheres. Statistical significance in **C–F** was derived using a two-tailed unpaired Student’s *t* test. *p*-values *p ≤ 0.05, **p ≤ 0.01, ***p ≤ 0.001. N.S., not significant.

Mammospheres formed 24 h post-seeding of normal-like breast MCF-10A (MI) cells with mammary morphogenetic potential that form 3D acini [50], or MCF-10AneoT (MII) cells harboring oncogenic HRAS and capable of producing carcinomas in xenografts [51], were stimulated with TGFβ1 (hereafter abbreviated as TGFβ) for 16 h at three, six, or nine days post-seeding (Fig. 1A, B). As cultures aged, upregulation of canonical TGFβ-responsive genes was enhanced (*SMAD7*, *PAI-1*) or stabilized (*SNAIL*/*SNAI1*) (Fig. 1C, D), whereas the sphere surface area was significantly reduced (MI/Day9, MII/Day6 after seeding) (Fig. 1E, F). Fluorescent tracking confirmed MII-surface area decrease on Day6, followed by individual cell migration on Day9 (Fig. 1G), that was also reproduced in murine Py2T BRCA cells that express RFP under the *E-cadherin* promoter [35] (Day7; Fig. S1A). Oncogenic cultures dissociated spontaneously after Day40, creating daughter colonies, able to propagate after re-seeding (Fig. S1B).

Hierarchical clustering after targeted NanoString profiling across Day3 and Day6 spheres separated MI- from MII-cultures and aligned with sphere age, while k-means clustering revealed the time-dependent patterns (Fig. 1H). Clusters C2-C3-C4 and C5-C6 and most notable C2 and C6 showed reciprocally inverse expression patterns between MI- and MII-cells (Fig. 1H). Differential expression analysis (DEA) of all MII- against all MI-spheres confirmed HRAS overactivation in MII, along with epithelial/EMT-related factors (Fig. 1I; *EPCAM*, *GRHL2*). MII-spheres expressed more differentially expressed genes (DEGs) than MI-spheres between Day3 and Day6 (Fig. S1C – F, Table S1). Gene set enrichment analysis (GSEA) revealed hypoxia and EMT hallmark enrichment in Day6 versus Day3 MII-spheres (Fig. 1J). Subsequently, Day3 cultures with 16 h TGFβ stimulation were analyzed to capture early transcriptional and chromatin modulations accumulating before a full replication cycle.

### TGFβ stimulation exposes distinct chromatin regions in normal versus oncogenic spheres

**Fig. 2:**
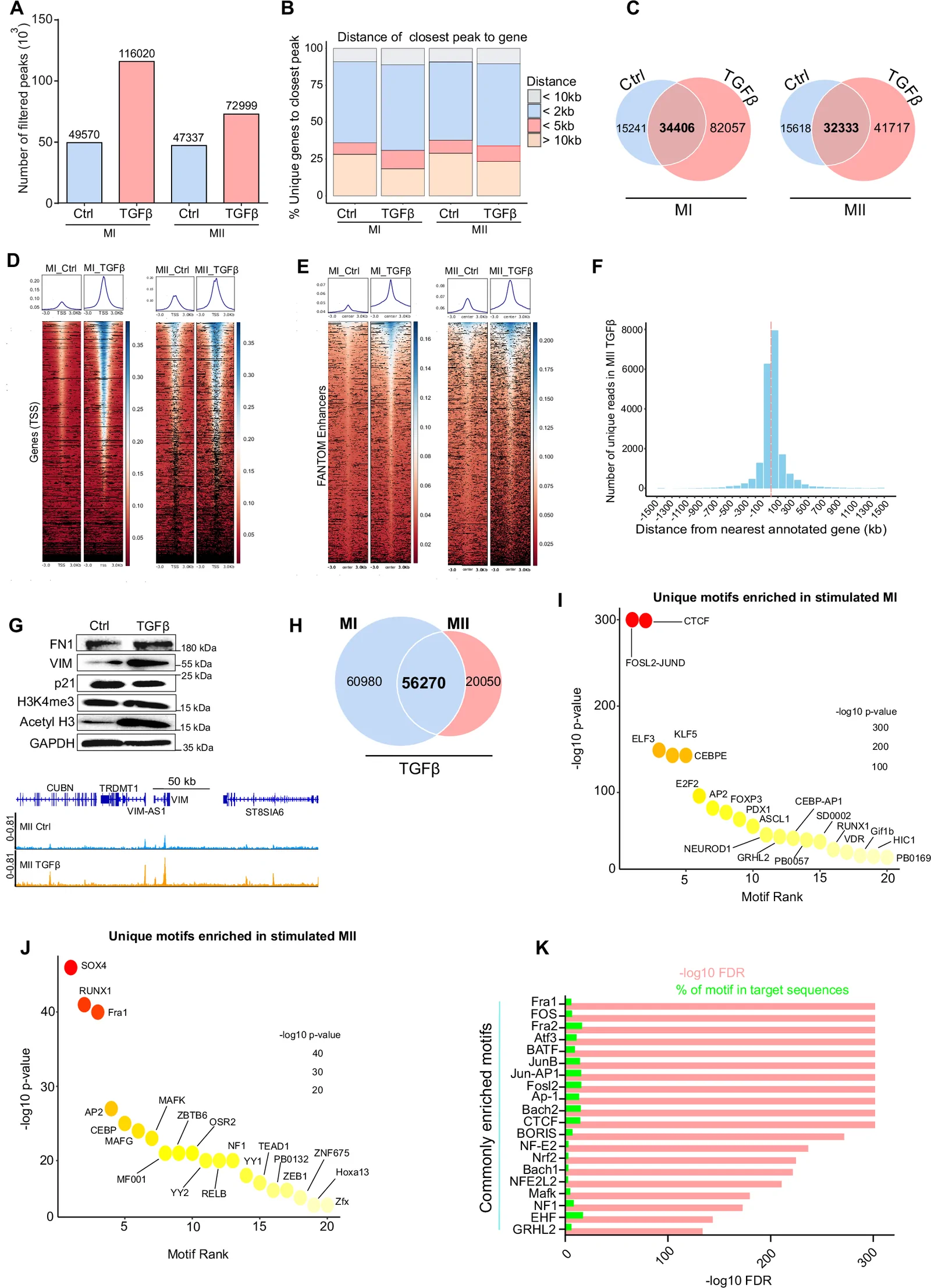
TGFβ enhances chromatin accessibility in MI- and MII-spheres and selects SOX4 in oncogenic MII-spheres. **A** Bar graph showing the total number of significantly enriched peaks in MI- and MII-spheres following TGFβ stimulation. **B** Distribution of genomic distances of the peaks enriched in different samples relative to the annotated unique genes. (**C**) Intersection analysis demonstrating the number of significantly enriched common and unique peaks in response to TGFβ treatment in MI- (left panel) and MII-spheres (right panel). Cumulative heatmaps spanning a window of ±3 kb distance of the enriched peaks in MI- and MII-spheres centered around the annotated genome-wide TSS (**D**) and annotated enhancers (**E**) derived from the FANTOM5 catalog. The color-coded scales represent the relative peak enrichment values for individual comparisons and the top diagrams represent the same values for the highest enriched gene. (**F**) Distance distribution, measured in kb, of uniquely enriched peaks in MII-spheres relative to the nearest annotated gene. The vertical dashed line indicates the start position of the gene. **G** Immunoblotting of the indicated proteins (FN1: Fibronectin, VIM: Vimentin, Acetyl H3: Acetylated histone 3) in MII-spheres following TGFβ stimulation (upper panel) and a genome browser snapshot featuring the enriched ATAC-seq peaks in unstimulated (Ctrl) and stimulated MII-spheres. The values on the Y-axis represent the normalized scale for both conditions. **H** Intersection analysis demonstrating the number of common and unique peaks in TGFβ-stimulated MI- and MII-spheres. Motif enrichment analysis of uniquely enriched peaks in MI- (**I**) and MII- (**J**) spheres treated with TGFβ. The X-axis represents the motif rank, and the Y-axis indicates the levels of statistical significance expressed on a log scale. **K** Motif analysis of peaks commonly enriched in stimulated MI- and MII-spheres. The X-axis represents the levels of significance (pink color) and the percentage of each identified motif in the target peaks (green color), while the Y-axis indicates the enriched TF motifs.

Chromatin remodeling in dividing cells supports tissue lineage specification [52], including mammary organoid development [53]. We optimized ATAC-seq assays [47] in 3D cultures where principal component analysis (PCA) showed reproducible, distinct chromatin profiles by cell type (PCA1) and TGFβ treatment (PCA2) (Fig. S2A). TGFβ increased chromatin accessibility in both MI- (>2-fold) and MII-spheres (Fig. 2A), and with peaks mapped mainly in intronic and intergenic regions (Fig. S2B) where distal peaks (>10 kb) declined and proximal peaks (≤2 kb) increased (Fig. 2B, C). In MI-spheres, TGFβ upregulated and downregulated the accessibility of 13,960 and 4214 peaks, respectively (Fig. S2C, Table S2), whereas 1718 and 833 peaks were upregulated and downregulated, respectively, in MII-spheres (Fig. S2D, Table S2). Moreover, TGFβ treatment upregulated 6275 and downregulated 10,066 peaks in MI- compared to MII-spheres (Table S2), implying that epigenetic landscape differences are primarily driven by genotype.

Enrichment matrix analysis showed that TGFβ stimulation elevated chromatin accessibility around (±3 kb) annotated transcription start sites (TSS) and at selected enhancers (annotated across cell and tissue types by FANTOM5 [54]) (Fig. 2D, E). Most of the 41,717 enriched peaks in stimulated MII-spheres (Fig. 2C) localized within ±100 kb of the closest gene (Fig. 2F), consistent with vertebrate enhancer positioning [55]. Immunoblotting in MII-spheres confirmed elevated active enhancer (acetyl-H3) and promoter (H3K4me3) marks after TGFβ stimulation (Fig. 2G), and increased vimentin levels, a known TGFβ target, and a slight decrease in p21, the latter being compatible with the observed onset of spheroid size decrease (Fig. 1B).

To explore transcriptional differences between MI- and MII-spheres after TGFβ stimulation, consensus peak analysis (Fig. 2H) and multiple iterations of consensus transcription factor binding site identification indicated, among others, FOSL2-JUND, CTCF and ELF3 motifs in MI-unique peaks (Fig. 2I), and SOX4, RUNX1 and FRA1 motifs in MII-unique peaks (Fig. 2J). SOX4 stood out due to its known role in oncogenic TGFβ signaling [56], with its binding sites (Fig. 2J) mostly found in intronic/intergenic regions and near promoter-TSS zones linked to diverse biological functions (Fig. S2E, Table S2). SOX4 motifs peaked around ±2 kb relative to the enriched TSSs (Fig. S2F, G), and were not shared between stimulated MI- and MII-spheres (Fig. 2K), indicating TGFβ specificity in oncogenic cells. Lastly, pathway enrichment analysis of common promoter-TSS regions in stimulated MI- and MII-spheres revealed genes regulated by Myc, mTORC1, hypoxia and TGFβ signaling, oxidative phosphorylation genes, and genes linked to regulation of the G2/M cell cycle phase (Fig. S2H, I). Thus, TGFβ stimulation increases chromatin accessibility with distinct transcription factor specificities in HRAS-transformed mammary spheres.

### TGFβ induces cell cycle and DNA replication gene programs in oncogenic spheres

**Fig. 3:**
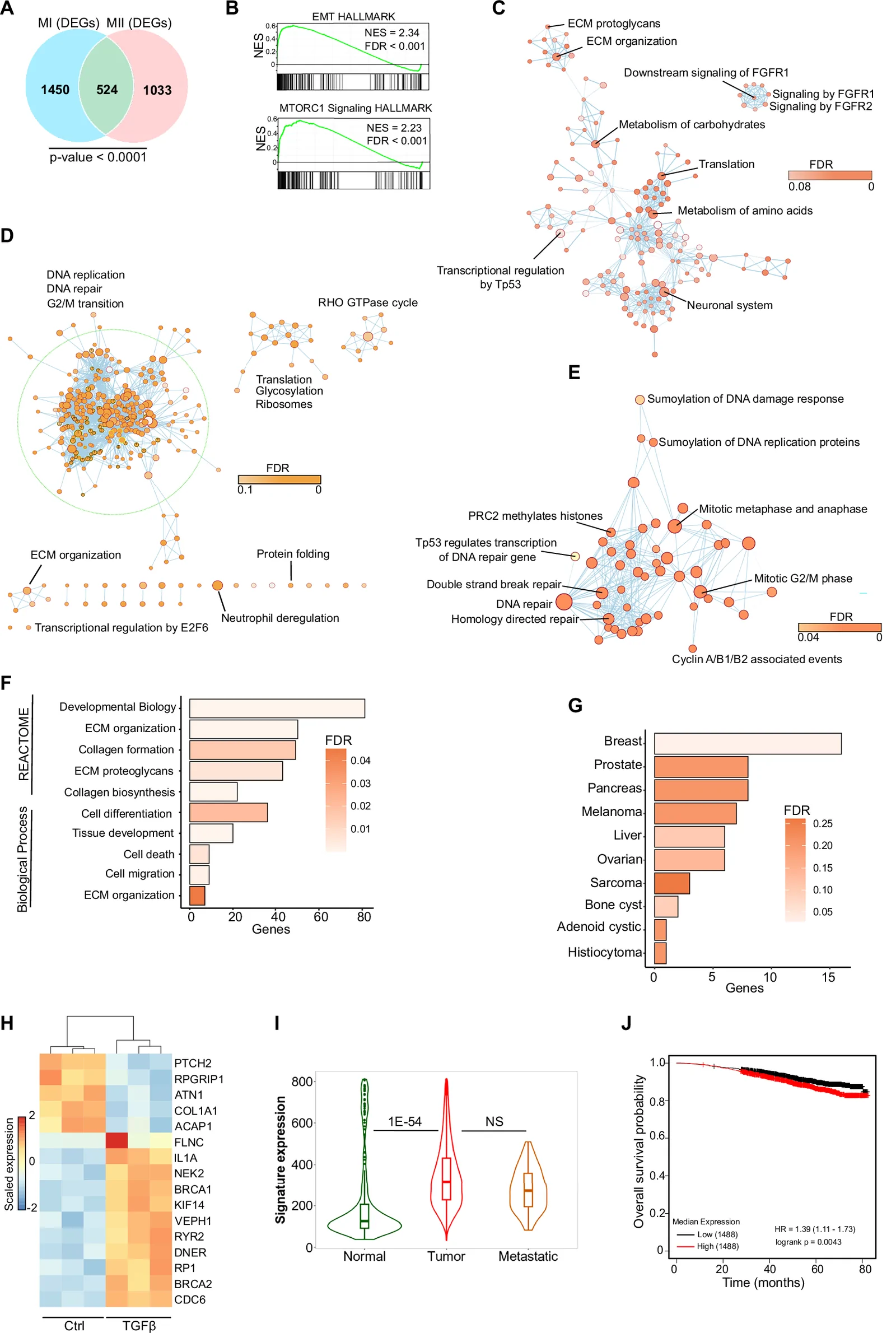
The transcriptional response of oncogenic MII-spheres to TGFβ stimulation generates a BRCA-specific gene signature. **A** Intersection analysis indicating the number of DEGs (log2 fold-change > ±1 and FDR < 0.05) in MI- and MII-spheres following TGFβ stimulation. **B** GSEA plots showing the positive enrichment of EMT and mTORC1 signaling hallmarks, indicated by the NES in TGFβ-stimulated MI-spheres versus the respective unstimulated spheres. Network interaction visualization of significantly enriched (nominal *p*-value < 0.005, FDR < 0.1 and overlap-coefficient >0.5) REACTOME terms in stimulated MI- (**C**) and MII- (**D**) spheres compared to unstimulated spheres, with FDR values color-coded. **E** Zoomed view of interacting pathways (from **D**) involved in DNA repair and cell cycle-related processes in stimulated MII-spheres. **F** Bar graph showing the top significant biological processes and REACTOME pathways enriched in the MI and MII commonly dysregulated genes. The enriched terms are shown on the Y-axis, and the corresponding number of genes involved in each term is shown on the X-axis, which is color-coded with FDR. **G** Bar graph demonstrating different tumor types (Y-axis) identified by the NCG repository, based on the expression patterns of unique genes (X-axis) differentially expressed (FDR color-coded) in stimulated MII-spheres. BRCA represents the top significantly identified tumor, harboring the highest number of dysregulated genes. **H** Heatmap exhibiting the 16 gene signature expression patterns that significantly identify BRCA. The color-coded scale represents the scaled expression in stimulated and unstimulated (Ctrl) MII-spheres. **I** Violin plots showing the collective expression of the 16 genes in normal, primary tumors and metastatic breast tissues. Statistical significance was derived using an ANOVA test. **J** Kaplan-Meier survival plot demonstrating the prognostic value of the combined expression signature of the 16 genes in BRCA patients with the associated hazard ratio (HR).

RNA-seq analysis of stimulated spheres showed 1974 MI and 1557 MII DEGs, representing protein-coding (MI 60.4%, MII 51.5%) and long noncoding RNAs (lncRNAs, MI 28.1%, MII 33.98%) (Fig. 3A, Fig. S3A – G, Table S3, S4), classified in hallmark pathways of EMT (MI/MII), oxidative phosphorylation (MI/MII), cholesterol homeostasis and mTORC1 signaling (MI), transcription factor E2F targets, genes acting at the G2/M checkpoint and the mitotic spindle in MII (Fig. 3B, Fig. S3A, E). Construction of a network of interacting pathways using REACTOME, highlighted ECM remodeling, FGF/FGFR signaling, citric acid cycle and electron transport system, amino acid metabolism and protein translation in MI and RHO GTPases, ribosome biogenesis and protein translation/glycosylation, and cell cycle-related processes in MII (Fig. 3C, D, Table S3). The latter harbored many interacting nodes, including G1 and G1/S phase transition, DNA replication, G2/M phase transition, DNA repair and histone modifications (Fig. 3E), with over 200 genes tied to DNA replication processes (Fig. S3H). TGFβ stimulation produced a molecular signature overlapping with known oncogenic profiles (Fig. S3D, Table S3), including TANK-binding kinase 1 (TBK1), an innate immune response modulator in intestinal epithelial cells [57] and mediator of platelet-induced EMT [58]; serum-induced response of fibroblasts [59]; rapamycin response of leukemic cells [60]; MYC overexpression in BRCA cells [61]. These signatures suggest an oncogenic and metabolic shift in MI-spheres following TGFβ stimulation. In MII, the oncogenic signature genes of *EGFR*, *KRAS*, *MEK*, *ERBB2*, *E2F1*, *VEGF* and the chromatin modulator *PRC2* shared similar positive gene expression patterns with TGFβ stimulation (Table S4). Thus, based on whole transcriptome analysis, TGFβ enhances DNA replication and exerts oncogenic functions in HRAS-transformed spheres, similar to known tumor-promoting factors.

### Relevance of the TGFβ-regulated gene signature to BRCA classification

Focusing on shared MI/MII responses to TGFβ by analyzing the 524 common DEGs with similar expression patterns (Fig. 3A), except for 15 transcripts (Fig. S3I), we identified ECM remodeling, cell migration and differentiation functions (Fig. 3F). In contrast, the 1033 MII-specific DEGs (Fig. 3A) emphasized cell division and DNA replication (Table S4). Using the Network of Cancer Genes (NCG) repository [62] and MII-unique DEGs, our analysis revealed a TGFβ-responsive 16-gene cancer signature capable of distinguishing BRCA from other malignancies and from normal counterparts (Fig. 3G, H, Fig. S3J). Expression of these 16 genes is elevated in primary and metastatic BRCA compared to normal breast, predicting an unfavorable outcome for patients (Fig. 3I, J). Finally, using the clinically-relevant PAM50-gene signature [63] and a pre-trained statistical model [64], we assessed the molecular subtypes of MI and MII cultures before and after TGFβ stimulation. As a positive control, the PAM50 signature extracted from the published transcriptomic profile of MDA-MB-231 basal BRCA cells [40] showed a strong match to basal breast tumors, whereas MI- and MII-spheres, regardless of TGFβ exposure, matched normal breast tissue (Fig. S3K). This observation suggests that HRAS transformation plus TGFβ stimulation are not per se sufficient oncogenic signals to predict full breast malignancy. Overall, TGFβ elicits both common EMT/metabolic responses and distinct cell division and DNA replication/repair gene activation in HRAS-transformed mammary spheres.

### TGFβ positively regulates cell cycle genes in MII-spheres

TGFβ stimulation in MII-spheres upregulated key cell cycle genes (*ORC1*, *BRCA1*, *CDK1*, *CCNB1*, *CCND1*), while *p21* remained unchanged, as validated by qRT-PCR (Fig. S4A). Time-course analysis in MI- and MII-spheres demonstrated negative (MI) and positive (MII) effects on *CCNB1*/*CCND1* expression (Fig. S4B, C), which was validated by EdU-assay in MII-spheres, since enhanced DNA synthesis was measured upon TGFβ pre-stimulation (Fig. S4D, E). As a related confirmation, transcriptomics from doxorubicin-resistant MCF-7 BRCA cells [65] showed elevated TGFβ and EMT gene signatures (Fig. S4F), while MII-spheres co-treated with doxorubicin and TGFβ enhanced the levels of *BRCA1*, *CDK1* and *CCNB1* (Fig. S4G). As further validation, RNA-seq analysis of TNBC MDA-MB-231 cells after transient depletion of SMAD2, SMAD3 and SMAD4 (SMAD2/3/4), and network interaction analysis revealed downmodulation of mitotic phase transition, cytokinesis and DNA double-stranded breaks (Fig. S4H), providing further evidence of the role of SMAD signaling in promoting cell cycle-related processes in a different BRCA model.

### TGFβ signaling transcriptionally modulates cell cycle drivers through SOX4

**Fig. 4:**
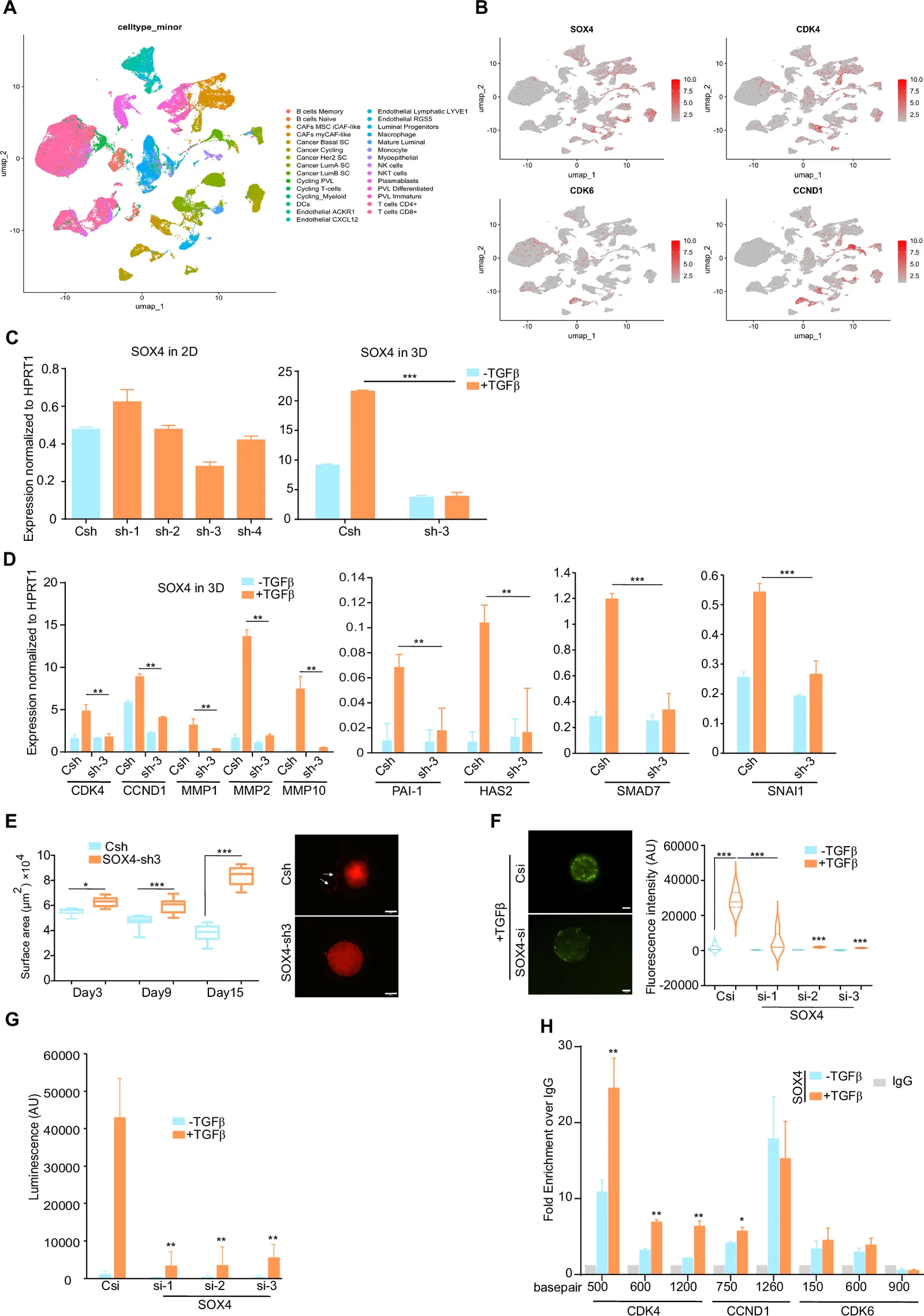
SOX4 is required to promote transcriptional activation of cell cycle genes in response to TGFβ stimulation. UMAP plots visualizing different cell populations detected in 26 BRCA tissues based on scRNA-seq analysis (**A**) and the expression patterns of *SOX4*, *CDK4*, *CDK6* and *CCND1* in multiple cell populations (**B**). The color-coded scales in **B** represent the normalized scaled RNA expression. **C** RT-qPCR analysis of the knockdown efficiency of *SOX4* in MII-cells growing in 2D (left panel) and 3D (right panel) conditions, stably expressing short hairpin scrambled control sequence (Csh) or SOX4-specific sequences (sh-1-4). **D** RT-qPCR analysis of the expression levels of the indicated genes in Csh and SOX4 stable knockdown (sh-3) MII-spheres upon TGFβ stimulation. Values in **C** and **D** represent mRNA expression levels normalized to *HPRT1*. Data in **C** are presented as mean values of three technical replicates ±SD and two independent biological replicates, each with three technical replicates ± SEM in **D**. **E** Quantification of the surface area covered by invading cells from 3D spheres of Csh and SOX4-sh3 cells, measured from at least six independent biological replicates for each condition, is shown on the left panel as a box plot with median values and whiskers representing minimum and maximum values, and with representative microphotographs on the right panel. (Scale bar, 100 µm). **F** Representative fluorescent micrographs of MII-spheres stably expressing CAGA-eGFP reporter transiently depleted of *SOX4* (left panel) and the corresponding quantification (right panel) following TGFβ stimulation for 24 h. (Scale bar, 50 µm). **G** Bar graph demonstrating the luciferase activity measured by luminescence in MII-spheres stably expressing the (CAGA)_12_ reporter and transfected with control siRNA (Csi) or SOX4-specific siRNAs (si-1 – 3) following TGFβ stimulation for 6 h. The data in **E** and **F** were collected from six independent biological replicates. **H** CUT&RUN assay followed by qPCR validating the enrichment of SOX4 protein at different regions, indicated in base-pairs on the X-axis, upstream to the TSS of the investigated genes in MII-spheres treated with TGFβ for 16 h. The data represent the average fold-enrichment ± SEM over IgG derived from two independent biological replicates, each with three technical replicates. Statistical significance in **C–H** was derived using a two-tailed unpaired Student’s *t* test. *p*-values *p ≤ 0.05, **p ≤ 0.01, ***p ≤ 0.001, NS, not significant.

ATAC-seq-based motif analysis identified SOX4 as one of the MII-selective TGFβ-responsive genes. Using the cancer cell line encyclopedia, we confirmed consistent *SOX4* expression across 59 BRCA cell lines (Fig. S4I), while BRCA-TCGA analysis revealed slightly higher *SOX4* levels in basal tumors (Fig. S4J). *SOX4* correlated positively with TGFβ and EMT gene signatures in BRCA (Fig. S4K, L). Furthermore, we reprocessed publicly available scRNA-seq datasets of spatially-resolved tissues from 26 BRCA patients representing the major subtypes [66]. UMAP clustering distinguished cycling cells and cancer-associated fibroblasts (CAFs) among other cells (Fig. 4A). *SOX4* was predominantly expressed in CAFs, basal and cycling cancer cells, matching the cell-type distribution of *CDK4*, *CCND1* and, to a lesser extent, *CDK6* (Fig. 4B, Fig. S4M). Corroborating its presence in BRCA cycling cells, the low *SOX4* expression in 2D cells increased (>15-fold) upon sphere formation, which was further enhanced by TGFβ treatment, and effectively silenced by shRNA in spheres (Fig. 4C). *SOX4* depletion abolished TGFβ-induced expression of cell cycle, EMT and ECM genes and inhibited cell dissemination while preserving viable spheres for extended periods (Fig. 4D, E). The latter observation recapitulated the effect of extended exposure to the TβRI inhibitor LY2157299/galunisertib (Fig. S4N).

To substantiate the necessity of SOX4 for core TGFβ/SMAD signaling, MII-cells were engineered with reporter systems that monitor SMAD-dependent transcriptional activation through eGFP (CAGA-eGFP) and firefly luciferase (CAGA-Luc) expression. TGFβ stimulation activated both reporters, but transient *SOX4* silencing via siRNA abolished their activity (Fig. 4F, G), suggesting that SOX4 may transactivate TGFβ target genes through minimal SMAD-bound sequences and also via direct promoter binding. As validation of the latter, CUT&RUN assays in MII-spheres monitoring promoter-TSS regions predicted to contain SOX4-binding motifs (Fig. S4O), revealed SOX4 enrichment at the *CDK4* promoter, which intensified after TGFβ stimulation, and less evident enrichment at *CCND1* and *CDK6* (Fig. 4H). These results support a model in which TGFβ signaling activates cell cycle (and other) genes via SOX4-driven transcription.

### Cooperative transcriptional activation between SOX4 and SMAD3

**Fig. 5:**
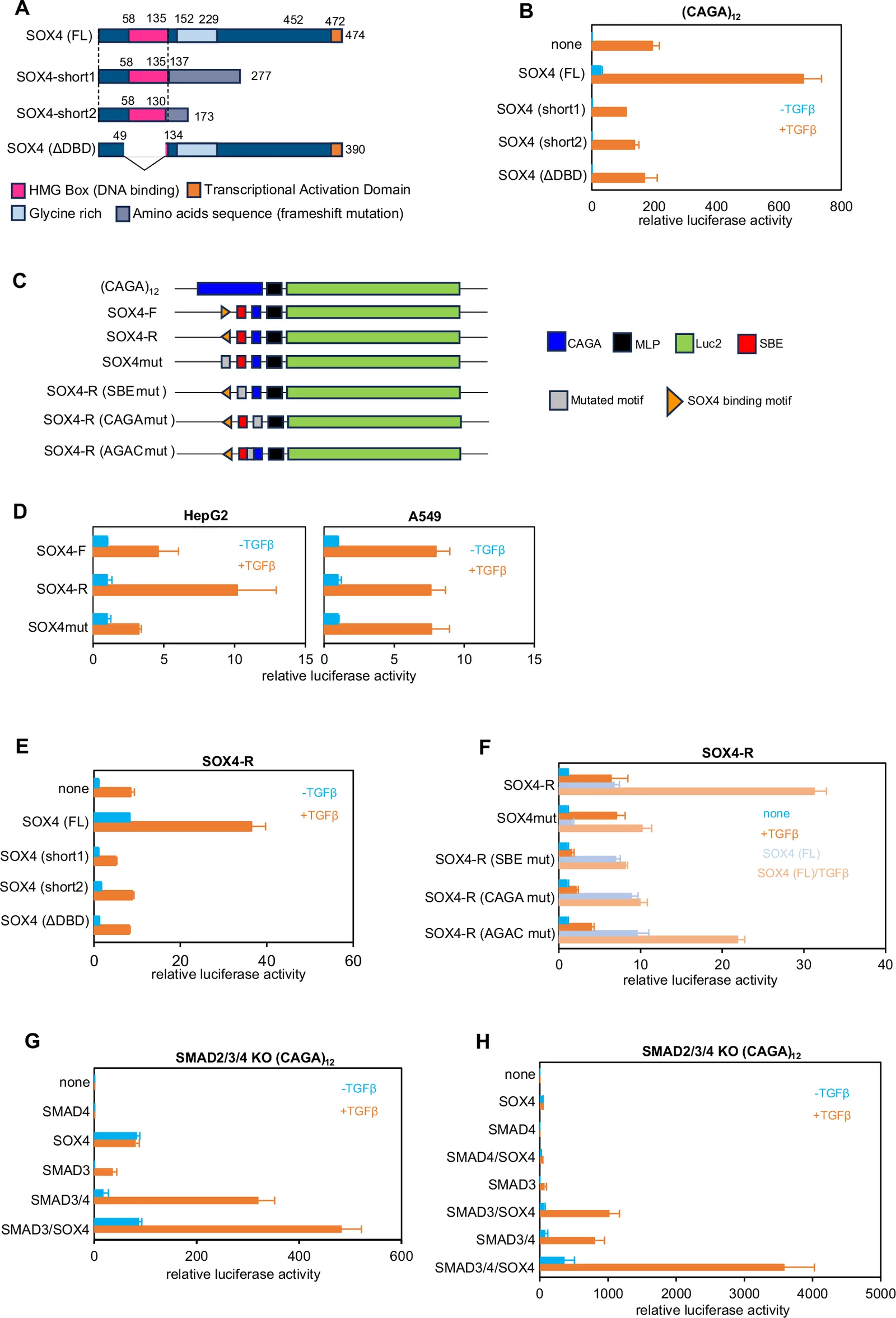
SOX4/SMAD cooperative transcriptional activation. **A** Schematic representation illustrating the structure of SOX4 full-length (FL) protein, SOX4 short isoforms (short1 and short2) and a SOX4 construct harboring a DNA binding domain deletion (ΔDBD). The numbers indicate the amino acid positions. **B** Quantification of the relative luciferase activity in A549 cells co-transfected with (CAGA)_12_ reporter and the indicated constructs in the presence or absence of TGFβ stimulation for 6 h. **C** Schematic representation of (CAGA)_12_ reporter and different constructs harboring the human SOX4 consensus binding motif cloned in the forward orientation (*SOX4*-F), reverse orientation (*SOX4*-R), mutated SOX4 motif (*SOX4*mut) and mutated SMAD binding element (SBEmut). **D** Quantification of the relative luciferase activity in HepG2 and A549 cell lines transfected with the indicated reporters harboring the human SOX4 motif. **E** Relative luciferase activity detected in A549 cells co-transfected with *SOX4*-R reporter and the indicated overexpression constructs upon TGFβ stimulation. **F** Co-transfection in A549 cells with *SOX4*-R reporter and the indicated reporters harboring functional or mutated (mut) SBE and CAGA binding elements in the presence or absence of SOX4 FL and TGFβ. **G**, **H** Measurement of luciferase activity in SMAD2/3/4 triple-knockout (KO) A549 cells co-transfected with (CAGA)_12_ reporter and the indicated constructs. The data in **B** and **D**–**H** represent the average fold-enrichment ± SEM derived from three independent biological replicates, each with three technical replicates.

We consolidated the *SOX4*-silencing results using gain-of-function experiments. We cloned the full-length (FL) *SOX4* transcript from our cDNA library of human U2987MG (glioblastoma) cells, and detected FL-*SOX4* encoding wildtype, 474 amino acid-long protein, and even shorter isoforms in U2987MG and human HepG2 (hepatocellular carcinoma) cells, with short1 harboring a 410 bp deletion, encoding 277 amino acids, and short2 harboring a 722 bp deletion, encoding 173 amino acids (Fig. S5A, B), probably due to frameshift mutations in the investigated cells that generated premature stop codons in the mono-exonic transcript. Both short1/2-SOX4 contain the DNA-binding domain (DBD) HMG-box but lack the C-terminal transactivation domain (TAD) (Fig. 5A). Subsequently, we co-transfected human lung adenocarcinoma A549 cells, because they express very low endogenous SOX4, either with FL, short1, or short2 *SOX4* along with the (CAGA)_12_ reporter introduced above (Fig. 4G), which contains SMAD-binding but lacks SOX4-binding sequences. Upon TGFβ stimulation, only FL-*SOX4* triggered luciferase activity; deletion of the DBD (ΔDBD) impaired this response (Fig. 5A, B).

To test whether SOX4 mediates its functions in a SMAD-dependent manner, reporters that monitor SOX4-SMAD cooperative transcriptional activation were constructed. Hence, the *SOX4* consensus sequence was cloned either in a forward/*SOX4*-F or reverse/*SOX4*-R orientation, upstream to SMAD-binding element (SBE) and CAGA sequences (Fig. 5C). In SOX4-low A549 cells, TGFβ increased luciferase activity, while *SOX4*-R showed higher activity than *SOX4*-F in SOX4-rich HepG2 cells (Fig. 5D). Mutating the SOX4-binding site/*SOX4*mut, drastically reduced reporter response to TGFβ, highlighting the requirement for intact SOX4 motifs in cooperative transcriptional activation. Co-transfection experiments using SOX4 variants with the *SOX4*-R reporter in A549 cells (Fig. 5E) revealed that only FL-*SOX4* (blue bar) enhanced reporter activity, especially when combined with TGFβ, while truncated forms were ineffective. Co-transfecting FL-*SOX4* with either short1 or ΔDBD into cells expressing (CAGA)_12_ or *SOX4*-R reporters, we observed enhanced luciferase activity upon TGFβ treatment, whereas short1 could act as a dominant recessive over FL-*SOX4*, especially on the (CAGA)_12_ reporter (Fig. S5C). Moreover, by mutating the SMAD-binding element (SBEmut) or the CAGA (CAGAmut) motif in the *SOX4*-R reporter, reduced luciferase activity under TGFβ, whereas FL-*SOX4*, independently from TGFβ, transactivated the SBEmut or CAGAmut, but not the SOX4mut reporter (Fig. 5F). To elucidate the cooperative effect of SOX4/SMAD, we utilized a triple-knockout A549 cell line lacking SMAD2/3/4; only SMAD3 (not SMAD4) rescue induced the (CAGA)_12_ reporter in stimulated cells, and SOX4 alone triggered activation regardless of TGFβ (Fig. 5G). Moreover, co-expression of SMAD3 and SOX4 was as effective as the SMAD3/SMAD4 pair, and the SMAD3/SMAD4/SOX4 combination yielded super-activation in triple-knockout cells (Fig. 5H). These findings indicate cooperative transactivation between SOX4 and SMADs in TGFβ signaling.

### Mapping the SOX4 interaction with SMAD3

**Fig. 6:**
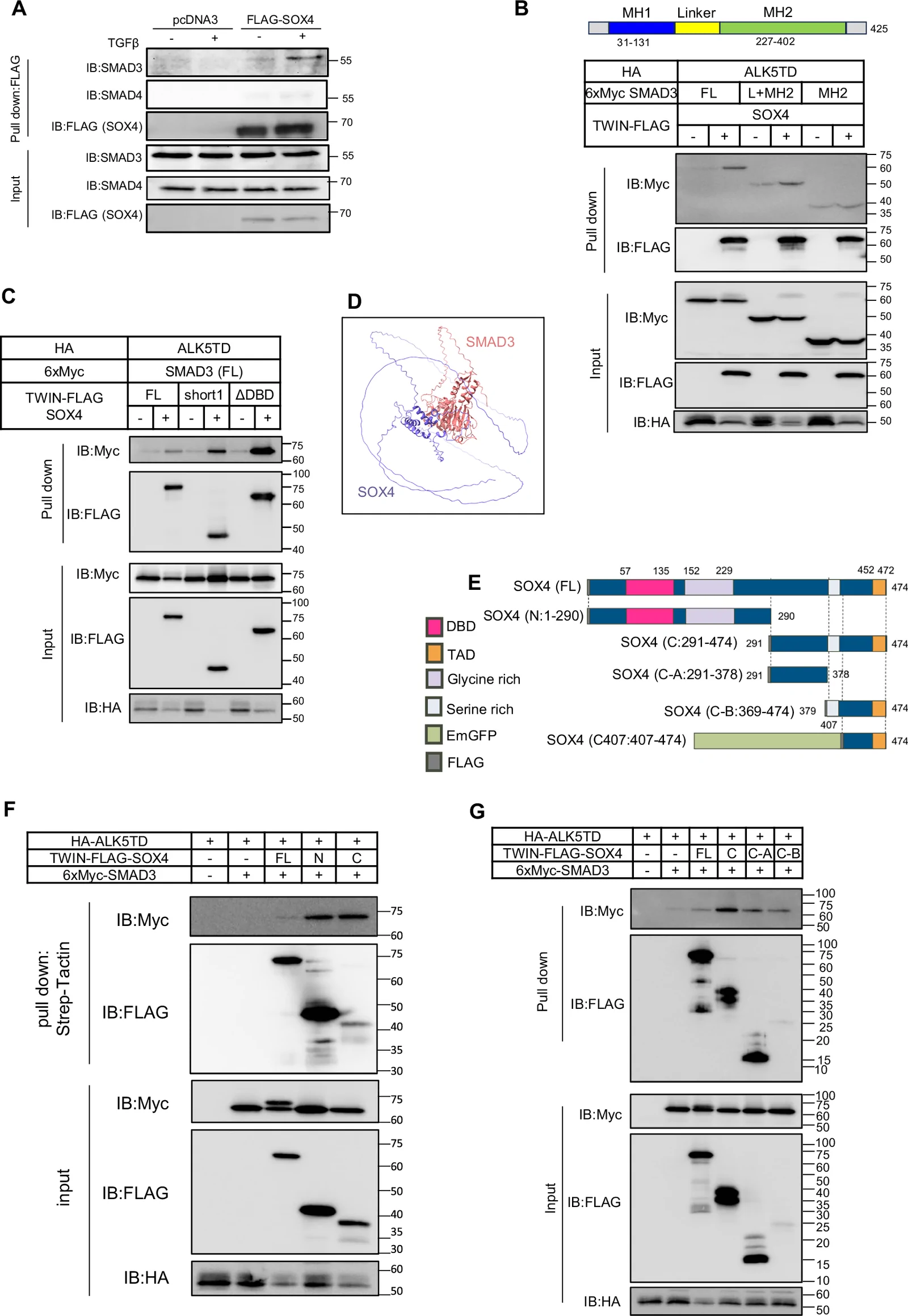
SMAD3, via its MH2 domain, interacts with the intrinsically disordered regions of SOX4. **A** Co-immunoprecipitation (co-IP) of SOX4 in HEK293T cells transfected either with FLAG-tagged SOX4 or empty vector (pcDNA3) followed by immunoblotting (IB) of the indicated proteins in the presence or absence of TGFβ stimulation for 24 h. **B** Schematic representation of the SMAD3 protein with numbers indicating the amino acid residues (upper panel). The lower panel shows co-IP of Myc-tagged constructs expressing SMAD3 full-length (FL), SMAD3 MH2 domain (MH2) alone or fused with the SMAD3 linker (MH2 + L) co-transfected with TWIN-FLAG-tagged SOX4 in HEK293T cells together with an HA-tagged constitutively active TβRI (ALK5TD) followed by IB of the indicated proteins. **C** Co-IP of Myc-tagged SMAD3 in HEK293T cells co-transfected with TWIN-FLAG-tagged SOX4 FL, short1, or ΔDBD constructs, followed by IB. **D** Model of the SOX4-SMAD3 complex predicted by AlphaFold 3. **E** Schematic illustrations of different constructs encoding various fragments of SOX4. **F**, **G** Co-IP of tagged SOX4 FL and different SOX4 fragments in HEK293T cells overexpressing Myc-tagged SMAD3 and an HA-tagged constitutively active TβRI (ALK5TD), followed by IB.

Earlier studies in BRCA cells hinted at a SOX4-SMAD3 interaction via the SMAD3 MH2 domain, excluding SMAD4 and without considering TGFβ signaling [56]. Therefore, we scrutinized these interactions. A weak interaction between SOX4 and SMAD3 in the absence of TGFβ was significantly enhanced in stimulated cells, whereas no reproducible interaction was detected with SMAD4 (Fig. 6A). Co-transfecting HEK293T cells with constitutively active TβRI (ALK5TD), FLAG-SOX4 and Myc-SMAD3 constructs, demonstrated that the MH2 domain alone can interact with SOX4 (Fig. 6B). Furthermore, all SOX4 variants (including FL-*SOX4*, short1, and ΔDBD) interacted robustly with SMAD3 (Fig. 6C), implicating the conserved N-terminal 49 aa SOX4 region (Fig. 5A) in binding.

Structural modeling via AlphaFold3 [67], based on individually resolved crystal structures of SOX4 and SMAD3 [68, 69], did not demonstrate robust interaction surfaces with high confidence, but suggested flexible intrinsically disordered regions (IDRs) flanking the SOX4 HMG domain (Fig. 6D). Therefore, we hypothesized that SMAD3 may interact with both N- and C-terminal SOX4 IDRs, which was confirmed, especially with regions C-A (291–378 aa) and C-B (369–474 aa) (Fig. 6E-G). The extreme C-terminal SOX4 region (407–474 aa) was not essential (Fig. S6A). In addition, structurally predicted putative phosphorylation sites at the C-terminal SOX4 IDR serine390/391 residues may regulate the SOX4 interaction surface with SMAD3 (Fig. S6B). These analyses indicate that the N- and C-terminal SOX4 IDRs can mediate interactions with SMAD3, in agreement with established functions of IDRs in transcription factor specificity [70].

### TGFβ promotes SOX4-mediated resistance to the CDK4/6 inhibitor palbociclib

**Fig. 7:**
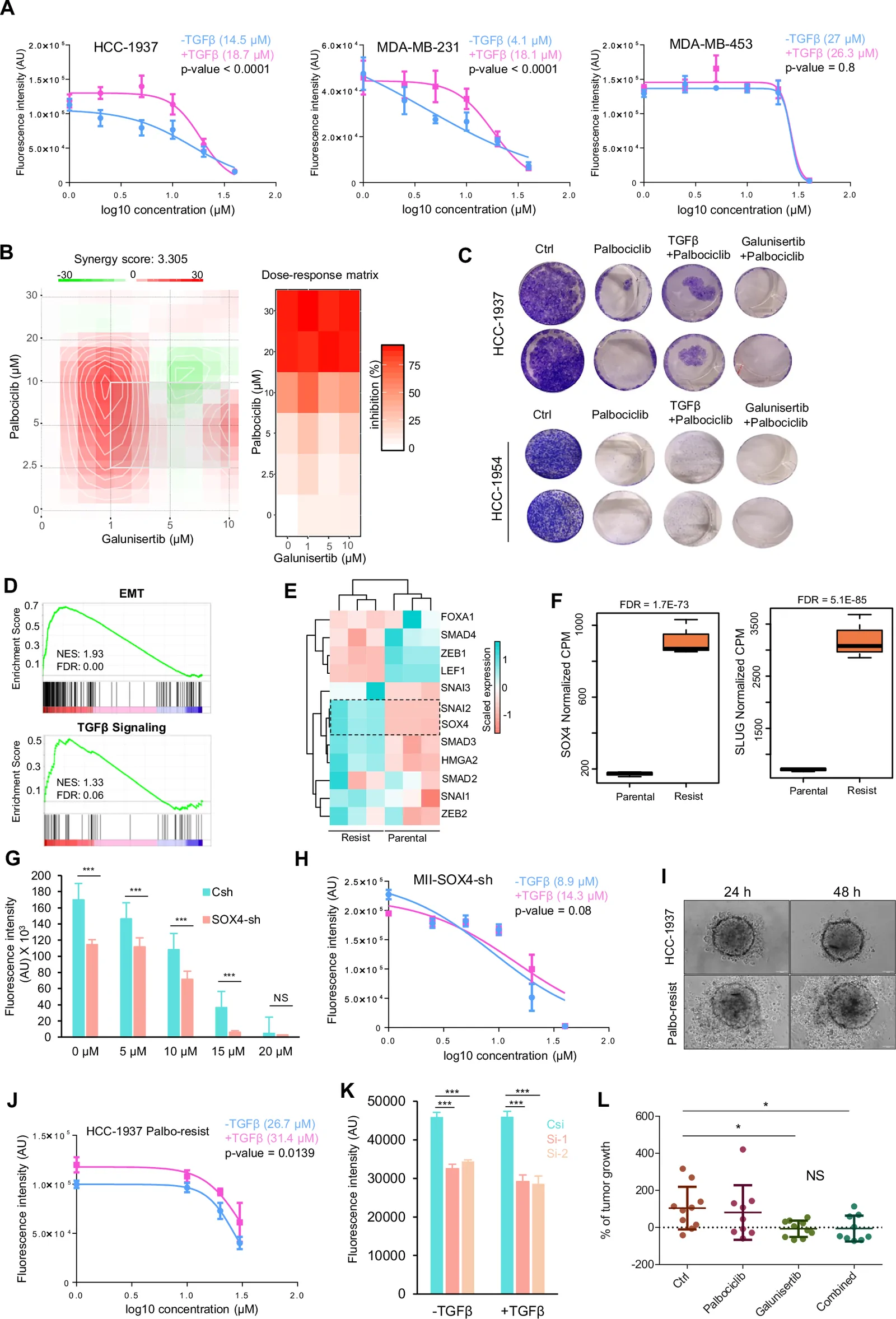
TGFβ exploits SOX4 function to promote resistance to palbociclib treatment in BRCA cells. **A** Dose–response curves showing the IC_50_ of palbociclib following 72 h of treatment in the investigated cell lines in the presence or absence of TGFβ (2 ng/ml). The X-axis represents the log10 values of palbociclib concentrations used in µM units. Fluorescence intensity on the Y-axis corresponds to the relative cell density, indicating the viable cell counts. The pink and blue lines represent the non-linear regression line fitted on the data points with or without TGFβ stimulation, respectively. The IC_50_ values corresponding to each experimental condition were calculated based on non-linear regression analysis, and are shown in brackets. The *p*-values represent the statistical significance demarcating the difference between the IC_50_ values in each condition. **B** Synergy and dose-response matrix heatmaps showing the effect of combining different concentrations of palbociclib and galunisertib on the viability of HCC-1937 cells following 72 h of treatment. The synergy score value > 10 represents a synergistic effect, while a value < −10 indicates an antagonistic effect, and values ranging from 0–10 indicate an additive effect. **C** Colony-forming assay with HCC-1937 and HCC-1954 cells, either treated with DMSO as a control (Ctrl), 10 µM of palbociclib, or in combination with TGFβ (2 ng/ml) or galunisertib (3 µM) for 2 weeks. **D** GSEA plots of significantly enriched hallmarks in palbociclib-resistant MDA-MB-231 cells compared to parental cells. **E** Heatmaps showing the expression patterns of selected EMT-related transcription factors in MDA-MB-231 palbociclib-resistant (resist) and parental cells based on RNA-seq analysis. The dotted rectangle highlights *SOX4* (log2-FC = 2.4, FDR = 1.7E-73) and *SLUG* (*SNAI2*; log2-FC = 2.18, FDR = 5.1E-85). **F** Box plots indicating the expression levels of *SOX4* and *SLUG* in parental and resistant MDA-MB-231 cells. The data are expressed as normalized counts per million (CPM) and the false discovery rate (FDR) was calculated based on the differential expression analysis obtained from three independent replicates of each condition. **G** Bar graph showing the relative cellular viability of MII-cells stably expressing scrambled control sequence (Csh) or SOX4 shRNA (SOX4-sh) exposed to variable concentrations of palbociclib for 72 h. Data represent an average of eight biological replicates ± SEM. Statistical significance was derived using a two-tailed unpaired Student’s *t*-test. **H** A dose–response curve of MII-cells stably depleted of *SOX4* exposed to palbociclib for 72 h in the presence or absence of TGFβ. **I** Phase contrast micrograph of spheroids derived from parental HCC-1937 and palbociclib-resistant (palbo-resist) HCC-1937 spheres. Images were acquired at 24 and 48 h post-seeding. (Scale bar, 50 µm). **J** Dose–response curve of palbociclib-resistant cells treated with an escalating dose of palbociclib for 72 h. **K** Viability assay in palbociclib-resistant MDA-MB-231cells transfected with control siRNA (Csi) or SOX4-specific siRNAs (si-1 and si-2) for 48 h in the presence or absence of TGFβ stimulation. Data presented as mean of three biological replicates ± SEM. Statistical significance was derived using a two-tailed unpaired Student’s *t* test. **L** Percentage of tumor growth at five days post-fertilization (5 dpf) in zebrafish larvae relative to the tumors measured at 3 dpf following the exposure to palbociclib (20 µM), galunisertib (20 µM) or their combination. DMSO was used as a control vehicle (Ctrl). Data were collected by measuring independent tumors growing in independently injected larvae. Statistical significance in **A,** **H,** **J** and **K** was derived using a one-way ANOVA test with Tukey’s correction for multiple comparisons. *p*-values *p ≤ 0.05, **p ≤ 0.01, ***p ≤ 0.001, NS, not significant.

The observed requirement of TGFβ and SOX4 on expression of CDKs, in addition to previous studies [71], prompted us to investigate the relationship between TGFβ signaling and clinically approved CDK inhibitors, selecting the CDK4/6 inhibitor palbociclib as a test compound. Using a probabilistic integrative database [72], we queried the association between gene expression and differential dose-response to palbociclib in 1092 cancer cell lines, and performed GSEA of the associated genes. TGFβ, EMT and glycolysis gene signatures correlated with higher inhibitory concentration (IC_50_) for palbociclib, while oxidative phosphorylation correlated with increased sensitivity (Fig. S7A). Experimental validations showed that TGFβ raised the palbociclib IC_50_ in BRCA cells, indicating reduced drug efficacy (Fig. 7A, Fig. S7B). On the other hand, hyperproliferative MDA-MB-453 cells, expressing high levels of CCND1 and functionally suppressed retinoblastoma (RB) protein [73], failed to respond properly to palbociclib regardless of TGFβ treatment (Fig. 7A), and showed severe cytotoxicity at higher drug concentrations, confirming the role of RB in palbociclib sensitivity [74].

Similar to the human BRCA cell lines (Fig. 7A), TGFβ stimulation increased the palbociclib IC_50_ in Py2T and MII-spheres, but sensitized MI-spheres to the drug because of its strong anti-proliferative effect, as assessed by cell viability and caspase 3/7 activity (Fig. S7B – G). Testing combined palbociclib and TGFβ inhibition (via galunisertib) revealed a complex drug interaction, yielding additive (but not synergistic) effects on cell viability (Fig. 7B, Fig. S7H), based on a dose–response matrix computed through synergy score calculations [38] in HCC-1937 and HCC-1954 2D cultures. Still, palbociclib/galunisertib combinations reduced colony-forming ability, whereas TGFβ alone promoted sporadic colony survival (Fig. 7C), compatible with its predominant action as a pro-resistance factor.

### Relevance of SOX4 to TGFβ-dependent resistance to palbociclib

To understand the role of TGFβ signaling in promoting resistance to palbociclib, we re-processed available RNA-seq datasets of palbociclib-resistant MDA-MB-231 cells [75], where GSEA demonstrated enrichment of EMT, angiogenesis and TGFβ signatures in resistant cells (Fig. 7D, Table S5). DEA of EMT-related TFs showed significant upregulation of *SOX4* (and *SLUG* (*SNAI2*) in resistant cells (Fig. 7E, F). *SOX4*-deficient MII-cells were more sensitive to palbociclib and unresponsive to TGFβ-driven resistance, suggesting SOX4 as a key modulator (Fig. 7G, H). To validate these findings, we exposed BRCA cell lines (HCC-1937, HCC-1954 and MDA-MB-231) to escalating palbociclib doses over a 6-month period, thus developing palbociclib-resistant cells, characterized by slower growth, structural disorganization of 3D HCC-1937 spheres, with faster migratory behavior and altered clonogenic morphology (Fig. 7I, Fig. S7I). Palbociclib-resistant cells exhibited elevated TGFβ signaling, including *SOX4* and *SLUG* expression (Fig. S7J – L). TGFβ further reinforced resistance, while its inhibition with galunisertib suppressed gene expression and restored palbociclib sensitivity, reducing clonogenic potential even at lower drug concentrations (Fig. 7J, Fig. S7M, N). Expectedly, transient knockdown of SOX4 reduced the proliferation of resistant cells (Fig. 7K, Fig. S7O).

We utilized zebrafish embryos that allow rapid, medium-throughput drug screening. Bloodstream injection of resistant BRCA cells (HCC-1937 (Fig. S8A) and MDA-MB-231) led to cardiac edema and mortality, while PVS-injected embryos formed micrometastatic tumors that attracted melanocytes and caused 70% mortality at 48 h (Fig. S8B), potentially linked to elevated cytokine activity. Optimized yolk-sac engraftment produced solid tumors without adverse effects (Fig. S8C). Drug tolerance assays confirmed larval safety at up to 40 µM palbociclib and 80 µM galunisertib, with minor defects in the yolk and bladder areas (Fig. S8D). Tumor-bearing embryos exposed to these treatments for 48 h (5 dpf) revealed that galunisertib alone significantly suppressed tumor growth, whereas palbociclib showed no substantial effect (Fig. 7L, Fig. S8E). Combined treatment offered no added benefit, underscoring the potency of TGFβ inhibition in reducing tumor burden in vivo.

### Ambiguous contribution of SLUG in regulating SOX4 expression

**Fig. 8:**
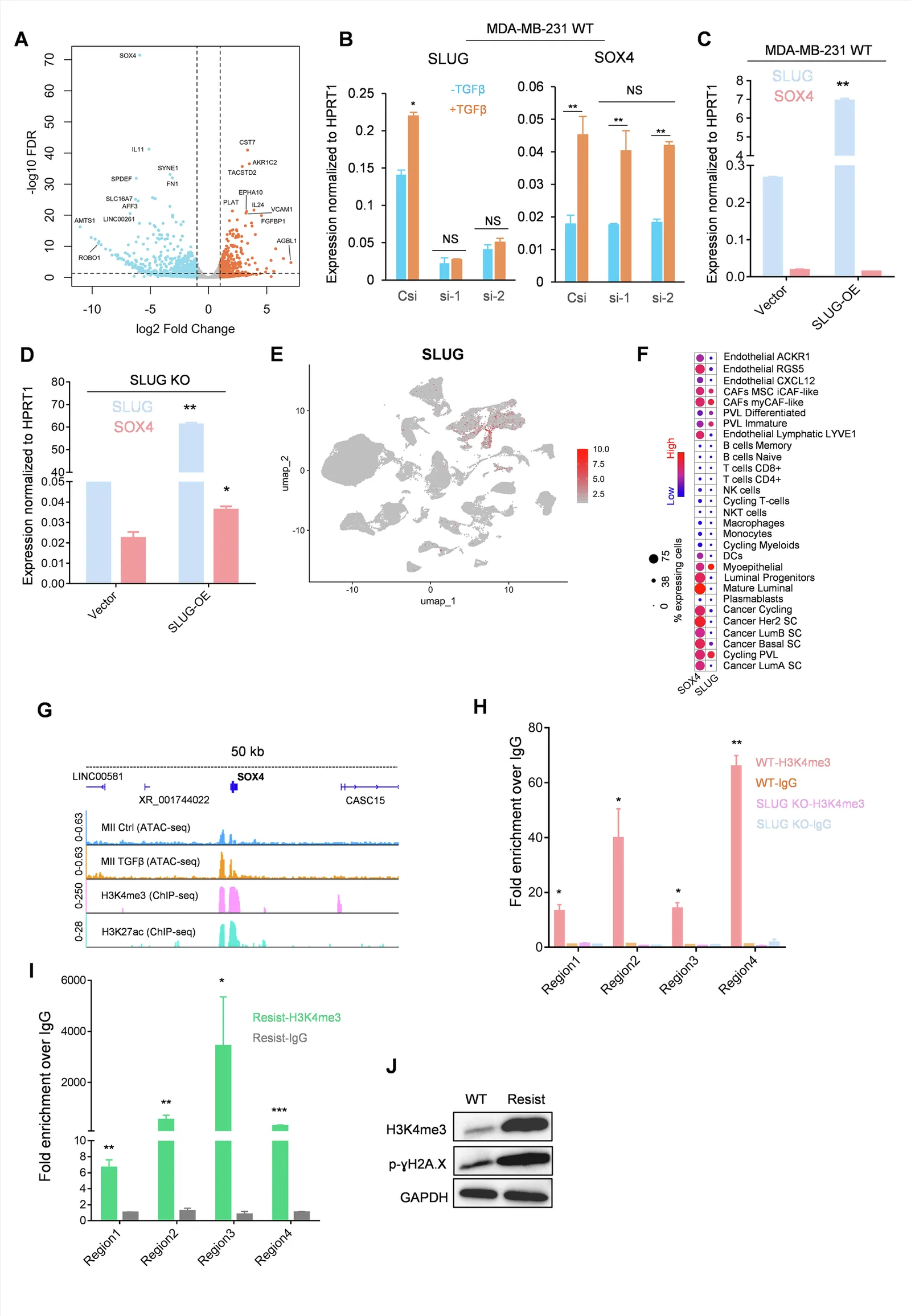
Epigenetic regulation of SOX4 in palbociclib-resistant cells. **A** Volcano plot showing the DEGs based on RNA-seq analysis of *SLUG* knockout (KO) MDA-MB-231 cells compared to parental cells. **B** RT-qPCR of *SLUG* and *SOX4* expression levels in MDA-MB-231 cells transfected with scrambled control (Csi) or *SLUG*-specific siRNAs (si-1 and si-2) in the presence or absence of TGFβ stimulation for 24 h. Data represent the average of three technical replicates ± SD. RT-qPCR of *SLUG* and *SOX4* expression levels in MDA-MB-231 parental cells (**C**) and *SLUG* KO cells (**D**) transiently over-expressing SLUG (SLUG-OE) or an empty vector. Data represent the average of two independent biological replicates ± SEM. UMAP visualization of *SLUG* expression in different cell populations (**E**) with the corresponding percentage of expressing cells (**F**) based on scRNA-seq analysis of 26 BRCA tissues. **G** Genome browser snapshot showing ATAC-seq peak distribution at the *SOX4* genomic locus in unstimulated or TGFβ-stimulated MII-spheres and ChIP-seq peak distributions of H3K4me3 and H3K27ac active marks. The values on the Y-axis represent the normalized scale of each condition. ChIP-qPCR of H3K4me3 active mark at the upstream regions of the *SOX4* TSS, indicated on the X-axis, in parental MDA-MB-231 and *SLUG* KO cells (**H**) and palbociclib-resistant MDA-MB-231 cells (**I**). The data represent the average fold-enrichment ± SEM over IgG derived from two independent biological replicates, each with three technical replicates. **J** Immunoblotting of the indicated proteins in parental wildtype (WT) MDA-MB-231 and resistant (resist) cells. Statistical significance in **B–D,** **H** and **I** was derived using a two-tailed unpaired Student’s *t* test. *p*-values *p ≤ 0.05, **p ≤ 0.01, ***p ≤ 0.001, NS, not significant.

The elevated *SLUG* levels in palbociclib-resistant cells suggested that *SOX4* expression might be elevated due to SLUG and we also queried a similar role for SNAIL/SNAI1. Our previous *SNAI1* knockout (KO) RNA-seq analysis did not support regulation of *SOX4* expression by SNAI1 in MDA-MB-231 cells [40]. CRISPR/Cas9-mediated KO of *SLUG* in MDA-MB-231 cells identified *SOX4* as the downregulated gene with the highest confidence (Fig. 8A, Table S6), suggesting potential regulatory links. However, transient *SLUG* knockdown in MDA-MB-231 or MII cells, regardless of TGFβ stimulation (Fig. 8B, Fig. S9A, B), or overexpression in MDA-MB-231 cells (Fig. S9C), did not alter *SOX4* expression, and TGFβ stimulation still upregulated *SOX4* regardless of *SLUG* status (Fig. 8B, Fig. S9A, B). SLUG rescue restored SOX4 (Fig. 8D), but overall, SLUG appeared dispensable for *SOX4* transactivation. *SLUG* KO cells also showed reduced viability, yet SOX4 overexpression could not rescue this or *SLUG* levels (Fig. S9D – F). Finally, spatial distribution of *SLUG* expression in BRCA tissues indicated abundant expression in CAFs and myoepithelial cells, unlike *SOX4*, which was predominantly expressed in carcinoma cells (Figs. 4A, 8E, F). Querying the scRNA expression patterns of *SLUG* and *SOX4* in normal breast tissues, publicly available through the Human Protein Atlas portal [76], showed distinct patterns, fibroblastic *SLUG* expression and glandular or myoepithelial cell expression for *SOX4* (Fig. S9G, H). These findings indicate that SOX4 is regulated by TGFβ independently of SLUG.

### Epigenetic SOX4 regulation in naïve and resistant BRCA cells

The MII-sphere ATAC-seq data indicated enhanced chromatin accessibility at two regions upstream of the *SOX4* TSS upon TGFβ stimulation, coinciding with active (H3K4me3/H3K27ac) chromatin marks obtained from ENCODE re-processed ChIP-seq datasets [77] (Fig. 8G), suggesting a chromatin locus-specific response. Using overlapping ChIP primers upstream of the *SOX4* TSS, strong H3K4me3 enrichment at these sites was scored in parental MDA-MB-231 cells, but *SLUG* KO abolished these marks (Fig. 8H). Strikingly, palbociclib-resistant cells exhibited increased H3K4me3 levels across the *SOX4* locus, despite the accumulation of DNA damage indicated by increased levels of phosphorylated histone variant ɣH2A.X, an established marker of damaged DNA, which was further evidenced by a global increase in H3K4me3 in resistant cells (Fig. 8I, J), reflecting the elevated *SOX4* expression in such cells. These data point to epigenetic effects during TGFβ-induced *SOX4* transactivation of the locus. Nevertheless, further investigations are needed to identify specific chromatin remodelers required for the transactivation of *SOX4* in palbociclib-resistant cells, as proposed by the current data that generate a stimulating hypothesis.

## Discussion

In a premalignant milieu, TGFβ preserves tissue homeostasis and suppresses inflammation [78]. Under malignant conditions, TGFβ facilitates a pro-tumorigenic environment [79]. Although loss-of-function mutations in signaling mediators explain loss of cytostatic functions of TGFβ, gradually uncovered factors seem to be responsible for the pro-oncogenic action of TGFβ [79]. In this regard, we investigated TGFβ signaling in isogenic breast cell lines that differ by HRAS mutation that confers carcinogenic potential in vivo [51]. Culturing cells under 3D conditions better preserves tumor tissue architecture in terms of cell-cell interactions and the generation of a hypoxic center. Yet 3D cultures of single cell types do not match the complexity of a real tumor with its microenvironment, tumor-embedded blood vessels and infiltrating immune cells. Despite these deficiencies, the epigenetic impact of oncogenic mutations in carcinoma cells can be preserved under 3D culture conditions, making such studies useful and hypothesis-generating that can then be translated to human tumor contexts [80].

TGFβ altered thousands of transcripts showing similar expression patterns in MI- and MII-spheres. The 524 commonly regulated RNAs in MI- and MII-cells represent mainly the fibrogenic program [79] of extracellular matrix pathways. MI-specific genes were enriched in metabolic pathways, with unique MII-DEGs being enriched in cell cycle checkpoints, DNA replication and chromosome segregation, suggesting a novel cooperation of TGFβ with the HRAS-induced phenotype. Previous demonstrations of the cooperation between TGFβ and oncogenic RAS have primarily addressed the process of EMT and tumor metastasis [24], including a unique transcriptional, coupled to chromatin remodeling, mechanism, driven by the RAS-dependent factor RREB1 [25].

NCG analysis identified 16 MII-specific TGFβ-responsive genes, forming a BRCA-specific signature that includes well-known tumor drivers (*BRCA1*, *BRCA2*, *IL1A*, *KIF14*) [81–83] and genes acting in different tumors (*PTCH2*, *RPGRIP1*), providing a new diagnostic tool based on TGFβ’s oncogenic influence. Additionally, *VEPH1*, enriched in this signature, can inhibit TGFβ signaling by impeding the release of SMAD2 from the TβRI [84]. Our data demonstrating induction of *VEPH1* by TGFβ in MII-spheres may underscore the generation of a negative feedback loop.

TGFβ stimulation enhanced chromatin accessibility in both MI- and MII-spheres, though these changes did not directly mirror transcriptional patterns associated with the clinically relevant PAM50 BRCA subtypes [63], suggesting that phenotypic responses to TGFβ are not strong enough to influence switches between BRCA subtypes. In contrast, genetic silencing of EMT transcription factors such as SNAI1 can switch BRCA phenotype between different subtypes [40]. Since DNA accessibility at promoter regions is not the sole determinant of gene expression [85], our accessibility analysis focusing on promoter-TSS regions might not capture all important genomic loci. For example, placental development studies revealed a high concordance between promoter accessibility and expression of housekeeping genes, and discordance with tissue-specific genes [86]. Analyses in MCF-7 BRCA cells identified genes with limited baseline chromatin accessibility near their promoters, that was enhanced upon TGFβ treatment, whose function is associated with cell differentiation and lineage plasticity [87]. In contrast, genes with discordant chromatin accessibility and expression profiles demonstrated higher chromatin accessibility irrespective of TGFβ stimulation and were enriched in signal transduction pathways [87]. Furthermore, enhancement of accessibility at enhancers was measured in the mammospheres; however, the correlation between enhancer accessibility and transcriptional activation is not linear, since multiple enhancers can regulate a single gene, and a single enhancer can regulate multiple genes [88], suggesting the need for deeper analysis for the role of enhancers under TGFβ signaling.

SOX4 motif enrichment in TGFβ-stimulated MII-spheres became a central focus of this study, despite prior claims of its limited involvement in transformation [89]. SOX4 promotes cell aggression characterized by high mutational burden and loss of crucial tumor suppressor genes [90]. Amplified in ~10% of TNBC, SOX4 correlates with metastasis [91], and promotes cancer stemness and cell cycle progression [92, 93]. Moreover, scRNA-seq analysis of BRCA tissues revealed remarkably similar expression patterns of *SOX4*, *CDK4* and *CCND1* in cancer-cycling cells and luminal progenitors. This agrees with SOX4 regulation of progenitor cell cycle programs in cycling murine BRCA cells [94], which show enrichment of E2F-, Myc-regulated genes and G2/M cell cycle hallmarks, similar to our analysis of TGFβ-stimulated MII-spheres. In agreement with previous reports focusing on EMT [95], we show that *SOX4* depletion downmodulated TGFβ-induced cell cycle genes in human MII-spheres, effects intensified by galunisertib treatment. Additionally, *SOX4* silencing impaired the transactivation of SMAD-dependent transcription, which is compatible with SOX4 interactions with SMADs in a TGFβ-dependent manner. Although others reported that SOX4 determines the oncogenic selectivity of SMAD3 in regulating EMT-related genes during breast carcinogenesis and SOX4-SMAD3 interaction occurs irrespective of TGFβ stimulation [56], we demonstrate that TGFβ is required to mediate a reproducible SOX4-SMAD3 interaction. Meanwhile, we could not detect SOX4-SMAD4 interaction, which agrees with the previous study [56]. Computational modeling and biochemical validations mapped interactions between the SMAD3 MH2 and the SOX4 IDR domains. Intriguingly, in SMAD2/3/4 triple-knockout cells, SOX4-SMAD3, unlike SOX4-SMAD4, was sufficient to restore TGFβ-SMAD signaling, with the SOX4 DBD, beyond its IRDs, contributing to the SOX4-SMAD3 cooperation. We also mapped short *SOX4* isoforms possibly generated by focal amplification of the 6p21-p23 region, a chromosomal aberration associated with cancer metastasis [96].

Investigating the link between SOX4-regulated cell cycle gene expression and therapeutic response revealed that TGFβ stimulation undermines the efficacy of CDK4/6 inhibitors (CDK4/6i), like palbociclib, that are implemented in first-line monotherapy or in combination with aromatase inhibitors to treat non-resectable BRCA [97]. TGFβ elevated the palbociclib IC_50_ values in various BRCA cell lines but not in non-transformed MI-cells, where it arrests proliferation. Moreover, palbociclib-resistant cells demonstrated elevated levels of TGFβ signaling components, *SOX4* and *SLUG*. Recent studies indicated several mechanisms of acquired resistance to CDK4/6i. For instance, transient palbociclib administration to pancreatic cancer cells activated TGFβ signaling, which induced EMT and the ECM response [30]. Furthermore, *CDK6* genomic amplification occurs in BRCA cells following long-term exposure to CDK4/6i [98], whereas cells harboring *CCND1* translocations, *CCND2*, or *CCND3* amplifications are more sensitive to CDK4/6i [99]. An intriguing mechanism proposed that mutant CDK6 sequesters and inactivates the CDK inhibitor p15, thus counteracting TGFβ-mediated cytostasis [31]. Similarly, *CCNE1*, *AURKA*, *S6K1* amplifications, *c-MYC* overexpression, activating mutations in *RAS*, *AKT1*, *FGFR2* and *CCNE2* and *RB1* loss, facilitate resistance to palbociclib [100–102]. An additional factor contributing to CDK4/6i resistance is the cytokine IL-6, whose elimination resensitizes BRCA cells to palbociclib; IL-6 signals via STAT3 and STAT3 inhibitors have been shown to be effective in reducing BRCA growth in vivo [103]. Moreover, TMEM45A/mTOR signaling confers resistance to palbociclib by inducing EMT and glycolytic shift [104]. Accordingly, we found the mTORC1 signature enriched in TGFβ-stimulated mammospheres, including *TMEM45A* upregulation. These insights propose TGFβ as a contributor to CDK4/6i resistance, with SOX4 potentially acting as an effector in this adaptive process. Accordingly, we showed that TβRI inhibition or SOX4 depletion sensitized BRCA cells to palbociclib and attenuated the acquired resistance.

In contrast, CDK6 overexpression selected during resistance to palbociclib was shown to induce extracellular vesicle-mediated transport of *miR-432-5p* that downregulates SMAD4, and thus, resistance could be transmitted to vesicle-receiving T47D BRCA cells, suggesting that TGFβ signaling counteracts resistance [32]. An alternative BRCA palbociclib-resistant cell model with high CDK6 expression showed that TGFβ3 and palbociclib can be effectively used to eradicate TNBC cells [33]. These two examples open the possibility that TGFβ, similar to its dual role in cancer progression, may also elicit opposite functions in cancer cell resistance to targeted drugs, a topic that deserves deeper analysis.

Beyond CDK4/6i, our experimental validations and analyses of published datasets [65] elucidated potent effects of TGFβ in mediating resistance to doxorubicin-induced DNA damage (e.g., *BRCA1* regulation), compatible with the role of TGFβ in promoting doxorubicin-resistance in lung adenocarcinoma cells through *ALDH1A1* [105]. *ALDH1A1* transcriptional activation was observed in MII- (log2 FC = 1.7, FDR = 0.0002) but not in MI-spheres (Tables S3, S4), suggesting common mechanisms of TGFβ-mediated resistance to doxorubicin.

In agreement with our report, other studies implicated SOX4 in the acquired resistance to hormonal therapy in estrogen-dependent BRCA cells [106] and to cisplatin in cervical cancer and melanoma cells [107, 108]. Moreover, *SOX4* upregulation by activation of latent TGFβ promoted immune evasion and suppressed cytotoxic T-cells in TNBC [109]. Interestingly, in therapy-resistant prostate tumors, the H3K4me1 mark was highly enriched in TGFβ signaling genes, with SOX4 contributions to drug resistance [110]. Similarly, our ATAC-seq and ChIP analyses following TGFβ treatment revealed strong chromatin accessibility in the proximal promoter region of *SOX4* and a potential enhancer element. Thus, our data open the possibility of epigenetic regulation of the *SOX4* locus in palbociclib-resistant BRCA cells.

In conclusion, this study explores context-specific functions of TGFβ, shaped by the oncogenic contribution of HRAS. It demonstrates how TGFβ can promote resistance to the CDK4/6i palbociclib through transactivation by oncogenic SOX4, and introduces the SOX4-SMAD transcriptional module that facilitates adaptation to anti-cancer treatment and survival of fit cancer cells.

## Supplementary information

Table S1

Table S2

Table S3

Table S4

Table S5

Table S6

Table S7

Supplementary Figures

## Data Availability

Original and processed sequencing data files (RNA-seq and ATAC-seq) are deposited to the public repository GEO (accession number GSE300358). Processed nanoString profiling data are provided as a supplementary table.
