# Supplementary material for: TGFβ signaling promotes cell cycle progression and resistance to the CDK4/6 inhibitor palbociclib through SOX4 transcriptional modulation in breast cancer cells
Source: Cell Death Dis. 2026 Feb 4;17(1):209. doi: 10.1038/s41419-026-08435-4 (PMC12895049; doi:10.1038/s41419-026-08435-4)
Supplement: Supplementary file 8 — Supplementary Figures [file 41419_2026_8435_MOESM8_ESM.pdf]

TGFβ signaling promotes cell cycle progression and resistance to the CDK4/6 inhibitor palbociclib through SOX4 transcriptional modulation in breast cancer

Mohamad Moustafa Ali[1], Yuka Itoh[1,2], Aisha Mariama Pereira Badji[1], Sarah Gallant[1], Chrysoula Tsirigoti[1,3], Yu Bai[1], Beata Filipek-Górniok[4], Keiji Miyazawa[2], Carl-Henrik Heldin[1] and Aristidis Moustakas[1]

[1]Department of Medical Biochemistry and Microbiology, Science for Life Laboratory, Box 582, Uppsala University, SE-751 23 Uppsala, Sweden.

[2]Department of Biochemistry, Graduate School of Medicine, University of Yamanashi, Shimokato 1110, Chuo, Yamanashi, 409-3898, Japan.

[3]Astra Zeneca, Pepparedsleden 1, Mölndal, SE-431 83, Sweden.

[4]Department of Genetics and Pathology, Science for Life Laboratory, Uppsala University, SE-751 85 Uppsala, Sweden.

Supplementary table titles

Table S1. Nanostring analysis of gene expression in MI and MII 3D cultures.

Table S2. ATAC-seq data of MI and MII 3D cultures in the absence or presence of TGFβ stimulation.

Table S3. RNA-seq analysis of MI 3D cultures in the absence or presence of TGFβ stimulation.

Table S4. RNA-seq analysis of MII 3D cultures in the absence or presence of TGFβ stimulation.

Table S5. DEGs in Palbociclib-resistant MDA-MB-231 cells.

Table S6. DEGs in *SLUG* knockout MDA-MB-231 cells.

Table S7. qRT-PCR and ChIP primers, antibodies and siRNAs

Supplementary figures, titles and legends

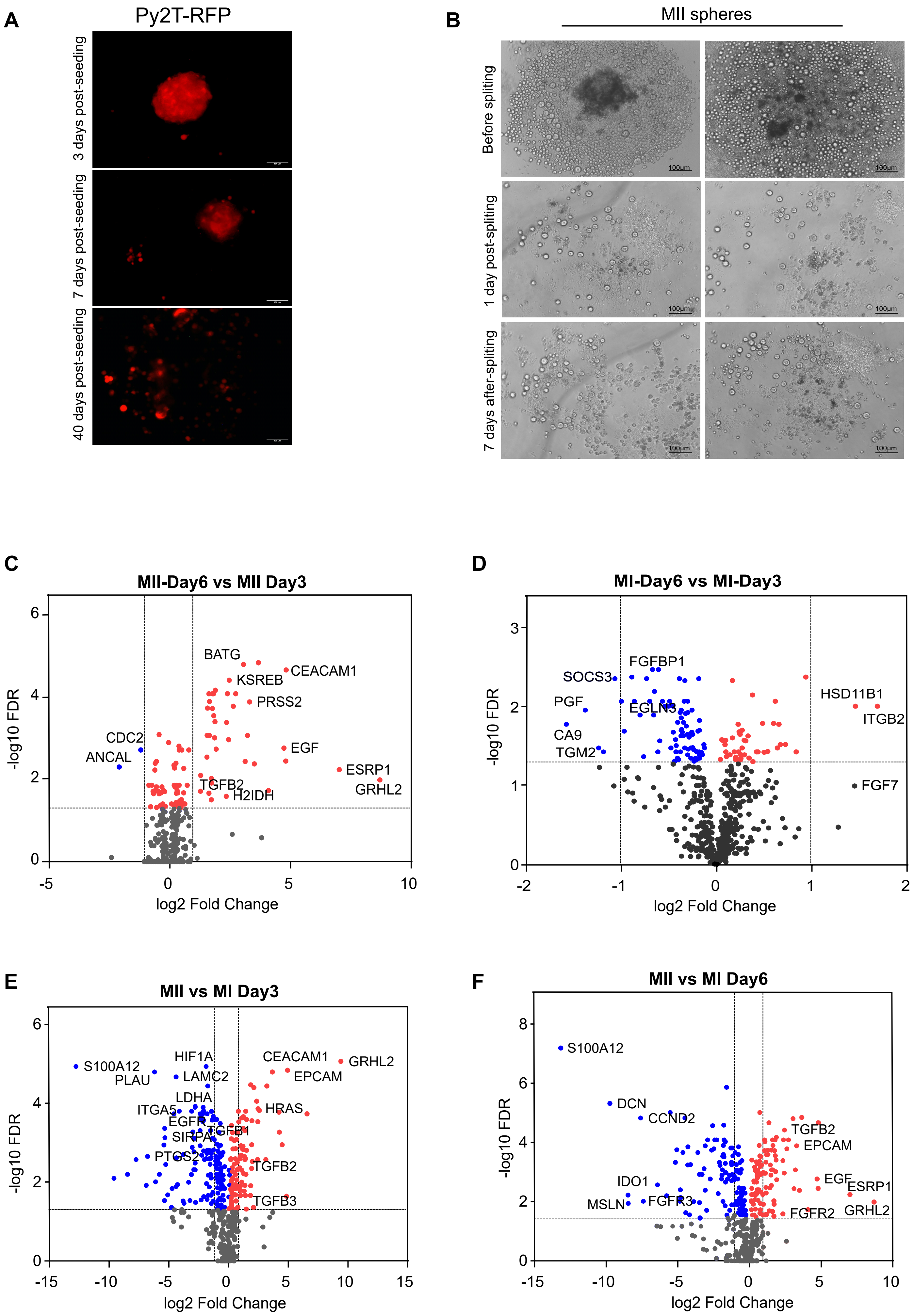
A
Py2T-RFP
3 days post-seeding
7 days post-seeding
40 days post-seeding
B
MII spheres
Before spliting
1 day post-spliting
7 days after-spliting
100μm
C
MII-Day6 vs MII Day3
-log10 FDR
log2 Fold Change
BATG
CEACAM1
KSREB
PRSS2
CDC2
ANCAL
EGF
ESRP1
GRHL2
TGFB2
H2IDH
D
MI-Day6 vs MI-Day3
FGFBP1
SOCS3
PGF
EGLN3
CA9
TGM2
HSD11B1
ITGB2
FGF7
E
MII vs MI Day3
S100A12
PLAU
HIF1A
LAMC2
LDHA
ITGA5
EGFR
SIRPA
TGFB1
PTGS2
CEACAM1
GRHL2
EPCAM
HRAS
TGFB2
TGFB3
F
MII vs MI Day6
S100A12
DCN
CCND2
TGFB2
EPCAM
EGF
ESRP1
IDO1
MSLN
FGFR3
FGFR2
GRHL2

Supplementary Figure S1: Profiling of mammary spheres growing for different time intervals

(**A**) Fluorescent imaging of engineered murine mammary tumor Py2T cells expressing red fluorescence protein (RFP) grown under 3D conditions for the indicated time periods. (**B**) Phase-contrast micrograph of daughter MII spheres left for two months before splitting (upper images) and after re-seeding into new wells. Images were taken at the indicated times. Scale bar, 100 μm in **A** and **B**. (**C - F**) Volcano plots showing the differential expression analysis of MII-spheres at Day6 against MII-spheres at Day3 (**C**), MI-spheres at Day6 against MI-spheres at Day3 (**D**), MII-spheres at Day3 versus MI-spheres at Day3 (**E**), and MII-spheres Day6 versus MI-spheres at Day6 (**F**). The X-axis indicates the log2 fold-change of the expressed genes and the Y-axis represents the -log10 values of the false discovery rate (FDR). The dotted horizontal line indicates the levels of statistical significance at 0.05 and the vertical lines represent log2 fold-change threshold ±1. Selected genes are highlighted in each plot.

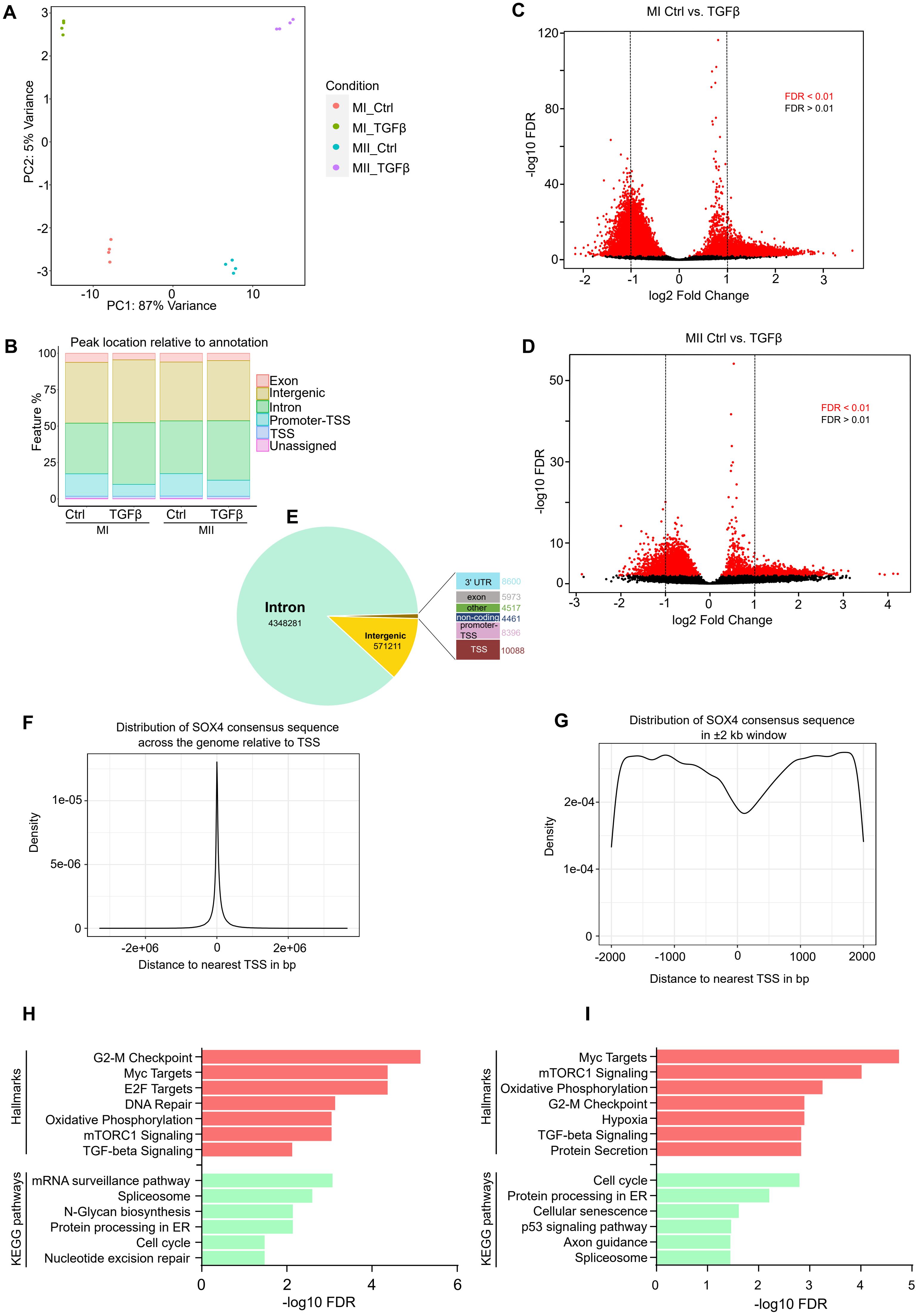

A
PC1: 87% Variance
PC2: 5% Variance
Condition
MI_Ctrl
MI_TGFβ
MII_Ctrl
MII_TGFβ
B
Peak location relative to annotation
Feature %
Exon
Intergenic
Intron
Promoter-TSS
TSS
Unassigned
Ctrl
TGFβ
Ctrl
TGFβ
MI
MII
C
MI Ctrl vs. TGFβ
-log10 FDR
log2 Fold Change
FDR < 0.01
FDR > 0.01
D
MII Ctrl vs. TGFβ
-log10 FDR
log2 Fold Change
FDR < 0.01
FDR > 0.01
E
Intron
4348281
Intergenic
571211
3' UTR 8600
exon 5973
other 4517
non-coding 4461
promoter-TSS 8396
TSS 10088
F
Distribution of SOX4 consensus sequence across the genome relative to TSS
Density
Distance to nearest TSS in bp
G
Distribution of SOX4 consensus sequence in ±2 kb window
Density
Distance to nearest TSS in bp
H
Hallmarks
G2-M Checkpoint
Myc Targets
E2F Targets
DNA Repair
Oxidative Phosphorylation
mTORC1 Signaling
TGF-beta Signaling
KEGG pathways
mRNA surveillance pathway
Spliceosome
N-Glycan biosynthesis
Protein processing in ER
Cell cycle
Nucleotide excision repair
-log10 FDR
I
Hallmarks
Myc Targets
mTORC1 Signaling
Oxidative Phosphorylation
G2-M Checkpoint
Hypoxia
TGF-beta Signaling
Protein Secretion
KEGG pathways
Cell cycle
Protein processing in ER
Cellular senescence
p53 signaling pathway
Axon guidance
Spliceosome
-log10 FDR

Supplementary Figure S2: Assessment of differential chromatin accessibility in TGFβ-stimulated spheres

(**A**) Principal component analysis in unstimulated (Ctrl) and TGFβ-stimulated conditions based on ATAC-seq analysis of MI- and MII-spheres. Four independent biological replicates were analyzed in each condition. Principal components (PC) 1 and 2 explain the percentage of variance between samples. (**B**) Cumulative bar plot showing the percentage of feature distribution of the total number of enriched peaks relative to the annotated genomic features. (**C, D**) Volcano plots showing the differentially accessible peaks (log2-fold change > ±1 and FDR < 0.01) in unstimulated MI (**C**) and MII (**D**) versus stimulated spheres. Different colors correspond to the level of statistical significance and dashed lines represent log2 fold-change threshold ±1. (**E**) Global distribution of SOX4 consensus binding elements across the human genome with the corresponding number of motifs. (**F**) Global distribution of SOX4 consensus binding elements relative to the annotated transcription start sites (TSS). (**G**) Distribution of SOX4 consensus binding elements within a window of ±2kb from the TSS. (**H, I**) Bar graphs showing the significantly enriched hallmarks and KEGG pathways based on the differentially accessible peaks mapped to the promoter-TSS regions of stimulated MI- (**H**) and MII-spheres (**I**) versus the corresponding controls.

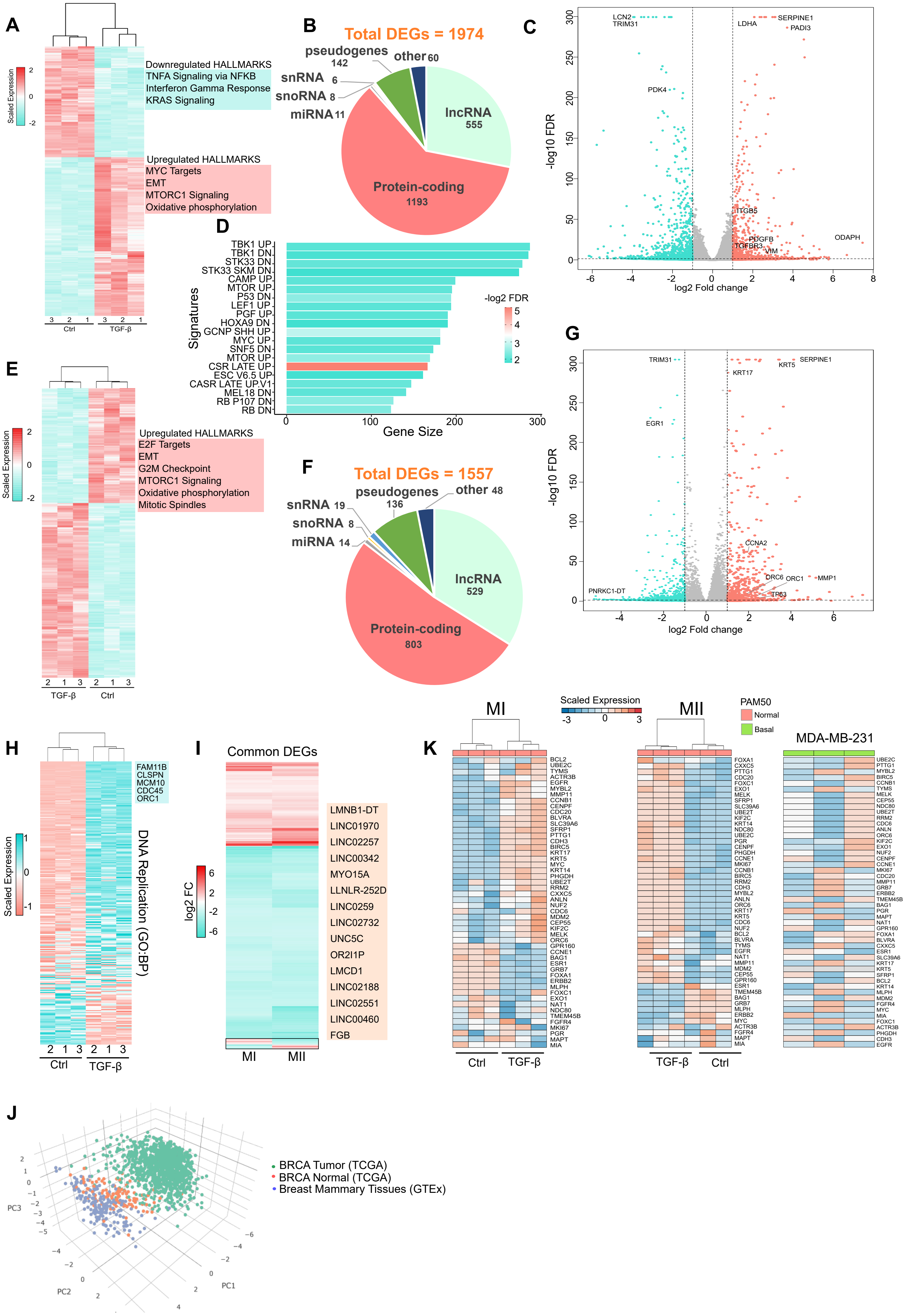

A
Downregulated HALLMARKS
TNFA Signaling via NFKB
Interferon Gamma Response
KRAS Signaling
Upregulated HALLMARKS
MYC Targets
EMT
MTORC1 Signaling
Oxidative phosphorylation
Scaled Expression
Ctrl
TGF-β
B
Total DEGs = 1974
pseudogenes 142
other 60
snRNA 6
snoRNA 8
miRNA 11
lncRNA 555
Protein-coding 1193
C
-log10 FDR
log2 Fold change
LCN2
TRIM31
SERPINE1
LDHA
PADI3
PDK4
ITGB5
PDGFB
TGFBR3
VIM
ODAPH
D
Signatures
TBK1 UP
TBK1 DN
STK33 DN
STK33 SKM DN
CAMP UP
MTOR UP
P53 DN
LEF1 UP
PGF UP
HOXA9 DN
GCNP SHH UP
MYC UP
SNF5 DN
MTOR UP
CSR LATE UP
ESC V6.5 UP
CASR LATE UP.V1
MEL18 DN
RB P107 DN
RB DN
Gene Size
-log2 FDR
E
Upregulated HALLMARKS
E2F Targets
EMT
G2M Checkpoint
MTORC1 Signaling
Oxidative phosphorylation
Mitotic Spindles
Scaled Expression
TGF-β
Ctrl
F
Total DEGs = 1557
pseudogenes 136
other 48
snRNA 19
snoRNA 8
miRNA 14
lncRNA 529
Protein-coding 803
G
-log10 FDR
log2 Fold change
TRIM31
SERPINE1
KRT5
KRT17
EGR1
CCNA2
ORC6
ORC1
MMP1
TP63
PNRKC1-DT
H
FAM11B
CLSPN
MCM10
CDC45
ORC1
DNA Replication (GO:BP)
Scaled Expression
Ctrl
TGF-β
I
Common DEGs
log2 FC
LMNB1-DT
LINC01970
LINC02257
LINC00342
MYO15A
LLNLR-252D
LINC0259
LINC02732
UNC5C
OR2I1P
LMCD1
LINC02188
LINC02551
LINC00460
FGB
MI
MII
K
MI
MII
Scaled Expression
PAM50
Normal
Basal
MDA-MB-231
Ctrl
TGF-β
J
BRCA Tumor (TCGA)
BRCA Normal (TCGA)
Breast Mammary Tissues (GTEx)
PC1
PC2
PC3

Supplementary Figure S3: Transcriptional rewiring in response to TGFβ stimulation

(**A**, **E**) Heatmaps elucidating the expression patterns of DEGs (log2 fold-change>±1 and FDR<0.05) in unstimulated (Ctrl) and stimulated MI- (**A**) or MII- (**E**) spheres based on RNA-seq analysis. The top significantly enriched hallmarks are highlighted (nominal p-value < 0.01 and FDR< 0.05). The color-coded scale represents the scaled expression values. (**B**, **F**) Pie charts showing the numbers of DEGs in MI- (**B**) or MII- (**F**) spheres corresponding to different RNA classes. (**C**, **G**) Volcano plots showing the DEGs in stimulated versus unstimulated MI- (**C**) or MII- (**G**) spheres. The vertical dashed lines represent the log2 fold-change cutoff threshold at ±1 and the horizontal dashed line represents the statistical significance threshold at a false discovery rate of 0.05. (**D**) Significantly enriched oncogenic signature in stimulated MI-spheres based on the GSEA. The X-axis represents the number of genes constituting each signature. The color-coded scale indicates the level of statistical significance. (**H**) Heatmap indicating the MII-sphere expression patterns of different genes constituting the DNA replication biological process extracted from the gene ontology collection (GO:BP). (**I**) Heatmap showing the RNA expression patterns of the commonly dysregulated genes in stimulated MI- and MII-spheres. The highlighted genes are the DEGs with opposite expression patterns in stimulated MI- and MII-spheres. (**K**) Heatmaps demonstrating the expression of genes comprising the PAM50 signature in MI- and MII-spheres in addition to the MDA-MB-231 triple-negative breast cancer cell line that serves as a positive control for the clustering analysis. The collective signature classified MI- and MII-cells as normal and MDA-MB-231 cells as basal. (**J**) Principal component analysis based on the combined signature of 16 genes in breast cancer tumor tissues (BRCA) derived from the TCGA dataset and normal mammary tissues derived from the GTEx dataset.

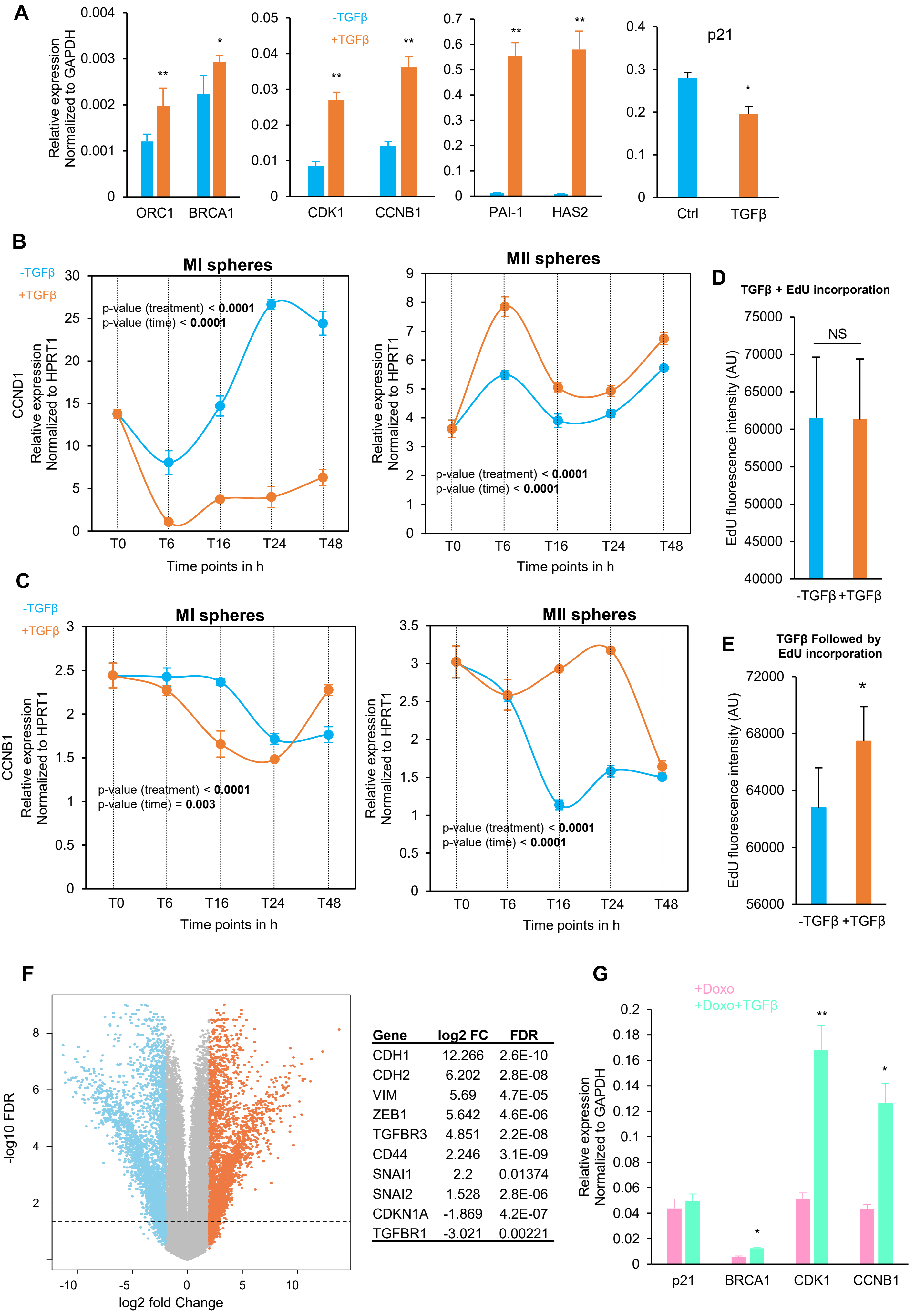

Gene	log2 FC	FDR
CDH1	12.266	2.6E-10
CDH2	6.202	2.8E-08
VIM	5.69	4.7E-05
ZEB1	5.642	4.6E-06
TGFBR3	4.851	2.2E-08
CD44	2.246	3.1E-09
SNAI1	2.2	0.01374
SNAI2	1.528	2.8E-06
CDKN1A	-1.869	4.2E-07
TGFBR1	-3.021	0.00221

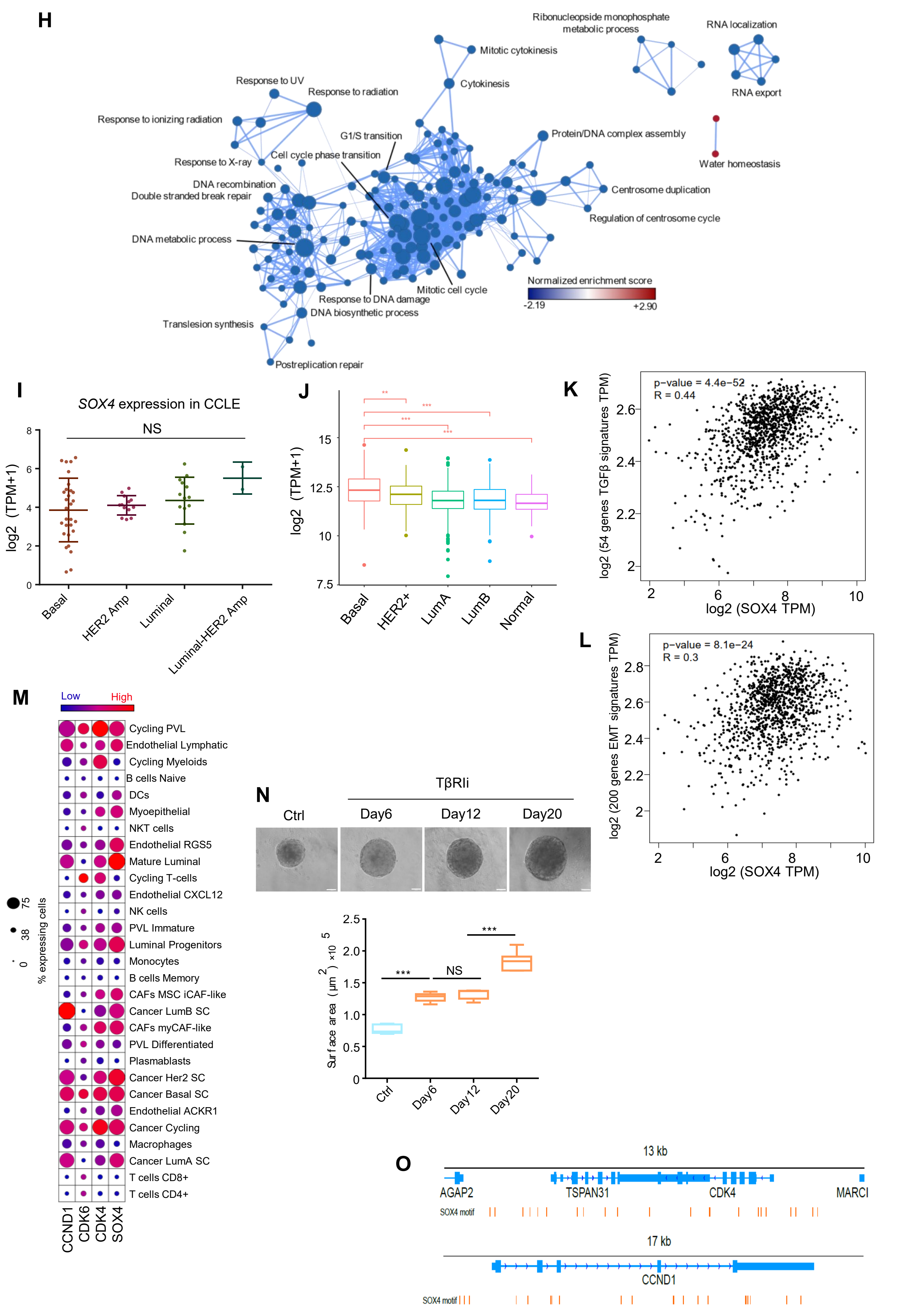

H
Ribonucleopside monophosphate metabolic process
RNA localization
Mitotic cytokinesis
RNA export
Cytokinesis
Response to UV
Response to radiation
Response to ionizing radiation
G1/S transition
Protein/DNA complex assembly
Water homeostasis
Response to X-ray
Cell cycle phase transition
DNA recombination
Double stranded break repair
Centrosome duplication
Regulation of centrosome cycle
DNA metabolic process
Normalized enrichment score
-2.19
+2.90
Mitotic cell cycle
Response to DNA damage
DNA biosynthetic process
Translesion synthesis
Postreplication repair
I
SOX4 expression in CCLE
NS
log2 (TPM+1)
Basal
HER2 Amp
Luminal
Luminal-HER2 Amp
J
log2 (TPM+1)
Basal
HER2+
LumA
LumB
Normal
K
p-value = 4.4e-52
R = 0.44
log2 (54 genes TGFβ signatures TPM)
log2 (SOX4 TPM)
L
p-value = 8.1e-24
R = 0.3
log2 (200 genes EMT signatures TPM)
log2 (SOX4 TPM)
M
Low
High
Cycling PVL
Endothelial Lymphatic
Cycling Myeloids
B cells Naive
DCs
Myoepithelial
NKT cells
Endothelial RGS5
Mature Luminal
Cycling T-cells
Endothelial CXCL12
NK cells
PVL Immature
Luminal Progenitors
Monocytes
B cells Memory
CAFs MSC iCAF-like
Cancer LumB SC
CAFs myCAF-like
PVL Differentiated
Plasmablasts
Cancer Her2 SC
Cancer Basal SC
Endothelial ACKR1
Cancer Cycling
Macrophages
Cancer LumA SC
T cells CD8+
T cells CD4+
CCND1
CDK6
CDK4
SOX4
% expressing cells
N
TβRIi
Ctrl
Day6
Day12
Day20
Surface area (μm²) ×10⁵
NS
O
13 kb
AGAP2
TSPAN31
CDK4
MARCI
SOX4 motif
17 kb
CCND1
SOX4 motif

Supplementary Figure S4: TGFβ signaling correlates with SOX4 and drives transcriptional reprogramming and chemoresistance in breast cancer cells

(**A**) RT-qPCR analysis of the indicated genes in unstimulated and TGFβ-stimulated MII-spheres following 16 h of treatment. (**B**) RT-qPCR analysis of *CCND1* and *CCNB1* expression in MI- and MII-spheres at different time points corresponding to 6 h (T6), 16 h (T16), 24 h (T24) and 48 h (T48) of TGFβ stimulation. The statistical significance was derived using Two-way ANOVA test defining column factor as treatment condition and row factor as time points with Sidak's correction for multiple comparisons. (**D**, **E**) Click-iT EdU incorporation assay measured by green fluorescence intensity in MII spheres either simultaneously stimulated with TGFβ and incubated with EdU for 16 h (**D**) or pre-stimulated with TGFβ for 16 h followed by EdU incorporation for 16 h (**E**). (**F**) Volcano plot demonstrating the differential gene expression analysis of doxorubicin-resistant MCF-7 cells versus the naïve cells. The dashed line indicates the level of statistical significance threshold at FDR of 0.05. Selected genes with their log2 fold-change and FDR values are highlighted in the table. (**G**) RT-qPCR analysis of the indicated genes in MII-spheres treated for 48 h with 500 nM doxorubicin alone or combined with TGFβ. Values in **A–C** and **G** represent mRNA expression levels normalized to *GAPDH* or *HPRT1*. Data are presented as mean values of at least 20 independent biological sphere replicates with three technical replicates ± SEM. Data in **D** and **E** are derived from five independent biological replicates. Statistical significance was derived using a two-tailed unpaired Student's *t*-test. (**H**) Visualization of interacting networks based on the REACTOME database in MDA-MB-231 cells transiently depleted of SMAD2/3/4 using siRNAs. The color-coded scale represents the normalized enrichment score values. Only statistically significant pathways with p-values < 0.005 are visualized. (**I**) Expression of *SOX4* mRNA levels in different breast cancer lines and the corresponding molecular subtypes present in the cancer cell line encyclopedia (CCLE). (**J**) Boxplots indicating the mRNA expression levels of *SOX4* in different breast cancer tissues

derived from the TCGA dataset. The mRNA levels in **I** and **J** are expressed as a log2 scale of normalized transcript per million (TPM+1). Statistical significance was derived using a one-way ANOVA test with Tukey's correction for multiple comparisons. (**K, L**) Scatter plots representing the correlation between *SOX4* mRNA expression levels and the collective signatures of TGFβ (**K**) and EMT (**L**) in breast cancer tissues of the TCGA dataset. (**M**) Dot plot visualizing the mRNA expression levels of the indicated genes in different cell types based on the scRNA-seq analysis of breast cancer tissues. (**N**) Phase contrast micrographs of MII-spheres treated either with DMSO, as a control (Ctrl), or TGFβ receptor type I inhibitor (TβRIi) for the indicated time intervals (upper panel) and the corresponding quantification of the surface area derived from six independent measurements is shown in the lower panel as a box plot with median values and whiskers representing minimum and maximum values. (Scale bar, 100 μm). (**O**) Visualization of the SOX4 consensus motifs present within the vicinity of the *CDK4* and *CCND1* loci. In **A**, **D**, **E**, **G, J** and **N**, p-values $*p \leq 0.05$, $**p \leq 0.01$, $***p \leq 0.001$, NS, not significant.

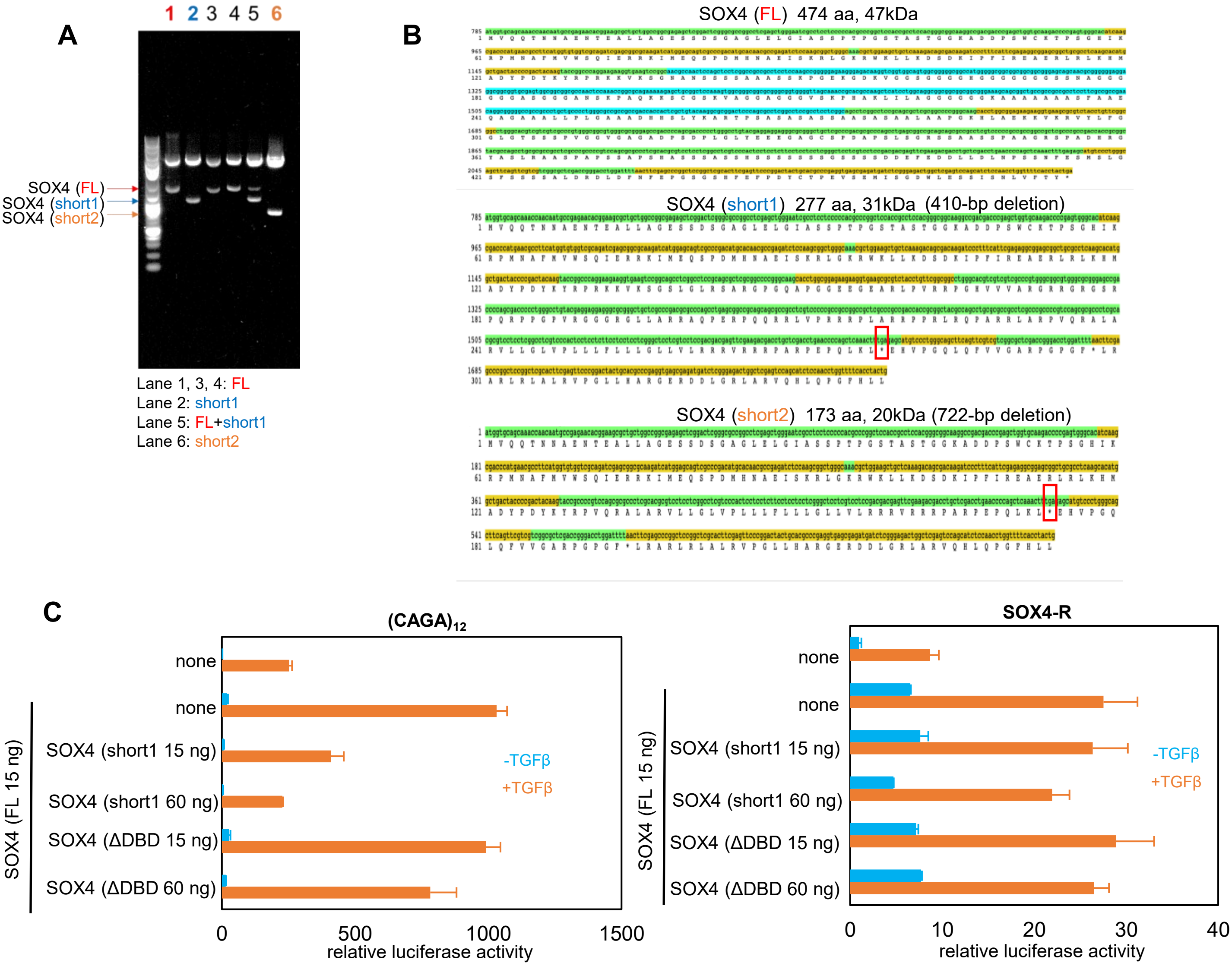
A
1 2 3 4 5 6
SOX4 (FL)
SOX4 (short1)
SOX4 (short2)
Lane 1, 3, 4: FL
Lane 2: short1
Lane 5: FL+short1
Lane 6: short2
B
SOX4 (FL) 474 aa, 47kDa
SOX4 (short1) 277 aa, 31kDa (410-bp deletion)
SOX4 (short2) 173 aa, 20kDa (722-bp deletion)
C
(CAGA)12
none
none
SOX4 (short1 15 ng)
SOX4 (short1 60 ng)
SOX4 (ΔDBD 15 ng)
SOX4 (ΔDBD 60 ng)
SOX4 (FL 15 ng)
-TGFβ
+TGFβ
0
500
1000
1500
relative luciferase activity
SOX4-R
none
none
SOX4 (short1 15 ng)
SOX4 (short1 60 ng)
SOX4 (ΔDBD 15 ng)
SOX4 (ΔDBD 60 ng)
SOX4 (FL 15 ng)
-TGFβ
+TGFβ
0
10
20
30
40
relative luciferase activity

Supplementary Figure S5: Cloning and expression of SOX4 isoforms

(**A**) DNA gel electrophoresis of PCR-amplified *SOX4* cDNA obtained from the glioblastoma cell line U2987MG. The arrows indicate the full-length (FL) transcript and the two shorter isoforms denoted as short1 and short2. (**B**) The DNA sequences and the corresponding amino acids of *SOX4* FL and shorter isoforms obtained by Sanger sequencing. The DNA fragment of *SOX4* FL highlighted in blue is deleted in the shorter isoforms with pre-mature stop codons marked by asterisks and red rectangles. (**C**) Luciferase reporter assays using the $(CAGA)_{12}$ reporter (left panel) and *SOX4*-R reporter (right panel) in A549 cells co-transfected with 15 ng of SOX4 FL vector and the indicated expression constructs in the presence or absence of TGFβ stimulation. Data are presented as mean values of two biological replicates with three technical replicates ± SEM.

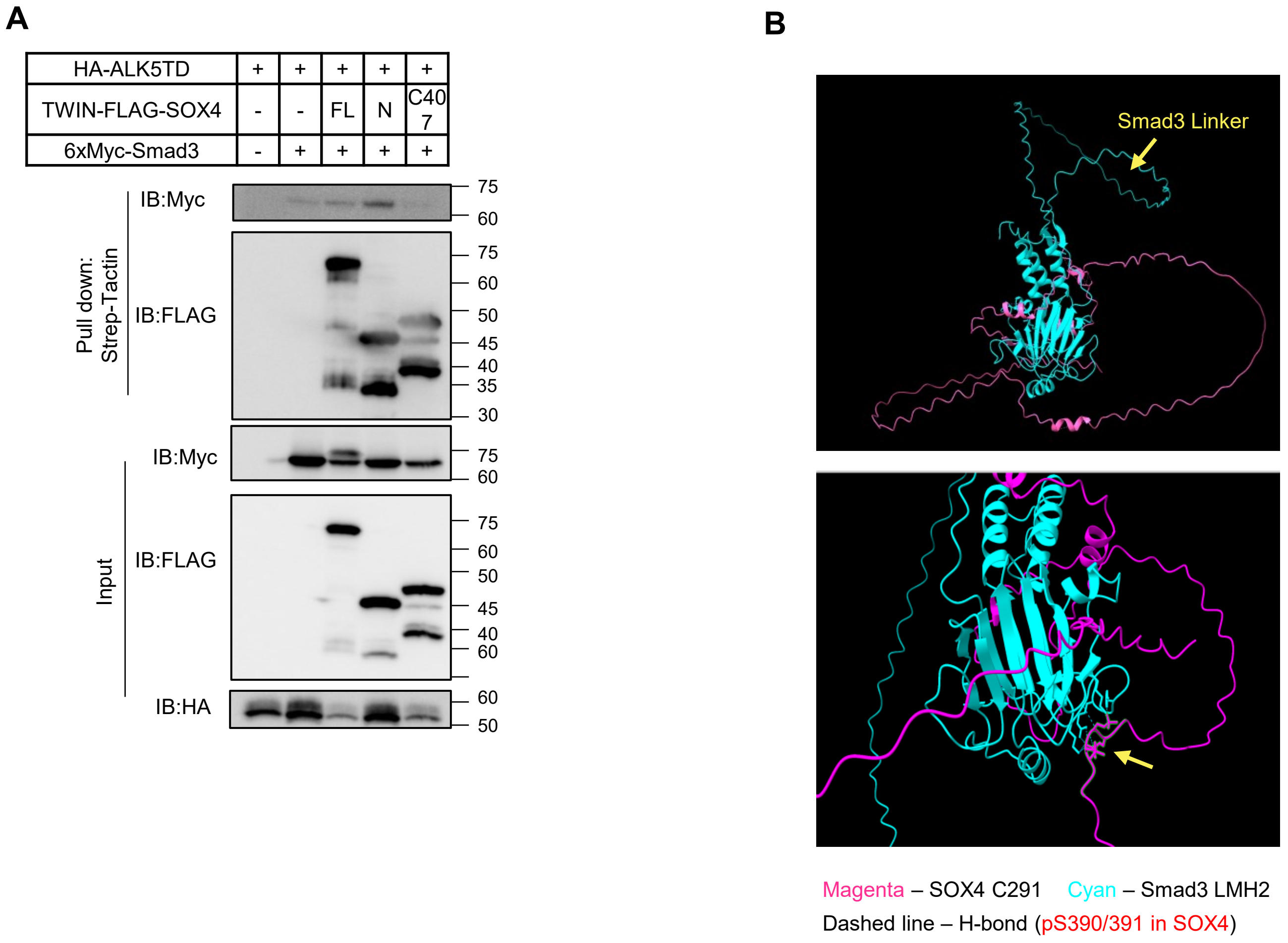

A
HA-ALK5TD
TWIN-FLAG-SOX4
6xMyc-Smad3
FL
N
C40
7
Pull down:
Strep-Tactin
IB:Myc
IB:FLAG
Input
IB:Myc
IB:FLAG
IB:HA
75
60
50
45
40
35
30
B
Smad3 Linker
Magenta – SOX4 C291 Cyan – Smad3 LMH2
Dashed line – H-bond (pS390/391 in SOX4)

Supplementary Figure S6: Characterization of the interaction surface of the SOX4/SMAD3 complex

(**A**) Co-immunoprecipitation of TWIN-FLAG-tagged constructs expressing SOX4 full-length (FL), N-terminal domain (N) or C-terminal domain starting from amino acid residue 407 (C407), co-trasnfected with 6×Myc-tagged construct expressing SMAD3 full-length followed by immunoblotting (IB) in HEK293T cells expressing constitutively active HA-tagged TβRI (ALK5TD). (**B**) AlphaFold3 modeling of the interaction between the C-terminus of SOX4 starting at amino acid residue 291 (C291) and the SMAD3 C-terminal part including the linker and the MH2 domain (LMH2). The yellow arrow indicates predicted phosphorylation sites on SOX4 (S390/391).

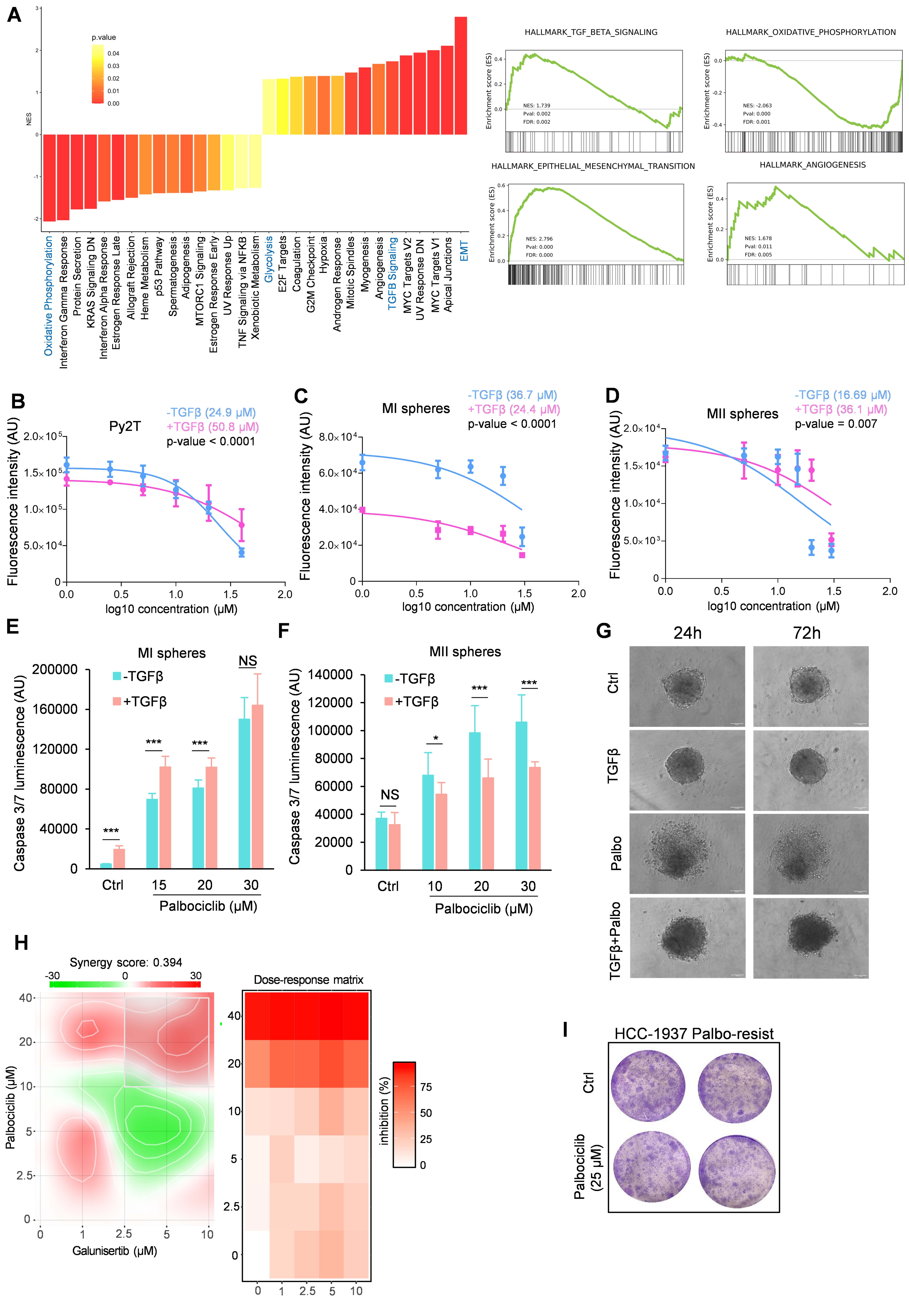
A
p.value
NES
Oxidative Phosphorylation
Interferon Gamma Response
Protein Secretion
KRAS Signaling DN
Interferon Alpha Response
Estrogen Response Late
Allograft Rejection
Heme Metabolism
p53 Pathway
Spermatogenesis
Adipogenesis
MTORC1 Signaling
Estrogen Response Early
UV Response Up
TNF Signaling via NFKB
Xenobiotic Metabolism
Glycolysis
E2F Targets
Coagulation
G2M Checkpoint
Hypoxia
Androgen Response
Mitotic Spindles
Myogenesis
Angiogenesis
TGFB Signaling
MYC Targets V2
UV Response DN
MYC Targets V1
Apical Junctions
EMT
HALLMARK_TGF_BETA_SIGNALING
NES: 1.739
Pval: 0.002
FDR: 0.002
HALLMARK_OXIDATIVE_PHOSPHORYLATION
NES: -2.063
Pval: 0.000
FDR: 0.001
HALLMARK_EPITHELIAL_MESENCHYMAL_TRANSITION
NES: 2.796
Pval: 0.000
FDR: 0.000
HALLMARK_ANGIOGENESIS
NES: 1.678
Pval: 0.011
FDR: 0.005
Enrichment score (ES)
B
Py2T
-TGFβ (24.9 μM)
+TGFβ (50.8 μM)
p-value < 0.0001
Fluorescence intensity (AU)
log10 concentration (μM)
C
MI spheres
-TGFβ (36.7 μM)
+TGFβ (24.4 μM)
p-value < 0.0001
D
MII spheres
-TGFβ (16.69 μM)
+TGFβ (36.1 μM)
p-value = 0.007
E
MI spheres
-TGFβ
+TGFβ
Caspase 3/7 luminescence (AU)
Ctrl
Palbociclib (μM)
NS
F
MII spheres
G
24h
72h
Ctrl
TGFβ
Palbo
TGFβ+Palbo
H
Synergy score: 0.394
Palbociclib (μM)
Galunisertib (μM)
Dose-response matrix
inhibition (%)
I
HCC-1937 Palbo-resist
Ctrl
Palbociclib (25 μM)

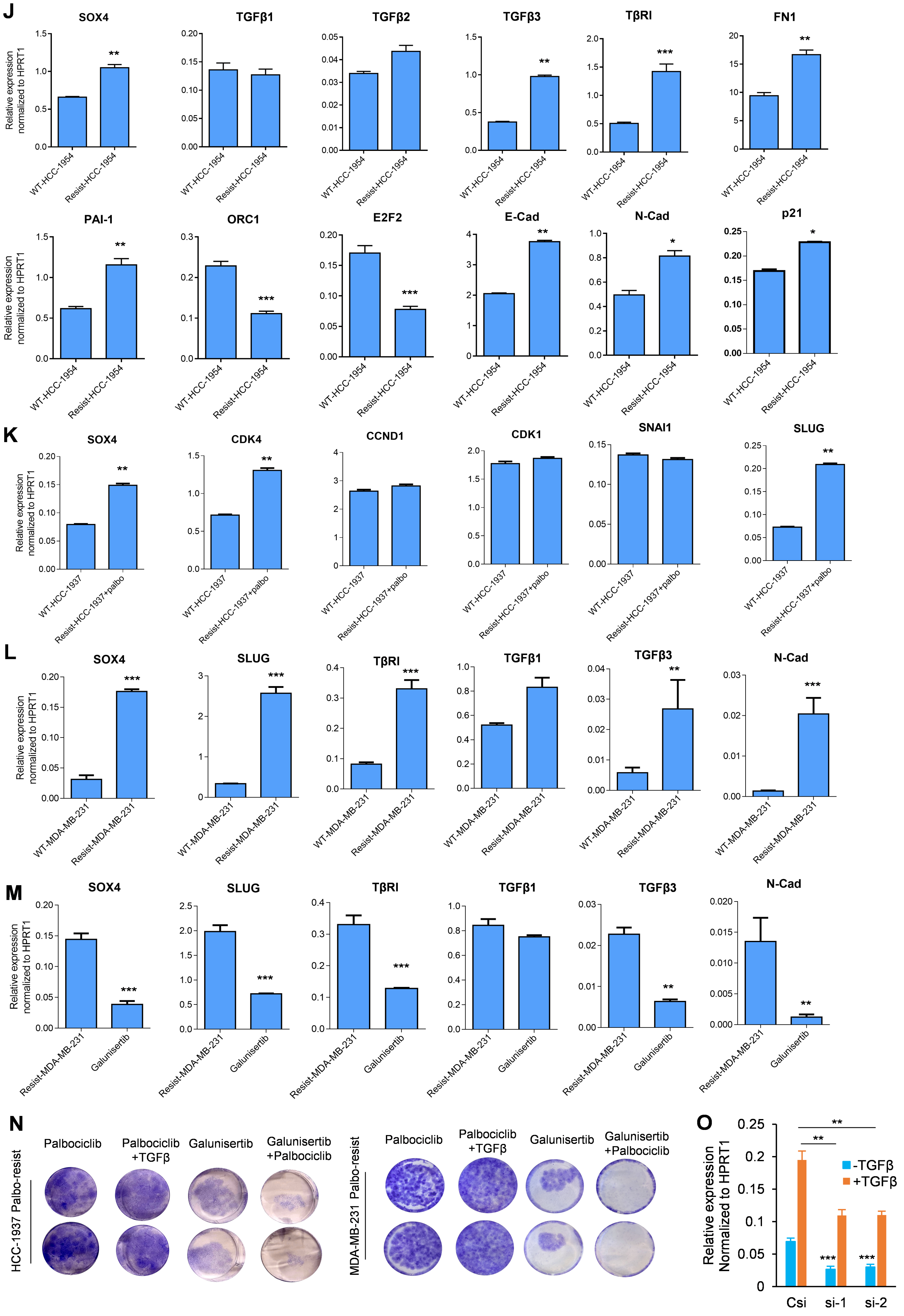
J
SOX4
TGFβ1
TGFβ2
TGFβ3
TβRI
FN1
PAI-1
ORC1
E2F2
E-Cad
N-Cad
p21
Relative expression normalized to HPRT1
WT-HCC-1954
Resist-HCC-1954
K
SOX4
CDK4
CCND1
CDK1
SNAI1
SLUG
WT-HCC-1937
Resist-HCC-1937+palbo
L
SOX4
SLUG
TβRI
TGFβ1
TGFβ3
N-Cad
WT-MDA-MB-231
Resist-MDA-MB-231
M
SOX4
SLUG
TβRI
TGFβ1
TGFβ3
N-Cad
Resist-MDA-MB-231
Galunisertib
N
Palbociclib
Palbociclib +TGFβ
Galunisertib
Galunisertib +Palbociclib
HCC-1937 Palbo-resist
MDA-MB-231 Palbo-resist
O
Relative expression Normalized to HPRT1
-TGFβ
+TGFβ
Csi
si-1
si-2

Supplementary Figure S7: TGFβ signaling attenuates the cytotoxic effect of palbociclib in breast cancer cells

(**A**) Enrichment plots indicating the significantly dysregulated hallmarks associated with palbociclib resistance in 1,092 cancer cell lines. NES stands for normalized enrichment score. (**B–D**) A dose-response curve of murine mammary Py2T (**B**), MI-spheres (**C**) and MII-spheres (**D**) exposed to palbociclib for 72 h in the presence or absence of TGFβ. Statistical significance was derived using a one-way ANOVA test with Tukey's correction for multiple comparisons utilizing non-linear regression analysis. Data are presented as mean values of eight biological replicates ± SEM. Statistical significance was derived using a two-tailed unpaired Student's *t*-test. (**E, F**) Caspase 3/7 activity measured by luminescence in MI- (**E**) and MII-spheres (**F**) treated with an escalating dose of palbociclib for 72 h in the presence or absence of TGFβ. (**G**) Phase-contrast micrographs of MII-spheres exposed to the indicated treatments for 24 and 72 h. The combined treatment of TGFβ and palbociclib was done simultaneously. Scale bars is = 100 µm. (**H**) Synergy and dose-response matrix heatmaps showing the effect of combining different concentrations of palbociclib and galunisertib on the viability of HCC-1954 cells following 72 h of treatment. (**I**) Colony-forming assay in resistant HCC-1937 (palbo-resist) cells either unexposed (Ctrl) or exposed to 25 µM of palbociclib for ten days. (**J–L**) RT-qPCR analysis of the indicated genes in resistant HCC-1954 (**J**), HCC-1937 (**K**), MDA-MB-231 cells (**L**) and the corresponding wildtype (WT) cells. (**M**) RT-qPCR analysis of the indicated genes in resistant MDA-MB-231 cells treated with 5 µM of galunisertib for 72 h. Values represent mRNA expression levels normalized to *HPRT1*. Data are presented as mean values of three technical replicates ± SD. Statistical significance was derived using a two-tailed unpaired Student's *t*-test. (**N**) Colony-forming assay with resistant HCC-1937 cells (left panel) and resistant MDA-MB-231 cells (right panel) exposed to the indicated treatments for two weeks. The medium was replenished twice a week with 2 ng/ml of TGFβ, 3 µM of galunisertib, 25 µM

of palbociclib in case of HCC-1937 resistant cells and 4 μM of palbociclib for MDA-MB-231 resistant cells. (**O**) qRT-PCR of MDA-MB-231 resistant cells transiently transfected for 48 h with scramble siRNA (Csi) or two siRNAs targeting SOX4 (si-1 and si-2) in the presence or absence of TGFβ incubated for 24 h. In **E, F**, **J–M and O**, p-values *p≤ 0.05, **p≤ 0.01, ***p≤ 0.001.

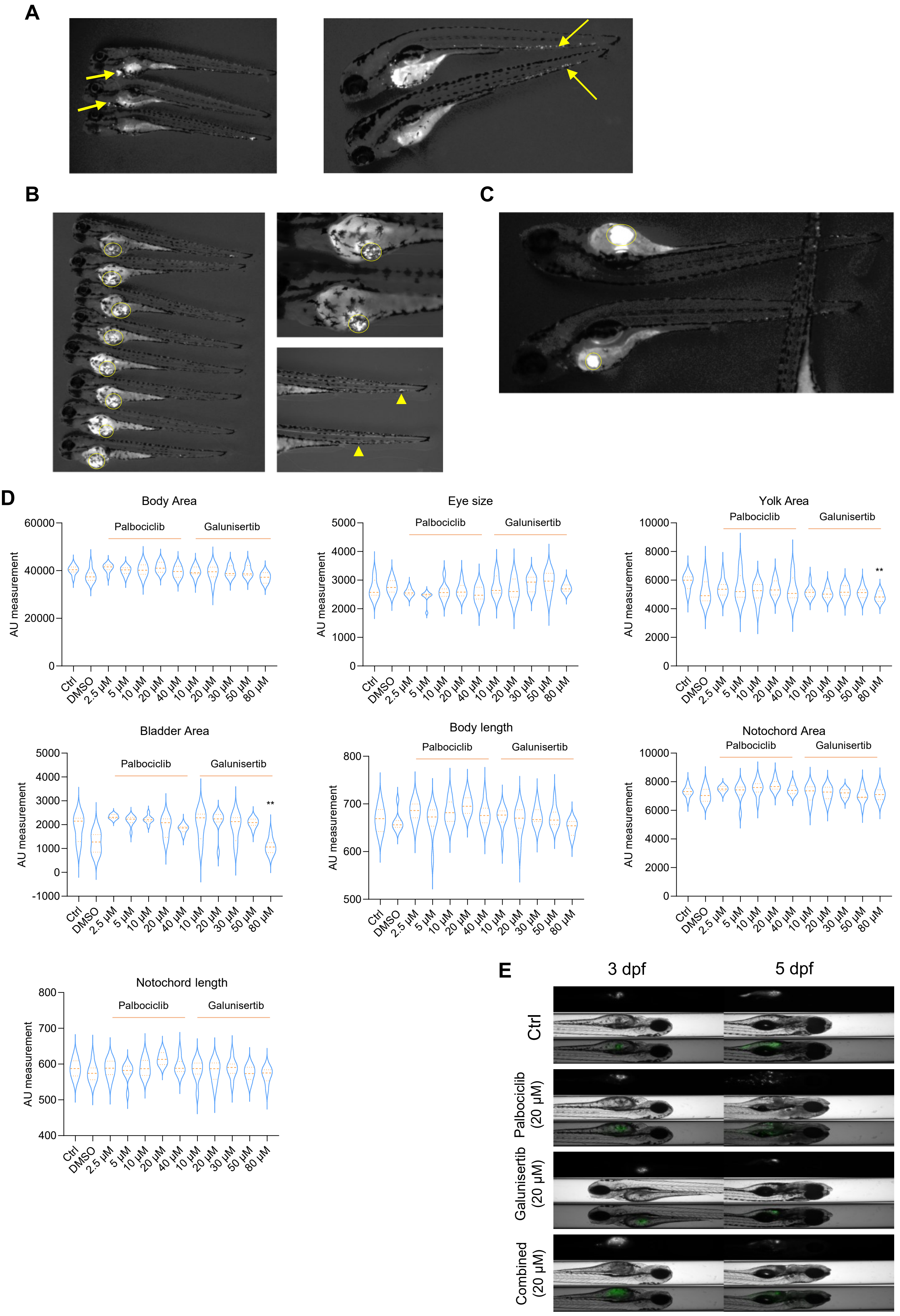

A
B
C
D
Body Area
Eye size
Yolk Area
Bladder Area
Body length
Notochord Area
Notochord length
AU measurement
Palbociclib
Galunisertib
Ctrl
DMSO
2.5 µM
5 µM
10 µM
20 µM
40 µM
10 µM
20 µM
30 µM
50 µM
80 µM
**
E
3 dpf
5 dpf
Ctrl
Palbociclib (20 µM)
Galunisertib (20 µM)
Combined (20 µM)

Supplementary Figure S8: TGFβ inhibition suppresses tumor growth in a zebrafish model

(**A**) Zebrafish embryos injected 2 days post-fertilization (2 dpf) with resistant HCC-1937 cells into the bloodstream. The yellow arrow indicates the fluorescently labeled cancer cells (left panel) and migratory cancer cells circulating the blood stream post-injection. (**B**) Zebrafish embryos injected with resistant HCC-1937 cells into the perivitelline space (PVS). The yellow circles mark cancer cells in close proximity to melanocytes. The triangles indicate the migrating cancer cells. (**C**) Zebrafish embryos injected with resistant HCC-1937 cells into the yolk sack, forming tumor masses. (**D**) Violin plots measuring the indicated parameters in embryos exposed either to palbociclib or galunisertib at the indicated concentrations for 48 h. Unexposed (Ctrl) and DMSO-treated embryos served as controls. Statistical significance was derived using a one-way ANOVA test with Tukey's correction for multiple comparisons. p-values *$p \leq 0.05$, **$p \leq 0.01$, ***$p \leq 0.001$. (**E**) Zebrafish embryos exposed to DMSO (Ctrl) and the indicated treatments. Images of the same embryos were acquired at the indicated time intervals.

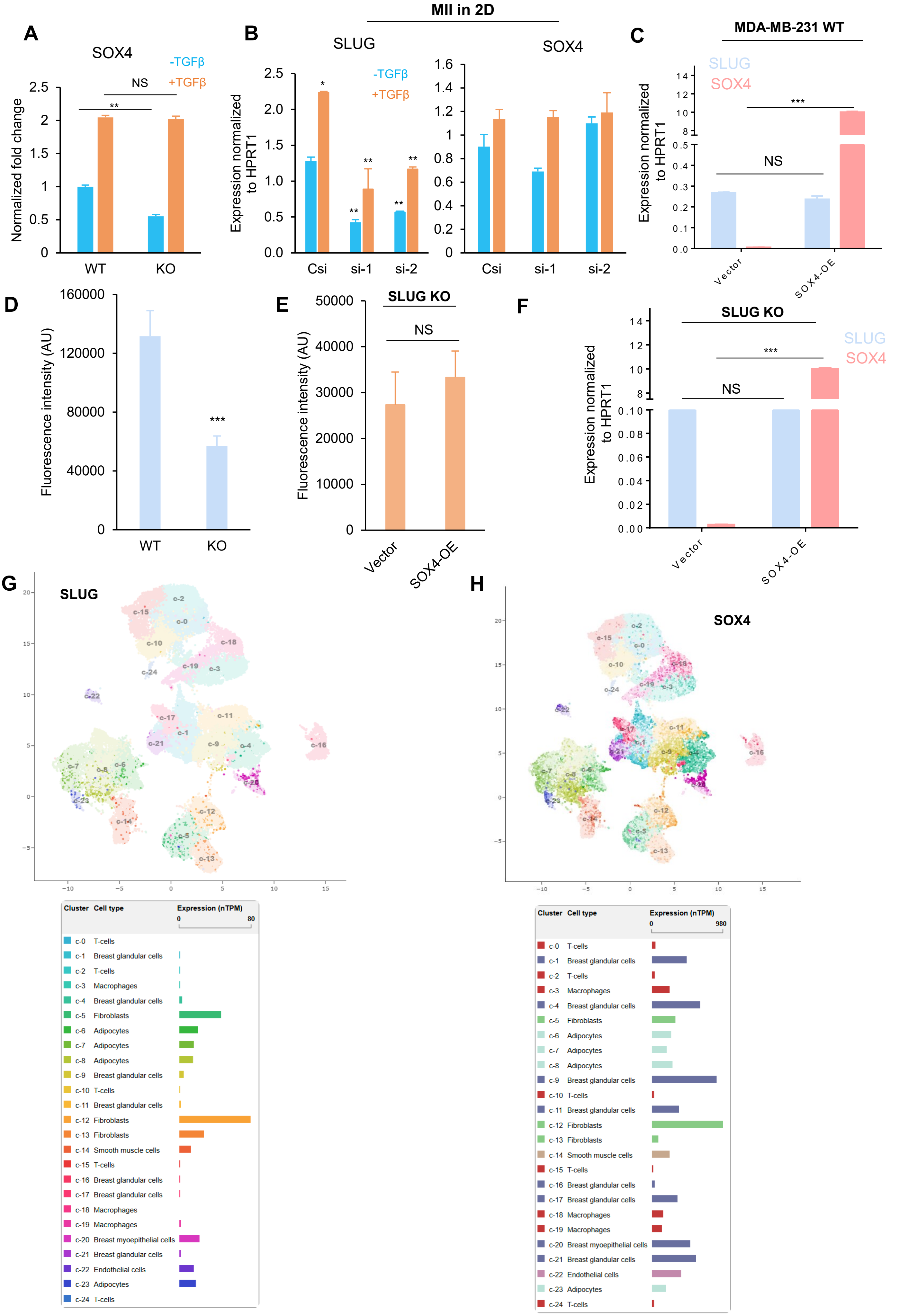
MII in 2D
A
SOX4
-TGFβ
+TGFβ
NS
**
Normalized fold change
WT
KO
B
SLUG
SOX4
Expression normalized to HPRT1
*
**
Csi
si-1
si-2
C
MDA-MB-231 WT
SLUG
SOX4
***
NS
Vector
SOX4-OE
D
Fluorescence intensity (AU)
***
WT
KO
E
SLUG KO
NS
Fluorescence intensity (AU)
Vector
SOX4-OE
F
SLUG KO
SLUG
SOX4
***
NS
Expression normalized to HPRT1
Vector
SOX4-OE
G
SLUG
H
SOX4
Cluster Cell type Expression (nTPM)
c-0 T-cells
c-1 Breast glandular cells
c-2 T-cells
c-3 Macrophages
c-4 Breast glandular cells
c-5 Fibroblasts
c-6 Adipocytes
c-7 Adipocytes
c-8 Adipocytes
c-9 Breast glandular cells
c-10 T-cells
c-11 Breast glandular cells
c-12 Fibroblasts
c-13 Fibroblasts
c-14 Smooth muscle cells
c-15 T-cells
c-16 Breast glandular cells
c-17 Breast glandular cells
c-18 Macrophages
c-19 Macrophages
c-20 Breast myoepithelial cells
c-21 Breast glandular cells
c-22 Endothelial cells
c-23 Adipocytes
c-24 T-cells

Supplementary Figure S9: Analysis of SLUG as a potential regulator of SOX4 expression

(**A**) RT-qPCR analysis of *SOX4* expression levels in wildtype (WT) and *SLUG* KO (KO) MDA-MB-231 cells in the presence or absence of TGFβ treatment for 24 h. (**B**) Expression analysis of *SLUG* and *SOX4* mRNA levels in MII-cells transiently depleted of *SLUG* using two different siRNAs (si-1 and si-2) in the presence or absence of TGFβ treatment for 24 h. (**C**) Analysis of *SLUG* and *SOX4* levels in MDA-MB-231 cells transiently transfected for 48 h with an empty vector or SOX4 overexpression vector (SOX4-OE). (**D**) Viability assay in WT MDA-MB-231 and *SLUG* KO cells. (**E**) Viability assay in *SLUG* KO cells transiently transfected with an empty vector or SOX4-OE. Data are presented as mean values of three biological replicates ± SEM. p-values ***p≤ 0.001. (**F**) RT-qPCR analysis of *SLUG* and *SOX4* expression levels in *SLUG* KO cells transiently transfected for 48 h with an empty vector or SOX4-OE. Values represent mRNA expression levels normalized to *HPRT1*. Data in **A−C** and **F** are presented as mean values of three technical replicates ± SD. p-values *p≤ 0.05, **p≤ 0.01, ***p≤ 0.001. (**G, H**) UMAP plots visualizing the scaled expression levels of *SLUG* and *SOX4* in different cell populations based on scRNA-seq analysis of normal breast tissues obtained from the Protein Atlas database. The mRNA levels are expressed as normalized transcript per million (nTPM) values.

Fig. 2G

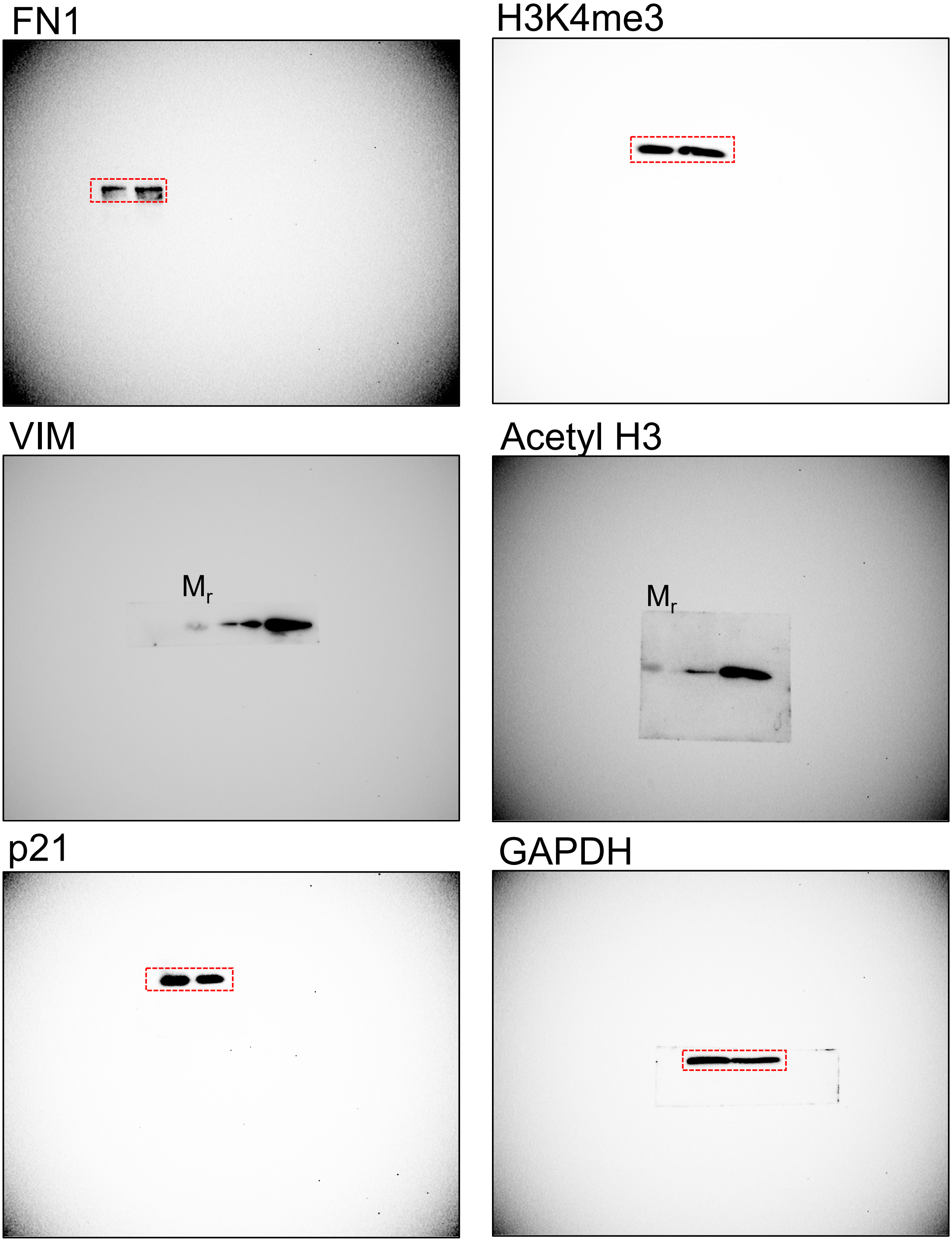

Suppl Fig.S10

Fig. 6A

IB: SMAD3/Pull down FLAG

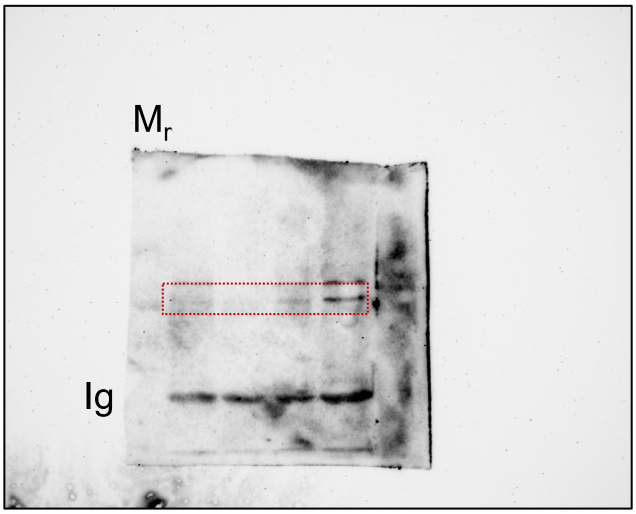

IB: SMAD3/Input

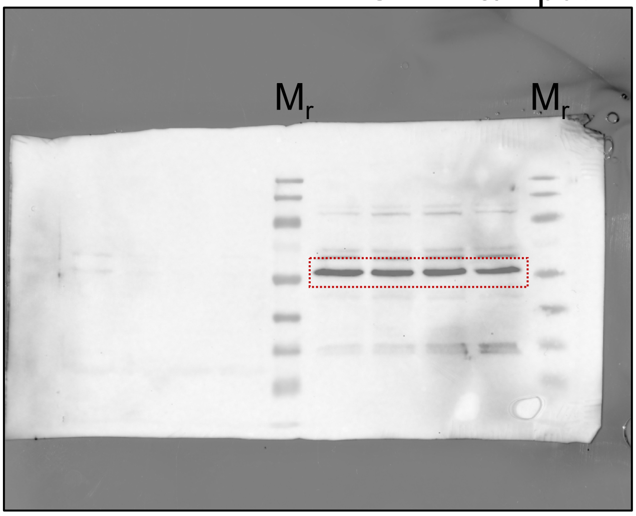

IB: SMAD4

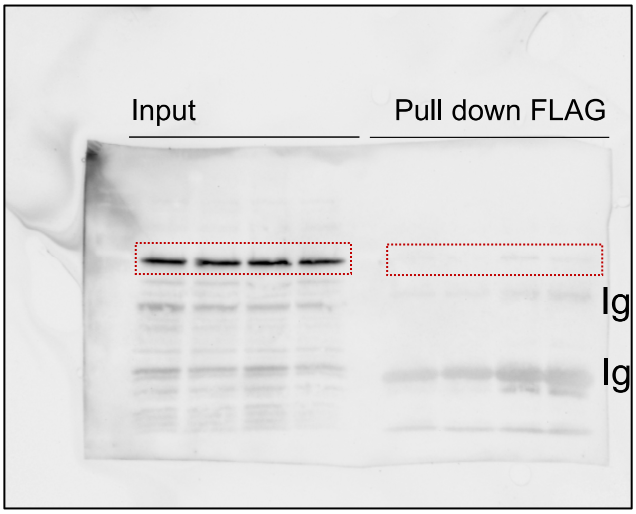

IB: FLAG (SOX4)

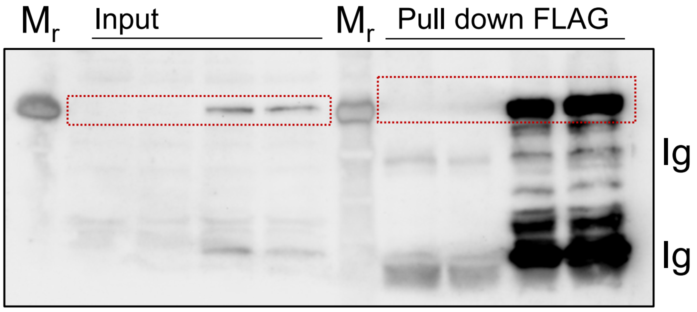

Fig. 6B

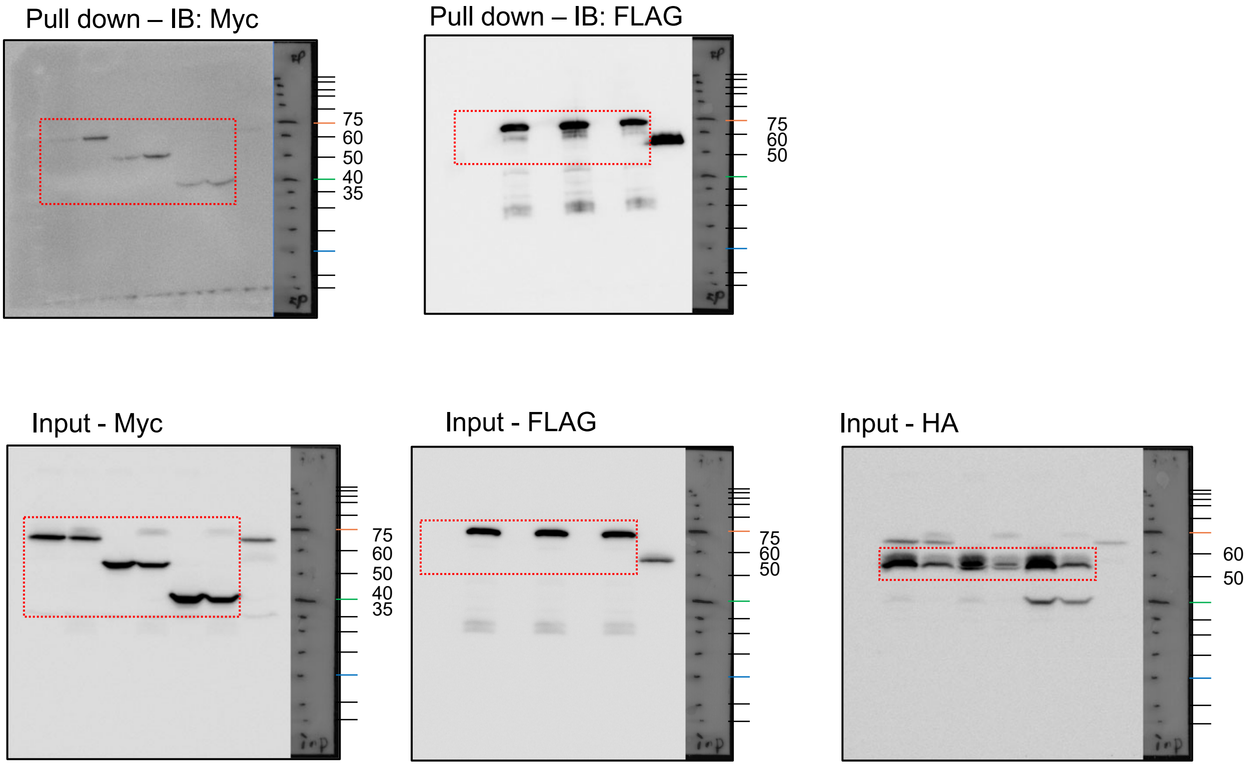

Fig. 6C

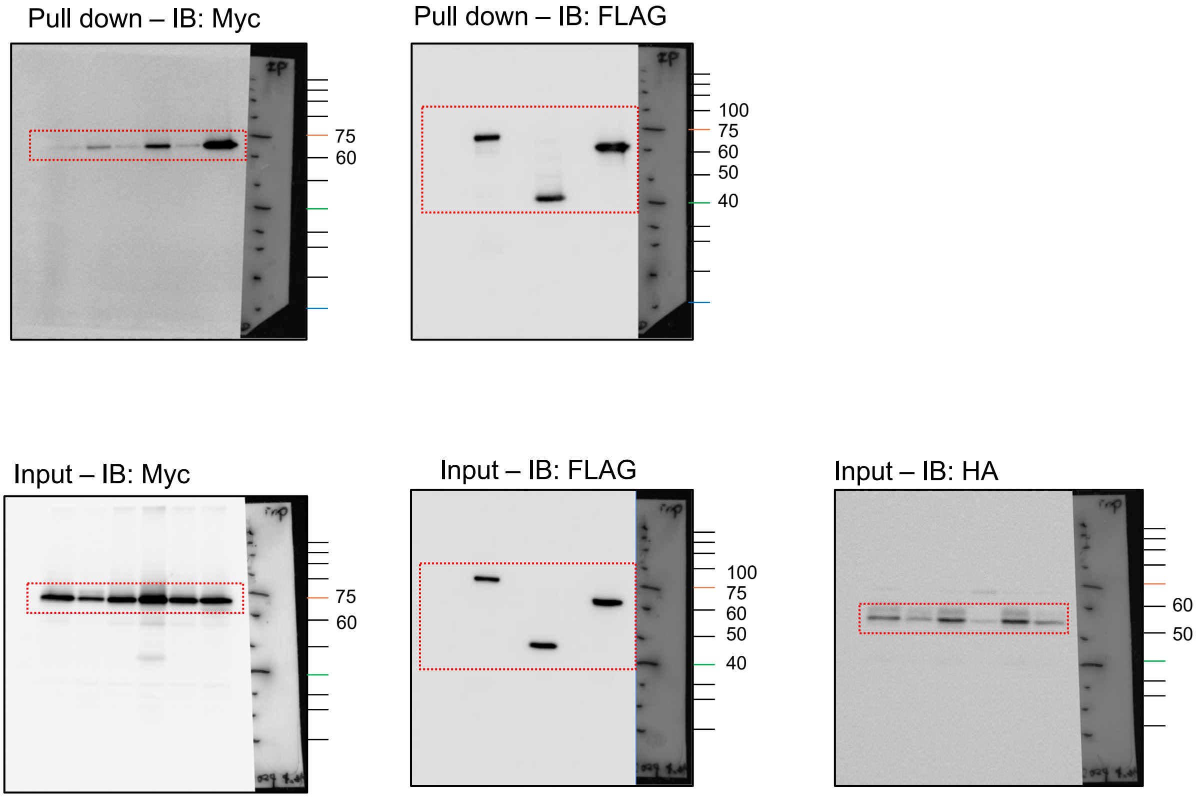

Suppl Fig.S10

Fig. 6F

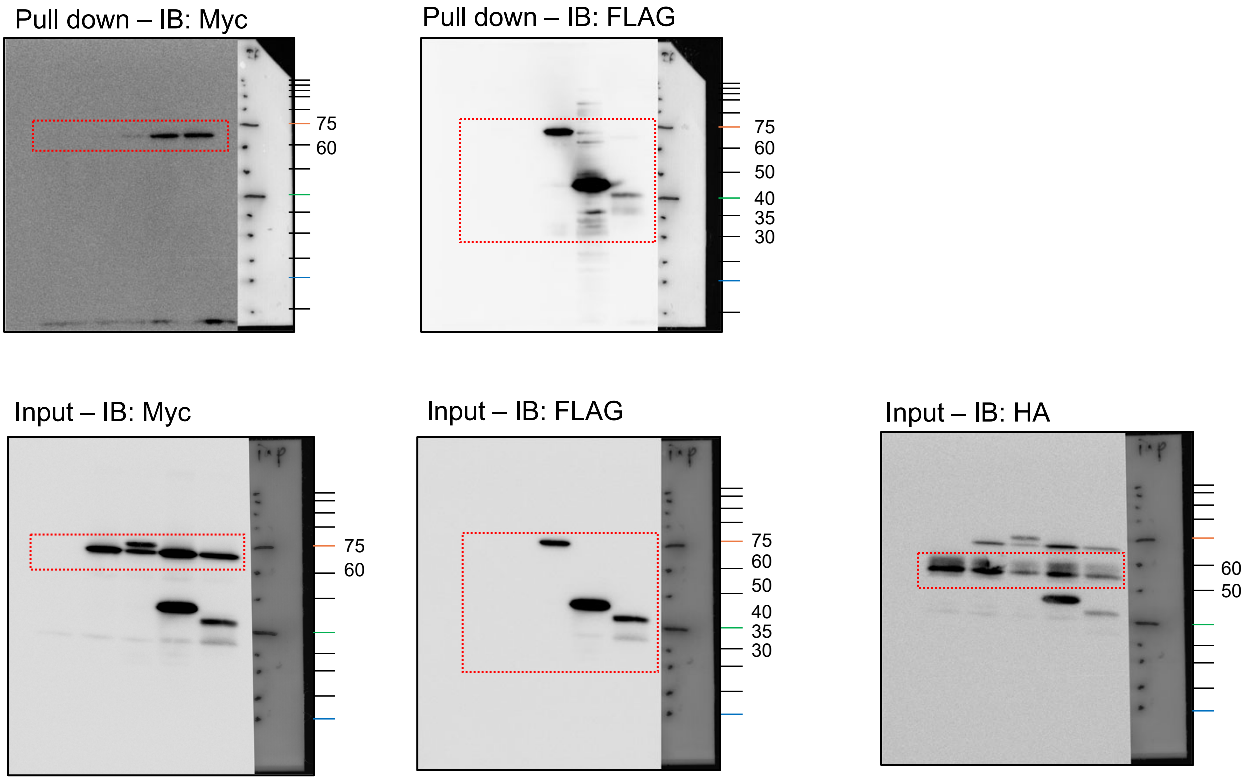

Fig. 6G

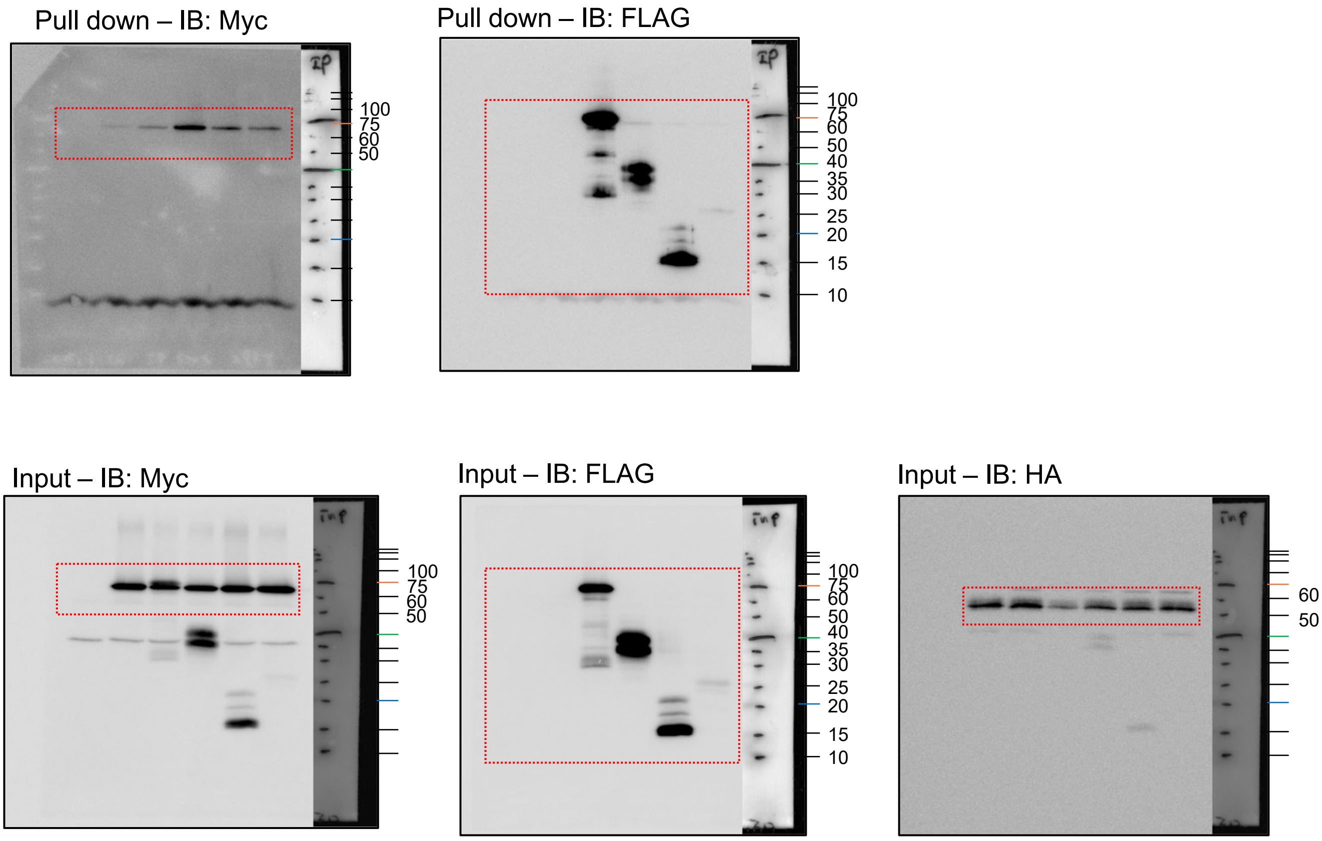

Suppl Fig.S10

Fig. 8J

H3K4me3

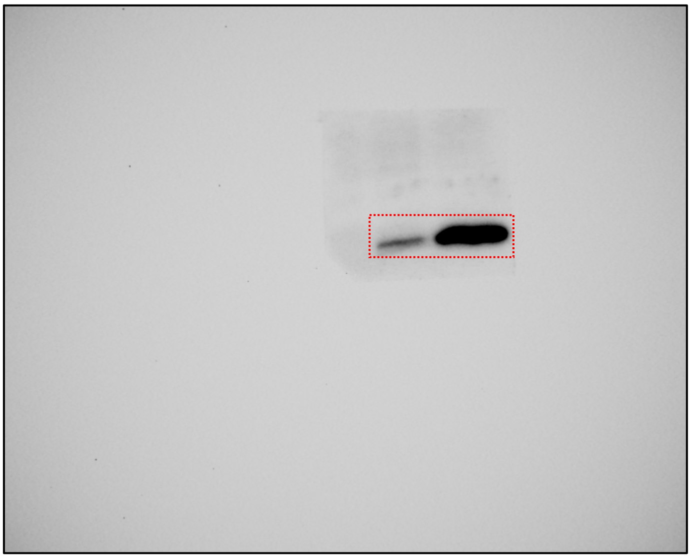

GAPDH

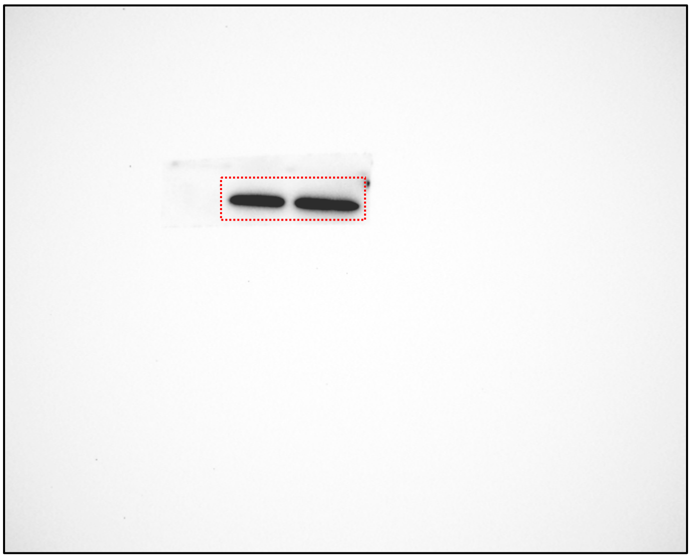

p-ɣH2A.X

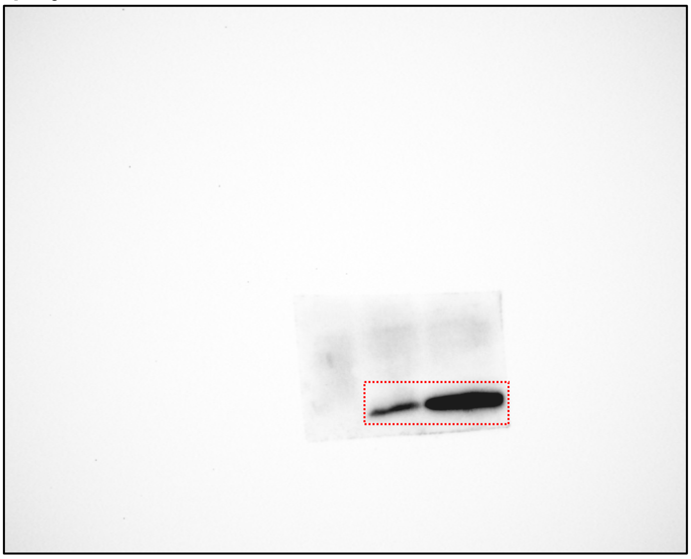

Suppl Fig.S10

Fig. S6A

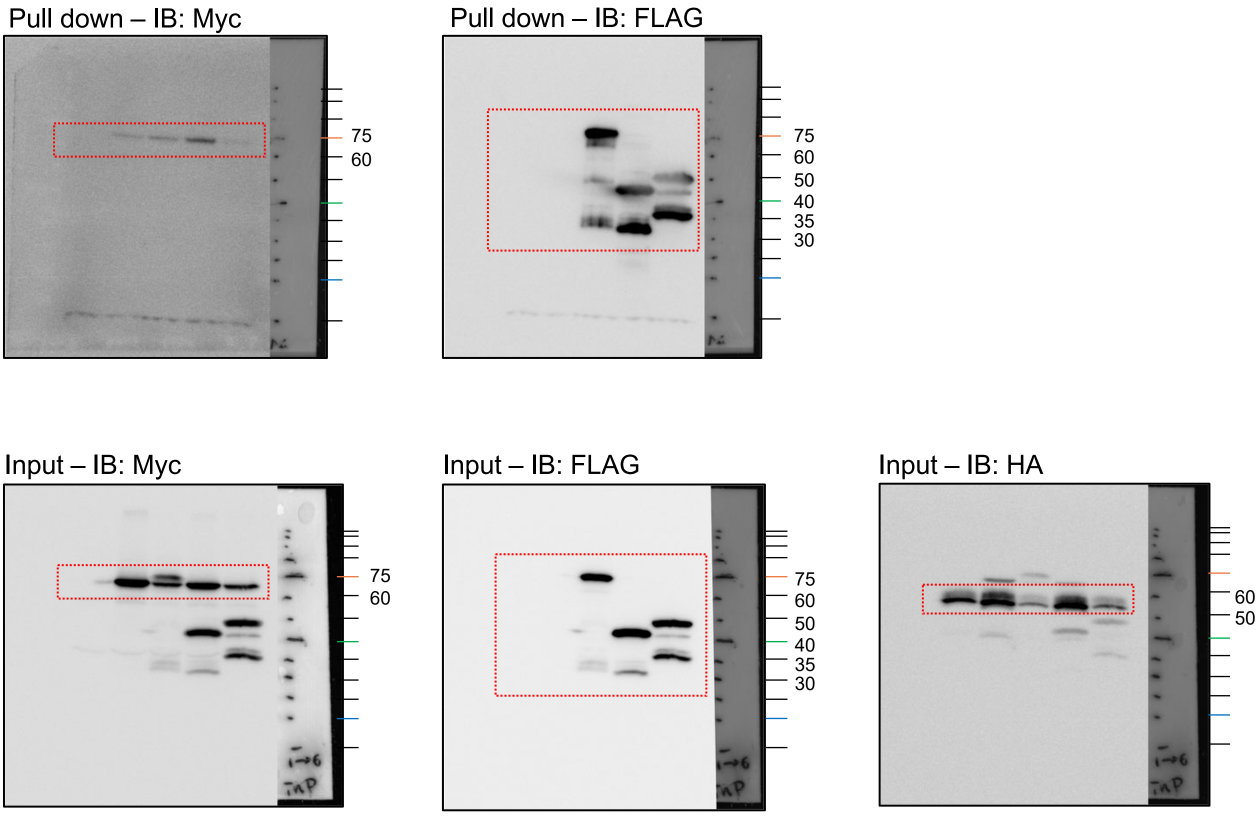

Suppl Fig.S10

Supplementary Figure S10: Unprocessed immunoblots

Unprocessed immunoblots for the indicated proteins along with molecular mass markers (in some of the immunoblots) and the corresponding figures. Dotted rectangles demarcate the cropped immunoblots presented in the main or supplementary figures, as indicated in each figure panel. In the case of immunoprecipitation, Ig indicates the immunoglobulin band and M_r stands for molecular mass marker.
